# Preliminary Evidence of the Use of Generative AI in Health Care Clinical Services: Systematic Narrative Review

**DOI:** 10.2196/52073

**Published:** 2024-03-20

**Authors:** Dobin Yim, Jiban Khuntia, Vijaya Parameswaran, Arlen Meyers

**Affiliations:** 1 Loyola University Maryland, MD United States; 2 University of Colorado Denver Denver, CO United States; 3 Stanford University Stanford, CA United States

**Keywords:** generative artificial intelligence tools and applications, GenAI, service, clinical, health care, transformation, digital

## Abstract

**Background:**

Generative artificial intelligence tools and applications (GenAI) are being increasingly used in health care. Physicians, specialists, and other providers have started primarily using GenAI as an aid or tool to gather knowledge, provide information, train, or generate suggestive dialogue between physicians and patients or between physicians and patients’ families or friends. However, unless the use of GenAI is oriented to be helpful in clinical service encounters that can improve the accuracy of diagnosis, treatment, and patient outcomes, the expected potential will not be achieved. As adoption continues, it is essential to validate the effectiveness of the infusion of GenAI as an intelligent technology in service encounters to understand the gap in actual clinical service use of GenAI.

**Objective:**

This study synthesizes preliminary evidence on how GenAI assists, guides, and automates clinical service rendering and encounters in health care The review scope was limited to articles published in peer-reviewed medical journals.

**Methods:**

We screened and selected 0.38% (161/42,459) of articles published between January 1, 2020, and May 31, 2023, identified from PubMed. We followed the protocols outlined in the PRISMA (Preferred Reporting Items for Systematic Reviews and Meta-Analyses) guidelines to select highly relevant studies with at least 1 element on clinical use, evaluation, and validation to provide evidence of GenAI use in clinical services. The articles were classified based on their relevance to clinical service functions or activities using the descriptive and analytical information presented in the articles.

**Results:**

Of 161 articles, 141 (87.6%) reported using GenAI to assist services through knowledge access, collation, and filtering. GenAI was used for disease detection (19/161, 11.8%), diagnosis (14/161, 8.7%), and screening processes (12/161, 7.5%) in the areas of radiology (17/161, 10.6%), cardiology (12/161, 7.5%), gastrointestinal medicine (4/161, 2.5%), and diabetes (6/161, 3.7%). The literature synthesis in this study suggests that GenAI is mainly used for diagnostic processes, improvement of diagnosis accuracy, and screening and diagnostic purposes using knowledge access. Although this solves the problem of knowledge access and may improve diagnostic accuracy, it is oriented toward higher value creation in health care.

**Conclusions:**

GenAI informs rather than assisting or automating clinical service functions in health care. There is potential in clinical service, but it has yet to be actualized for GenAI. More clinical service–level evidence that GenAI is used to streamline some functions or provides more automated help than only information retrieval is needed. To transform health care as purported, more studies related to GenAI applications must automate and guide human-performed services and keep up with the optimism that forward-thinking health care organizations will take advantage of GenAI.

## Introduction

### Background

Generative artificial intelligence tools and applications (GenAI) systems automatically learn patterns and structures from text, images, sounds, animation, models, or other media inputs to generate new data with similar characteristics [1]. GenAI is used to search, write, and create models, computer codes, and art forms without human assistance. GenAI has emerged significantly in the current decade to help every industry through different products such as ChatGPT, Bing Chat, Bard, LLaMA, Stable Diffusion, Midjourney, and DALL-E [2-5]. Almost all industries share an optimistic vision, with significant investment in using GenAI to transform aspects of value chains [6-10]. However, similar to many other technology hypes, whether this optimism will translate to value outcomes or be a “fad or fashion” remains to be tested over time.

The adoption of GenAI in health care is emerging. Studies point to the use of GenAI in service interactions involving breast cancer diagnoses [11], bariatric surgery [12], cardiopulmonary resuscitation [13], and breast cancer radiologic decision-making [14]. GenAI has the potential to transform by performing tasks at higher quality than humans, which may reduce errors associated with humans in expert domains such as cancer detection [15] and neurological clinical decisions [16]. The rise of GenAI is also referred to as the “second machine age” [17], whereby “instead of machines performing mechanical work they are taking on cognitive work exclusively in the human domain” [17]. Although these instances are encouraging, how exactly GenAI helps in health care processes needs to be articulated and evaluated to provide an understanding of use and value linkages [18,19]. Thus, we asked the following research questions (RQs) in this study: (1) How is GenAI used across different aspects of health care services? (RQ 1) and (2) What is the preliminary evidence of GenAI use across health care services? (RQ 2).

It is essential to explore these 2 RQs for several reasons. Exploring GenAI’s use in health care services is essential for realizing its potential benefits, addressing ethical concerns, and continually improving its applications to enhance patient care and the health care ecosystem. This impact spans different areas. For instance, GenAI can help analyze data to provide personalized treatment and tailor interventions. It has shown promise in improving diagnostic accuracy, with higher levels of accuracy in the interpretation of images and scans. AI applications can enhance patient engagement by providing personalized health recommendations, reminders for medications, and real-time monitoring of vital signs. On the provider side, GenAI can save costs by streamlining administrative tasks and improving efficiency, early disease detection, and preventive care. Similarly, knowing the preliminary evidence of GenAI use across health care services is crucial for making informed decisions, ensuring regulatory compliance, building trust, guiding research initiatives, and addressing ethical considerations. This sets the stage for the responsible and effective integration of GenAI into the health care landscape.

The impact of GenAI in health care depends on various factors, including the specific application, quality of data used for training, ethical considerations, and regulatory framework in place. Continuous monitoring, evaluation, and responsible deployment are essential to maximize the positive impact and mitigate potential negative consequences. For instance, artificial intelligence (AI) assists pathologists in diagnosing diseases from pathology slides, leading to faster and more accurate diagnoses and improving patient outcomes [20]. Analysis of oncology literature, clinical trial data, and patient records can help oncologists identify personalized, evidence-based treatment options for patients with cancer, potentially improving treatment decisions [21]. AI has been applied to analyze medical images for conditions such as diabetic retinopathy, aiding in early detection and intervention [22]. AI analyzes clinical and molecular data to help physicians make more informed decisions about cancer treatment and steer them toward personalized and effective therapies [23].

Concerns about using GenAI remain because of algorithmic bias in predictive models that causes discrimination, unequal distribution of health care resources, and exacerbated health disparities [24]. Data privacy and the need for clear guidelines on AI in health care remain a gap, with reported misuse [25]. Misinterpretations or errors in algorithms can lead to incorrect diagnoses, specifically for image readings, which underscores the importance of human oversight in critical health care decisions [26]. Furthermore, implementing and maintaining AI systems can be costly, and overreliance on technology without sufficient human oversight may result in overlooking critical clinical nuances and potentially compromising patient care [27]. Therefore, it is essential to note that the impact of AI on health care is a dynamic and evolving field. Regular updates and scrutiny of the latest research and applications are necessary to understand the positive and negative aspects of GenAI in health care.

Using a literature scoping, review, and synthesis approach in this study, we evaluated the proportionate evidence of using GenAI to assist, guide, and automate clinical service functions. Technologies in general help standardize [28], provide flexibility [29], increase experience and satisfaction through relational benefits [30], induce higher switching costs [31], and enhance the overall quality [32] and value [33] of services. However, high technology may reduce personal touch, trust, and loyalty in service settings [34-38]. Complex technologies may introduce anxiety, confusion, and isolation [39] or disconnection, disruption, and passivity stressors [13] that can erode satisfaction, loyalty, and retention in service settings [28,40-42]. Given the mixed evidence in previous research on the role of technology in services [28,43,44], it is timely to assess to what extent GenAI may even have a role in shaping or disrupting health care services. Overall, the ground realities of the potential for emerging GenAI to benefit health care services rather than just being another knowledge and collation tool need to be assessed and reported to influence further research and practice activities.

### Objectives

This study took a deep dive to review and synthesize preliminary evidence on how GenAI is used to assist, guide, and automate activities or functions during clinical service encounters in health care, with plausible indications for differential use. More evidence on the actual use is needed to assert that GenAI plays a considerable role in the digital transformation of health care. Therefore, this study aims to identify how GenAI is used in clinical settings by systematically reviewing preliminary evidence on its applications to assist, guide, and automate clinical activities or functions.

## Methods

### Article Search and Selection Strategy

**Figure 1:**
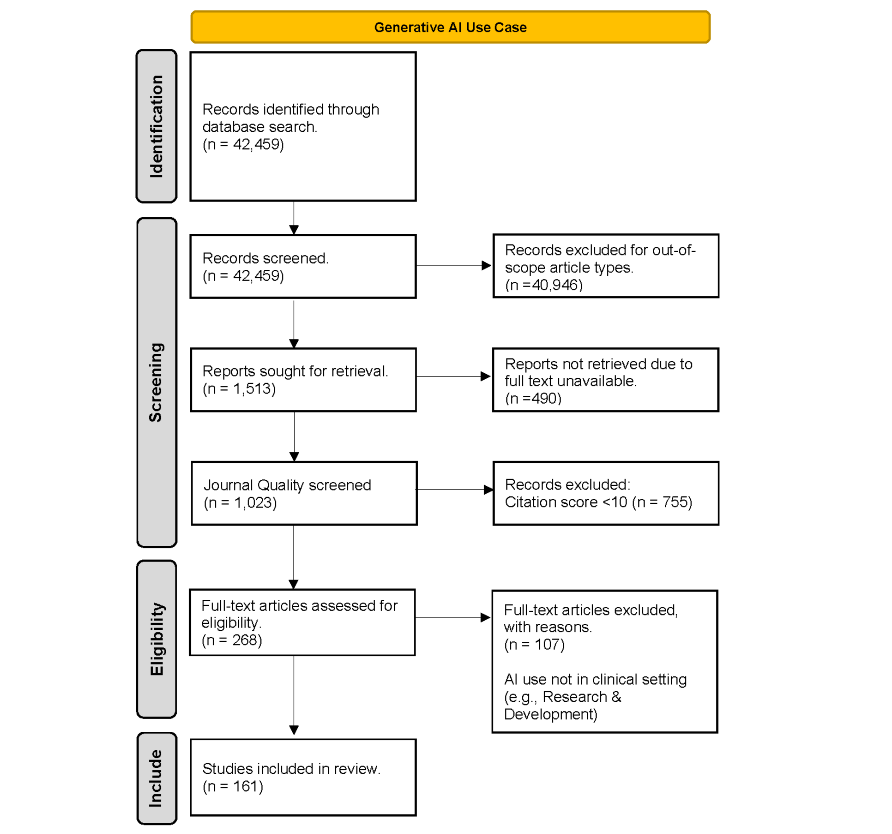
Literature screening process for relevant articles on generative artificial intelligence (AI) tools and applications.

This study aims to identify how physicians use GenAI in clinical settings, as evidenced in published studies. The design of this study adheres to the protocols outlined in the PRISMA (Preferred Reporting Items for Systematic Reviews and Meta-Analyses) statement [45,46]. Figure 1 provides a flowchart of this study’s article search and inclusion process.

We focused our search exclusively on PubMed to ensure the credibility of this study’s medical or clinical service settings. PubMed is part of the National Library of Medicine and a trusted national source of peer-reviewed publications on medical devices, software applications, and techniques used in the clinical setting. We performed keyword searches to retrieve relevant GenAI publications in PubMed that used “artificial intelligence” anywhere in the text of the article written in English. The sampling period of the publications was from January 1, 2020, to May 31, 2023. The search yielded 42,459 results in the first round of identification of articles for evaluation.

Within PubMed’s classification system for articles, we used the “article type” that described the material presented in the article (eg, review, clinical trial, retracted publication, or letter). We used this article type feature in the PubMed classification system to identify peer-reviewed articles and other relevant types of publications that are pertinent to our study. A total of 52.02% (22,086/42,459) of the returned articles did not have an article type assigned from the 75 article types in PubMed’s classification system and were excluded from the study sample. We included clinical, multicenter, case report, news, evaluation, and validation studies. We excluded article types that were out of scope, such as uncategorized articles, government-funded studies, reviews, editorials, errata, opinion articles, nonscientific articles, retracted publications, and supplementary files. We also excluded preprint article types that were unlikely to have attracted attention. Errata or retracted publications (404/42,459, 0.95%), supplementary files (117/42,459, 0.28%), and 50 article types that had too few search returns (243/42,459, 0.57%) were also excluded.

The screening stage excluded review articles (6732/42,459, 15.86%) with an objective that was neither aligned with nor redundant to this study’s goal. Opinion articles such as editorials, letters, and commentaries were excluded (2455/42,459, 5.78%). Articles whose funding came from the government or a government agency were not considered because of a conflict of interest for the researchers of the evaluated study (8936/42,459, 21.05%), and preprint articles (77/42,459, 0.2%) were excluded because of lack of availability to the public. We also considered the full text availability of the article, and 32.39% (490/1513) of the articles were excluded in the eligibility stage.

The resulting set of records included 1023 publications. To ensure the credibility of the publication source, we used CiteScore (Elsevier) [47] as a citation index to remove publication sources whose influence is limited. Any publication source whose citation index was unavailable or <10 was removed, resulting in 268 records.

In total, 2 raters, 1 author (DY) and 1 graduate assistant (BB), evaluated 161 articles. The 2 raters’ agreement was 91.93%, and the expected agreement was 82.99%. The κ score was 0.5252 (SE 0.0544; *Z* score=9.66; probability>Z score=0.0000). The author and the graduate student performed manual coding by reading the paper’s title, abstract, and introduction paragraph to gain a preliminary understanding of the study. After reading the abstract and introduction paragraph, each rater classified each article according to the definition of the 3 classes. For articles that were difficult to understand, the rater read the article further to gain a better understanding of the article. We defined clinical service settings to include the life cycle of physician encounters with patients for the diagnosis, prognosis, and management of health conditions. The research and development of drug discovery, for instance, was not considered. This process eliminated 107 records. The final data set of articles considered for this study was 161.

### Ethical Considerations

The data collected for this study were obtained from publicly available sources. The study did not involve any interaction with users. Therefore, ethics approval was not required for this study.

### Data Extraction and Categorization Process

We adopted a modified thematic synthesis approach for data analysis that involved coding the text, developing descriptive themes, and generating analytical themes [48]. Initially, each author coded each line of text extracted from the articles, assigning it to different dimensions. This line-by-line coding process facilitated identifying and capturing critical article information and concepts. Next, each author developed descriptive themes by grouping related codes and identifying common patterns or topics emerging from the coded data. These descriptive themes provided a broad overview of the various aspects of AI in the clinical service context. Building on the descriptive themes, each author generated analytical pieces to deepen the understanding and interpretation of the data. The analytical themes involved exploring relationships, connections, and implications within and across the articles, allowing for the extraction of meaningful insights.

Throughout the analysis process, all the authors engaged in extensive discussions to refine and finalize the results of the thematic synthesis. By collectively examining and interpreting the data, the research team ensured the robustness and reliability of the synthesized findings. Similar dimensions were then merged to generate the following 3 meaningful dimensions (assist, guide, and automate) and for relevance to the study objectives, as shown in Textbox 1. The researchers manually coded each article into several groups. They then tried to synthesize them into 1 of the 3 categories of *assist*, *guide*, and *automate* by looking at the title, abstract, and introduction (where applicable).

**Use of generative artificial intelligence tools and applications in clinical services in the reviewed articles (N=161).**

**Assist**

- Improve diagnostic accuracy or reduce error by accessing knowledge during clinical services (141/161, 87.6%) [49-96]
- Activities:
  Disease detection (19/161, 11.8%) [58,63,67,69,71,73,77,90,97-107]
  Diagnosis (14/161, 8.7%) [100,108-120]
  Screening (12/161, 7.5%) [65,86,87,93,121-128]
- Service areas:
  Radiology (17/161, 10.6%) [49-63,65,66]
  Cardiology (12/161, 7.5%) [67-72,74,76-79,129]
  Gastrointestinal medicine (4/161, 2.5%) [81-84]
  Diabetes (6/161, 3.7%) [86-91]
- Approaches and methods:
  Deep learning (34/161, 21.1%) [49,59,60,62,63,65,68,71,79,89,100,107,108,111,115,123,125,130-145]
  Machine learning (9/161, 5.6%) [53,55,83,91,110,146-149]
  Image analysis (13/161, 8.1%) [68,88,104,110,111,114,116,119,133,135,138,150,151]

**Guide**

- Recommend treatment options, step-by-step instructions, or checklists to improve clinical services (13/161, 8.1%) [64,80,85,96,152-160]
- Personalized treatment plans (1/161, 0.6%) [64]
- Monitoring and managing (1/161, 0.6%) [96]

**Automate**

- Minimize or eliminate human provider involvement in clinical services or follow-ups (7/161, 4.3%) [94,95,161-165]

In addition to manual coding by human researchers, we used ChatGPT (version 3.5; OpenAI) for automatic coding. ChatGPT-3.5 was used for speed and cost. ChatGPT-4 is less accessible to users who do not have the funds to pay for its monthly subscription. ChatGPT-3.5 training used one-shot learning using the standard user interface with the “foundational” mode, and no fine-tuning was performed. Future studies may use focused data sets for fine-tuning to improve classification accuracy. However, our study demonstrates that classification accuracy is high and robust even without fine-tuning. This procedure was implemented to check for any subjective bias and demonstrate AI’s potential use to complement the human coding process. The abstracts and introductions of these 161 articles were fed into ChatGPT using in-context or a few short learning processes that fine-tune a pair of domain-specific inputs and outputs to train, thereby enhancing the relevance and accuracy of ChatGPT’s automated coding output [166,167].

For instance, a sample of input we used in the study was the abstract, which summarizes the article. The output is the categories identified by the experts. ChatGPT learns how to code a set of articles by repeating the pair of inputs and outputs. One-shot learning, which consists of a single pair of inputs and outputs in general, performs as well as >2 samples and zero-shot learning. The benefits of in-context learning (ICL) in ChatGPT include enhanced relevance, where the foundational model becomes better at generating content for domain-specific tasks without additional training of the full model; controlled output such as developing a single word matching the desired coding category or variable; and reduced biases inherent in manual coding. We used the definitions provided in Textbox 1 to train and restrict ChatGPT to choose only 1 of the 3 use-case categories. We further compared ChatGPT’s classification with expert coding and found a high level of agreement between the 2, with a κ score of 0.94.

As mentioned previously, the manual coding process involved the raters coding and evaluating each article. After each rater coded the article, the results were compared and discussed to further refine the classification definition and derive consensus on the final assignment of the article classification. This “gold standard” classification was compared with automatic coding performed by ChatGPT (version 3.5). Automatic coding was performed by ChatGPT-3.5. Classification training was performed using one-shot ICL. ChatGPT learns how to classify articles by being fed a pair of articles and classification labels. For example, a user can feed a prompt or use control tokens to indicate an article abstract and the label associated with the article. In our context, 3 articles and labels were fed to the interface. After this initial prompt session of training on 3 classification labels, subsequent interactions of providing only the article abstract with a prompt asking for a class label would return ChatGPT’s prompt completion. Alternatively, training could involve >1 example of the article and its label, which would then be called *few-shot learning*. To summarize, 161 articles were coded by ChatGPT-3.5 based on a single instance of ICL.

## Results

### Findings From the Synthesis on the Use of GenAI to Assist in Different Aspects of Health Care Services

GenAI can improve clinical services in 3 ways. First, of the 161 articles, 141 (87.6%) reported using GenAI to assist services through knowledge access, collation, and filtering. The assistance of GenAI was used for disease detection (19/161, 11.8%) [58,63,67,69,71,73,77,90,97-107], diagnosis (14/161, 8.7%) [100,108-120], and screening processes (12/161, 7.5%) [65,86,87,93,121-127,168,169] in the areas of radiology (17/161, 10.6%) [49-63,65,66], cardiology (12/161, 7.5%) [67-72,74,76-79,129], gastrointestinal medicine (4/161, 2.5%) [81-84], and diabetes (6/161, 3.7%) [86-91]. Thus, although the use of GenAI has percolated across almost all disease-relevant and main service–relevant areas in health care, it is mainly for assisting through knowledge access, collation, and filtering.

The use of GenAI in disease diagnosis has long-term implications. For instance, identifying “referrable” diabetic retinopathy using routinely collected data would help in population health planning and prevention [86-90]; however, rigorous testing and validation of the applications are critical before clinical implementation [94]. Similarly, using GenAI in remote care helps improve glycemia and weight loss [95], yet challenges related to variable patient uptake and increased clinician participation necessitated by shared decision-making must be considered [96]. In radiology services, prediction models using deep learning and machine learning methods for predictive accuracy and as diagnostic aids have shown potential, and natural language processing has been used to improve readability by generating captions; however, studies report using high-quality images, highlighting the need for a future standardized pipeline for data collection and imaging detection.

In cardiology, AI analysis allows for early detection, population-level screening, and automated evaluation. It expands the reach of electrocardiography to clinical settings in which immediate interrogation of anatomy and cardiac function is needed and to locations with limited resources [67-69,71,73-75,95]. Nevertheless, there is evidence suggesting that integrating AI with patient data, including social determinants of health, enables disease prediction and early disease identification, which could lead to more precise and timely diagnoses, improving patient outcomes.

GenAI aids in diagnostic accuracy, although its focus on higher value creation in health care is limited. The articles in this review reported that they used deep learning (34/161, 21.1%) [49,59,60,62,63,65,68,71,79,89,100,107,108,111,115,123,125,130-145], machine learning (9/161, 5.6%) [53,55,83,91,110,146-149], and image analysis approaches of GenAI during the assistance process (13/161, 8.1%) [68,88,104,110,111,114,116,119,133,135,138,150,151]. Knowledge access using GenAI has the potential to enable more options and flexibility in serving patients.

### Evidence of GenAI Use for Guiding or Automation Services

Only 8.1% (13/161) of the studies provided insights into how GenAI is used to guide some services by seeking recommended treatment options, step-by-step instructions, or checklists to improve clinical services [64,80,85,96,152-160]. Of the 161 studies, 1 (0.6%) study sought personalized treatment plans and discussed monitored and managed service processes using GenAI [96]. Although this use category is nascent, GenAI can help provide speed efficiency and customized solutions in health services as in other contexts [37,127,170].

Finally, only 4.3% (7/161) of the articles indicated the use of GenAI to automate any service functions that could minimize or eliminate human provider involvement. When used appropriately, automation provides a predictable, reliable, and faster experience everywhere, every time for all customers, which will be a standardized way to provide several health care services [94,95,161-165].

The use of GenAI in some instances of service automation and guidance may be in its infancy but is encouraging. Providers are trying to explore unique ways to use AI, which requires a set of steps such as understanding the current workflow and the changes needed or aspirational workflows and aligning or designing GenAI to help in the workflow. This is similar to modifying restaurant food delivery options to suit drive-in rather than sit-in options. The providers need some work to fully automate, streamline, or re-engineer the service functions using GenAI in the future.

### Summary of Findings

To summarize our findings, in this study, we conducted a systematic scoping review of the literature on how GenAI is used in clinical settings by synthesizing evidence on its application to assist, guide, and automate clinical activities and functions. Of the 161 articles, 141 (87.6%) reported using GenAI to assist services through knowledge access, collation, and filtering. The assistance of GenAI was used for disease detection (19/161, 11.8%), diagnosis (14/161, 8.7%), and screening processes (12/161, 7.5%) in the areas of radiology (17/161, 10.6%), cardiology (12/161, 7.5%), gastrointestinal medicine (4/161, 2.5%), and diabetes (6/161, 3.7%). Thus, we conclude that GenAI mainly informs rather than assisting and automating service functions. Presumably, the potential in clinical service is there, but it has yet to be actualized for GenAI.

### Robustness Check Using Additional Database Search

To ensure the comprehensiveness and robustness of our findings, we expanded the search to Web of Science using similar keywords and strategies (suggested by the review team). We used the same keyword, “artificial intelligence,” in all text fields over the sampling period between January 1, 2020, and November 27, 2023. Our search was restricted to peer-reviewed academic journal articles written in English. We used the Web of Science–provided “Highly Cited Papers” criterion as a filtering mechanism to follow influential papers. Given the nonclinical context of the journals in the database, we believe that filtering based on the article’s importance is reasonable. Initial search results included 1958 articles from the Web of Science Core Collection. The preliminary analysis of the annual breakdown comprised 414 articles in 2023, a total of 651 articles in 2022, a total of 519 articles in 2021, and a total of 374 articles in 2020. The search results were further reduced by removing PubMed articles for redundancy, resulting in 1221 articles.

Next, Web of Science journals include medical, nonmedical, and other clinical journals. Thus, we used simple keywords for filtering nonmedical and clinical contexts. We used the keywords “medical” and “health” mentioned in the abstract, which led to 133 articles. Finally, we read the abstracts and titles to exclude survey or meta-review and nonclinical studies. This process further narrowed down the selection to 51 relevant articles. Using ChatGPT-3.5 on November 27, 2023, we applied one-shot learning by providing 3 class definitions. We asked ChatGPT-3.5 to classify the article’s abstract, with 63% (32/51) in the *assist* category, 29% (15/51) in the *guide* category, and 8% (4/51) in the *automated* category. Diagnostic assistance articles dominated, similar to the results from PubMed. However, the other categories—prescriptive guidance and clinical service recommendations—were slightly higher. This difference is explained by the nonmedical and clinical nature of the journals included in the database. The “applied” nature of the journals is more likely to explore prescriptive guidance and clinical service recommendation use cases.

## Discussion

### Principal Findings

This study asked RQs about how GenAI is used, with evidence, to shape health care services. It showed that 11.8% (19/161) of the studies were on automation and guidance, whereas 87.6% (141/161) reflected the assistance role of GenAI. These findings are essential to discuss and distinguish between the optimism and actual use of GenAI in health care.

### Study Implications

The aspiration that GenAI has the potential to change health care significantly needs a careful revisit. Health care organizations need to assess the actual ground use for GenAI and prepare for and understand the exciting possibilities with a cautious approach rather than overly high expectations. Concerns related to the cost, privacy, misuse, and regulatory aspects of implementing and using GenAI [24-26] will become more pronounced, particularly when there is a perceived overreliance without clear promising results or actual practical use [26].

The literature synthesis in this study suggests that GenAI is mainly used for screening and diagnostic purposes using knowledge access; diagnostic processes such as predicted disease outcomes, survival, or disease classification; and improvement of the accuracy of diagnosis. This solves the problem of knowledge being available and accessible in time in a well-articulated manner to provide or render the services. This could help health care providers make more accurate and timely diagnoses, leading to earlier treatment and better patient outcomes. Such knowledge distillation helps improve diagnostic accuracy through GenAI, which can provide enough knowledge to physicians during service encounters; however, this is not hugely oriented toward higher value creation in health care.

The research synthesis also suggests that there has been some use of GenAI during different steps and aspects of guiding the service delivery processes. Still, such use could be more encouraging and significant across the board. Plausibly, GenAI can analyze large amounts of disparate data from patients to suggest personalized medicine—which may help inform treatment plans for individuals. Service delivery needs some guidance or step-by-step help to be efficient and meet the duration or time requirements to render the clinical service on time, which GenAI may solve. However, we have not yet found strong evidence for such use by any health system.

Currently, the automation of service functions using GenAI has only seen minimal instances and is yet to see widespread implementation. Automation helps offset some manual activities. However, automation may help in service functions’ cost, efficiency, and flexibility while maintaining some standards across similar services.

Similarly, although we did not consider this area in the synthesis as it was out of the scope of services, GenAI can also be used in drug development and clinical trial pathways—a value proposition yet to be seen in practice. However, we do not undermine that many laboratories and pharmaceutical companies have used machine learning and AI tools and techniques in drug development and clinical trials. However, reported commercial GenAI use has not come to the limelight.

Some other plausible uses of GenAI in health care include managing supply chain data, managing medical equipment assets, maintaining gadgets and equipment, and building a robust intelligent information infrastructure to support several other activities. For example, active efforts are being undertaken to incorporate GenAI, especially in administrative use cases such as the In Basket patient messaging applications. However, assessing the clinical accuracy of such tools remains a concern.

In addition, we must incorporate user-centered design and sociotechnical frameworks into designing and building GenAI for health care use cases, for instance, to explore how GenAI can prevent a common pitfall of developing models opportunistically—based on data availability or end-point labels, adopting a user-centered design framework is vital for GenAI tools [171]. Similarly, scientific or research-oriented use of GenAI for knowledge search, articulation, or synthesis is helpful [172]. However, how far that will translate to the transformative clinical health care delivery processes while creating higher-order organizational capabilities to create value remains a concern [173].

### Limitations of the Study and Scope for Future Research

Several limitations and constraints affect the interpretation and generalizability of the findings of this study. Some of these limitations indicate the need for future research in relevant areas that we discuss further. First, the study’s findings were constrained by the availability of relevant and high-quality publications and the exclusion of preprints and unpublished data to limit the specifically designed scope of the study on using GenAI in health care clinical services, which influences the comprehensiveness and accuracy of the review. There also might be a tendency for studies with positive or significant results to be published, leading to a potential publication bias. In addition, harmful or neutral findings may not be adequately represented in the review, influencing the overall assessment of GenAI's effectiveness in health care. Research should focus on patient-centered outcomes, including patient satisfaction and engagement and the impact of GenAI on the patient-provider relationship. Understanding the patient perspective is crucial for successfully integrating AI technologies into health care.

Second, the field of GenAI in health care is rapidly advancing, and new technologies and applications are continuously emerging. The findings of this study might not capture the most recent developments, and the ’conclusions of this study may become outdated quickly, specifically when some technologies have the potential to be adopted beyond institutional mechanisms, such as using GenAI mobile apps to scan images for retinopathy. Furthermore, an in-depth analysis of specific GenAI applications may open newer directions, and future research should focus on specific GenAI applications to provide detailed insights into their effectiveness and limitations. This could include applications such as diagnostic tools, treatment planning algorithms, and predictive analytics. Such heterogeneity of GenAI in health care encompasses a wide range of applications, and investigating these could make it challenging to draw overarching conclusions about GenAI’s impact on clinical services.

Third, this review may not comprehensively address ethical considerations and potential biases in the use of GenAI in health care. Ethical issues related to data privacy, algorithmic bias, and the responsible deployment of AI technologies may require more in-depth exploration. Future research should systematically explore the ethical considerations associated with GenAI use in health care. This includes issues related to data privacy, consent, transparency, and the ethical deployment of AI algorithms in clinical settings. Finally, more data, papers, articles, and longitudinal developments on some applications may enrich this study and enhance its current limited generalizability. Longitudinal studies are needed to track the impact of GenAI in health care over an extended period. This will help researchers understand the sustained effects, identify potential challenges that may arise over time, and assess the scalability and adaptability of these technologies.

Future studies could undertake comparative effectiveness research to assess how GenAI compares with traditional approaches in health care. Understanding the relative advantages and disadvantages will contribute to evidence-based decision-making. In addition, it is not clear what and how to measure the GenAI applications’ effectiveness in clinical services, leading to a call for standardized study metrics that can incorporate outcome measures and evaluation frameworks. Future research should investigate how the integration of GenAI into clinical health care services affects the workflow of health care providers. This includes understanding the time savings, challenges, and potential improvements in decision-making processes. By addressing these areas, future research can contribute to a more comprehensive understanding of the role, challenges, and potential benefits of GenAI in clinical health care services.

### Actionable Policy and Practice Recommendations

The proliferation of technology often outpaces the development of appropriate regulatory and policy frameworks that are necessary for guiding proper dissemination. Our call is that, given that GenAI is emerging, policy agencies and health care organizations play a role in proactively guiding the use of GenAI in health care organizations.

What are some actionable steps for stakeholders, including health care organizations and policy makers, to navigate the integration of GenAI in health care? For health care organizations, the steps may include conducting a technology assessment vis-à-vis goals to achieve outcomes from GenAI. Evaluating the existing infrastructure and technological capabilities within the health care organization to determine readiness for GenAI integration is a first step. This will provide an understanding of the current state of technology and ensure that the necessary upgrades or modifications can be implemented to support GenAI applications, thus garnering the benefits of GenAI.

The second step is to invest in staff training and education through the development of training programs to enhance the skills of health care professionals in understanding and using GenAI technologies. Well-trained staff is essential for the effective and ethical implementation of GenAI, fostering a culture of continuous learning and adaptability. Third, health care organizations need to develop and communicate clear protocols and guidelines for the use of GenAI in different health care services, outlining ethical considerations, data privacy measures, and accountability standards. Transparent protocols help ensure the responsible and standardized use of GenAI, fostering trust among health care professionals and patients.

Fourth, health care organizations need to engage in research on GenAI through collaboration with research institutions and industry partners to participate actively in studies evaluating the effectiveness and impact of GenAI applications in specific health care domains. Involvement in research contributes to the evidence base, informs best practices, and positions the organization as a leader in health care innovation. Finally, as mentioned previously, implementing the gradual integration of GenAI rather than jumping into irrational decisions is a caution. All health systems need to gradually plan and introduce GenAI technologies, starting with pilot programs in specific departments or use cases. Gradual integration allows for careful monitoring of performance, identification of potential challenges, and iterative improvement before broader implementation.

For policy makers, much work must be done at the regulatory framework level to realize GenAI better. Policy makers must establish clear and adaptive regulatory frameworks that address the unique challenges GenAI poses in health care, ensuring patient safety, data privacy, and ethical use. There is a concern that bias in GenAI algorithms could lead to discrimination in care delivery across patients, and the role of policy guidelines in this aspect to train and use GenAI appropriately is critical. Policy frameworks must be developed to ensure less risk, safe and ethical use, and responsible effectiveness of GenAI. Policy and industry partnerships among experts to determine relevant frameworks are vital to guide the future of GenAI to help transform health care. Robust regulations will provide a foundation for the responsible and standardized integration of GenAI technologies. An underlying challenge of GenAI is integrating it across different legacy IT systems, which involves developing and adopting interoperability standards to ensure seamless communication and data exchange between different GenAI applications and existing health care systems. Interoperability enhances efficiency, reduces redundancy, and facilitates the integration of diverse GenAI solutions. In this process, creating incentives for responsible innovation for ethical considerations and the continuous improvement of GenAI applications will drive a culture of responsibility and quality improvement, aligning technological advancements with societal needs.

Policy-level efforts also need to be oriented to allocate resources to enhance health care infrastructure, including robust connectivity and data storage capabilities, to support the data-intensive nature of GenAI applications. Adequate infrastructure is crucial for the reliable and secure functioning of GenAI in health care. Many of these enhancements may require collaboration between public health care systems, private organizations, and academia to leverage collective expertise and resources for GenAI research, development, and implementation. Finally, policies that address potential biases in GenAI applications and ensure equitable access to these technologies across diverse populations are necessary to help with proactive measures to prevent the exacerbation of existing health care disparities through the adoption of GenAI.

### Conclusions

GenAI is both a tool and a complex technology. Complexity is the basis for GenAI, and thus, the use of GenAI in health care creates a set of unparalleled challenges. GenAI is costly to implement and integrate across all aspects of a health system [174]. In envisioning the future of GenAI in health care, we glimpse a transformative landscape in which technology and compassion converge for the betterment of humanity. As we stand at the intersection of innovation and responsibility, the prospect of GenAI holds immense promise in revolutionizing health care, shaping a future in which personalized, efficient, and equitable clinical services are not just aspirations but tangible realities. Our vision embraces a symbiotic relationship between technology and human touch, recognizing that the power of GenAI lies not only in its computational prowess but also in its potential to amplify the capabilities of health care professionals. Picture a world in which diagnostic accuracy is elevated, treatment plans are truly personalized, and each patient’s journey is marked by precision and empathy.

Crucially, this vision hinges on responsible adoption. We envisage a future in which regulatory frameworks ensure the ethical use of GenAI, safeguard patient privacy, and uphold the principles of equity. It is a future in which interdisciplinary collaboration flourishes, bridging the expertise of health care providers, policy makers, technologists, and ethicists to navigate the complexities of this evolving landscape.

In the future, the impact of AI on human lives will be profound. Patients experience a health care system that not only heals but also understands, a system in which the integration of GenAI contributes to quicker diagnoses, more effective treatments, and improved outcomes. The human experience is at the forefront—GenAI becomes a tool for health care professionals to better connect with patients and spend more time understanding their unique needs, fears, and hopes. As we embark on this journey, it is crucial to remember that the heart of health care lies in the compassion, empathy, and wisdom of its human stewards. GenAI catalyzes empowerment, freeing health care professionals from mundane tasks to engage in meaningful interactions. It fosters a health care culture in which technology serves humanity, and the collective mission is to enhance the quality of care and life.

In embracing this vision, we are not just architects of technological progress but also custodians of a future in which GenAI and human touch coalesce to redefine health care possibilities. Let our strides be guided by a commitment to responsible innovation, a dedication to inclusivity, and an unwavering focus on the well-being of those we serve. The future of GenAI in health care is not just a scientific evolution, but it is a narrative of healing; compassion; and a shared commitment to a healthier, more humane world. However, without enough evidence, we are skeptical about the current euphoria regarding GenAI in health care.

This systematic narrative review of the preliminary evidence of using GenAI in health care clinical services provides valuable insights into the evolving landscape of AI applications in health care. The existing literature synthesis reveals promising advancements and critical considerations for integrating GenAI into clinical settings. The positive evidence underscores the potential of GenAI to revolutionize health care by offering personalized treatment plans, enhancing diagnostic accuracy, and contributing to the development of innovative therapeutic solutions. The applications of GenAI in areas such as pathology assistance, oncology decision support, and medical imaging interpretation showcase its capacity to augment health care professionals’ capabilities and improve patient outcomes.

However, this review also highlights several limitations and challenges that warrant careful consideration. Issues such as the quality of available data, the rapid pace of technological evolution, and the potential for algorithmic bias highlight the complexities associated with adopting GenAI in health care. Ethical concerns, data privacy considerations, and the need for transparent guidelines underscore the importance of a thoughtful and measured approach to integration.

As we navigate the preliminary evidence, it becomes evident that a collaborative effort is required among health care organizations, policy makers, researchers, and technology developers. Establishing clear regulatory frameworks, fostering interdisciplinary collaboration, and prioritizing ethical considerations are crucial steps in ensuring the responsible deployment of GenAI. Addressing the identified limitations through targeted research initiatives, ongoing evaluation, and continuous improvement will be essential for maximizing the benefits of GenAI while mitigating potential risks.

Moving forward, it is imperative to recognize that integrating GenAI into health care is dynamic and evolving. Future research should focus on refining our understanding of the long-term impact, patient-centered outcomes, and scalability of GenAI applications. By collectively addressing the challenges outlined in this review, stakeholders can contribute to a health care landscape in which GenAI is a powerful ally in delivering personalized, efficient, and equitable clinical services.

## Appendix

Multimedia Appendix 1 PRISMA checklist.

Multimedia Appendix 2 Conversations with ChatGPT used in the Study.

## Abbreviations

AI: artificial intelligence
GenAI: generative artificial intelligence tools and applications
ICL: in-context learning
PRISMA: Preferred Reporting Items for Systematic Reviews and Meta-Analyses
RQ: research question

## References

[1] Pasick A (2023). Artificial intelligence glossary: neural networks and other terms explained. The New York Times.

[2] Roose K (2022). A coming-out party for generative A.I., Silicon Valley’s new craze. The New York Times.

[3] Karpathy A, Abeel P, Brockman G, Chen P, Cheung V, Duan Y (2016). Generative models. Open AI.

[4] Metz C (2023). OpenAI plans to up the ante in tech’s A.I. race. The New York Times.

[5] Thoppilan R, De Freitas D, Hall J, Shazeer N, Kulshreshtha A, Cheng HT, Jin A, Bis T, Baker L, Du Y, Li Y, Lee H, Zheng HS, Ghafouri A, Menegali M, Huang Y (2020). LaMDA: language models for dialog applications. arXiv Preprint posted online January 20, 2022.

[6] (2023). Don’t fear an ai-induced jobs apocalypse just yet: the west suffers from too little automation, not too much. The Economist.

[7] Harreis H, Koullias T, Roberts R, Te K Generative AI: unlocking the future of fashion. McKinsey & Company.

[8] Eapen TT, Venkataswamy L, Finkenstadt DJ, Folk J (2023). How generative AI can augment human creativity. Harvard Business Review.

[9] (2023). The race of the AI labs heats up: ChatGPT is not the only game in town. The Economist.

[10] (2023). Google Cloud brings generative AI to developers, businesses, and governments. Google.

[11] Zheng D, He X, Jing J (2023). Overview of artificial intelligence in breast cancer medical imaging. J Clin Med.

[12] Samaan JS, Yeo YH, Rajeev N, Hawley L, Abel S, Ng WH, Srinivasan N, Park J, Burch M, Watson R, Liran O, Samakar K (2023). Assessing the accuracy of responses by the language model ChatGPT to questions regarding bariatric surgery. Obes Surg.

[13] Ahn C (2023). Exploring ChatGPT for information of cardiopulmonary resuscitation. Resuscitation.

[14] Rao A, Kim J, Kamineni M, Pang M, Lie W, Succi MD (2023). Evaluating ChatGPT as an adjunct for radiologic decision-making. medRxiv. Preprint posted online February 7, 2023.

[15] Cirillo D, Núñez-Carpintero I, Valencia A (2021). Artificial intelligence in cancer research: learning at different levels of data granularity. Mol Oncol.

[16] Pedersen M, Verspoor K, Jenkinson M, Law M, Abbott DF, Jackson GD (2020). Artificial intelligence for clinical decision support in neurology. Brain Commun.

[17] Brynjolfsson E, McAfee A (2014). Second Machine Age: Work, Progress, and Prosperity in a Time of Brilliant Technologies.

[18] Raisch S, Krakowski S (2021). Artificial intelligence and management: the automation–augmentation paradox. Acad Manage Rev.

[19] Haug CJ, Drazen JM (2023). Artificial intelligence and machine learning in clinical medicine, 2023. N Engl J Med.

[20] Cascella M, Montomoli J, Bellini V, Bignami E (2023). Evaluating the feasibility of ChatGPT in healthcare: an analysis of multiple clinical and research scenarios. J Med Syst.

[21] Baxi V, Edwards R, Montalto M, Saha S (2022). Digital pathology and artificial intelligence in translational medicine and clinical practice. Mod Pathol.

[22] Yu SH, Kim MS, Chung HS, Hwang EC, Jung SI, Kang TW, Kwon D (2021). Early experience with Watson for Oncology: a clinical decision-support system for prostate cancer treatment recommendations. World J Urol.

[23] Wang Z, Keane PA, Chiang M, Cheung CY, Wong TY, Ting DS, Lidströmer N, Ashrafian H (2022). Artificial intelligence and deep learning in ophthalmology. Artificial Intelligence in Medicine.

[24] Osinski B, BenTaieb A, Ho I, Jones RD, Joshi RP, Westley A, Carlson M, Willis C, Schleicher L, Mahon BM, Stumpe MC (2022). Artificial intelligence-augmented histopathologic review using image analysis to optimize DNA yield from formalin-fixed paraffin-embedded slides. Mod Pathol.

[25] Obermeyer Z, Powers B, Vogeli C, Mullainathan S (2019). Dissecting racial bias in an algorithm used to manage the health of populations. Science.

[26] Jones C, Thornton J, Wyatt JC (2023). Artificial intelligence and clinical decision support: clinicians' perspectives on trust, trustworthiness, and liability. Med Law Rev.

[27] Degnan AJ, Ghobadi EH, Hardy P, Krupinski E, Scali EP, Stratchko L, Ulano A, Walker E, Wasnik AP, Auffermann WF (2019). Perceptual and interpretive error in diagnostic radiology-causes and potential solutions. Acad Radiol.

[28] Khanna NN, Maindarkar MA, Viswanathan V, Fernandes JF, Paul S, Bhagawati M, Ahluwalia P, Ruzsa Z, Sharma A, Kolluri R, Singh IM, Laird JR, Fatemi M, Alizad A, Saba L, Agarwal V, Sharma A, Teji JS, Al-Maini M, Rathore V, Naidu S, Liblik K, Johri AM, Turk M, Mohanty L, Sobel DW, Miner M, Viskovic K, Tsoulfas G, Protogerou AD, Kitas GD, Fouda MM, Chaturvedi S, Kalra MK, Suri JS (2022). Economics of artificial intelligence in healthcare: diagnosis vs. treatment. Healthcare (Basel).

[29] Curran JM, Meuter ML (2005). Self-service technology adoption: comparing three technologies. J Serv Mark.

[30] Choudhury V, Karahanna E (2008). The relative advantage of electronic channels: a multidimensional view. MIS Q.

[31] Marzocchi GL, Zammit A (2006). Self-scanning technologies in retail: determinants of adoption. Serv Ind J.

[32] Campbell D, Frei F (2010). Cost structure, customer profitability, and retention implications of self-service distribution channels: evidence from customer behavior in an online banking channel. Manag Sci.

[33] Chen PY, Hitt LM (2002). Measuring switching costs and the determinants of customer retention in internet-enabled businesses: a study of the online brokerage industry. Inf Syst Res.

[34] Mols NP (1998). The behavioral consequences of PC banking. Int J Bank Mark.

[35] Apte UM, Vepsäläinen AP (1993). High tech or high touch? Efficient channel strategies for delivering financial services. J Strateg Inf Syst.

[36] Giebelhausen M, Robinson SG, Sirianni NJ, Brady MK (2014). Touch versus tech: when technology functions as a barrier or a benefit to service encounters. J Mark.

[37] Selnes F, Hansen H (2016). The potential hazard of self-service in developing customer loyalty. J Serv Res.

[38] Walker RH, Johnson LW (2006). Why consumers use and do not use technology-enabled services. J Serv Mark.

[39] Xue M, Hitt LM, Harker PT (2007). Customer efficiency, channel usage, and firm performance in retail banking. Manuf Serv Oper Manag.

[40] Johnson DS, Bardhi F, Dunn DT (2008). Understanding how technology paradoxes affect customer satisfaction with self‐service technology: the role of performance ambiguity and trust in technology. Psychol Mark.

[41] Scherer A, Wünderlich NV, von Wangenheim F (2015). The value of self-service: long-term effects of technology-based self-service usage on customer retention. MIS Q.

[42] Li S, Sun B, Wilcox RT (2018). Cross-selling sequentially ordered products: an application to consumer banking services. J Mark Res.

[43] Bitner MJ, Brown SW, Meuter ML (2000). Technology infusion in service encounters. J Acad Mark Sci.

[44] Meuter ML, Ostrom AL, Roundtree RI, Bitner MJ (2018). Self-service technologies: understanding customer satisfaction with technology-based service encounters. Journal of Marketing.

[45] Page MJ, Moher D, Bossuyt PM, Boutron I, Hoffmann TC, Mulrow CD (2021). PRISMA 2020 explanation and elaboration: updated guidance and exemplars for reporting systematic reviews. BMJ.

[46] Bitner M (2001). Service and technology: opportunities and paradoxes. Manag Serv Qual.

[47] Page MJ, McKenzie JA, Bossuyt PM, Boutron I, Hoffmann TC, Mulrow CD, Shamseer L, Tetzlaff JM, Akl EA, Brennan SE, Chou R, Glanville J, Grimshaw JM, Hróbjartsson A, Lalu MM, Li T, Loder EW, Mayo-Wilson E, McDonald S, McGuinness LA, Stewart LA, Thomas J, Tricco AC, Welch VA, Whiting P, Moher D (2021). The PRISMA 2020 statement: an updated guideline for reporting systematic reviews. Int J Surg.

[48] Baker DW (2020). Introducing CiteScore, our journal's preferred citation index: moving beyond the impact factor. Jt Comm J Qual Patient Saf.

[49] Wang Y, Yao Q, Kwok JT, Ni LM (2020). Generalizing from a few examples: a survey on few-shot learning. ACM Comput Surv.

[50] Dong D, Fang MJ, Tang L, Shan XH, Gao JB, Giganti F, Wang R, Chen X, Wang X, Palumbo D, Fu J, Li W, Li J, Zhong L, De Cobelli F, Ji J, Liu Z, Tian J (2020). Deep learning radiomic nomogram can predict the number of lymph node metastasis in locally advanced gastric cancer: an international multicenter study. Ann Oncol.

[51] Ruscitti P, Bruno F, Berardicurti O, Acanfora C, Pavlych V, Palumbo P, Conforti A, Carubbi F, Di Cola I, Di Benedetto P, Cipriani P, Grassi D, Masciocchi C, Iagnocco A, Barile A, Giacomelli R (2020). Lung involvement in macrophage activation syndrome and severe COVID-19: results from a cross-sectional study to assess clinical, laboratory and artificial intelligence-radiological differences. Ann Rheum Dis.

[52] Shao L, Yan Y, Liu Z, Ye X, Xia H, Zhu X, Zhang Y, Zhang Z, Chen H, He W, Liu C, Lu M, Huang Y, Ma L, Sun K, Zhou X, Yang G, Lu J, Tian J (2020). Radiologist-like artificial intelligence for grade group prediction of radical prostatectomy for reducing upgrading and downgrading from biopsy. Theranostics.

[53] Liu X, Zhang D, Liu Z, Li Z, Xie P, Sun K, Wei W, Dai W, Tang Z, Ding Y, Cai G, Tong T, Meng X, Tian J (2021). Deep learning radiomics-based prediction of distant metastasis in patients with locally advanced rectal cancer after neoadjuvant chemoradiotherapy: a multicentre study. EBioMedicine.

[54] Gitto S, Cuocolo R, Annovazzi A, Anelli V, Acquasanta M, Cincotta A, Albano D, Chianca V, Ferraresi V, Messina C, Zoccali C, Armiraglio E, Parafioriti A, Sciuto R, Luzzati A, Biagini R, Imbriaco M, Sconfienza LM (2021). CT radiomics-based machine learning classification of atypical cartilaginous tumours and appendicular chondrosarcomas. EBioMedicine.

[55] Zhang J, Yao K, Liu P, Liu Z, Han T, Zhao Z, Cao Y, Zhang G, Zhang J, Tian J, Zhou J (2020). A radiomics model for preoperative prediction of brain invasion in meningioma non-invasively based on MRI: a multicentre study. EBioMedicine.

[56] Hindocha S, Charlton TG, Linton-Reid K, Hunter B, Chan C, Ahmed M, Robinson EJ, Orton M, Ahmad S, McDonald F, Locke I, Power D, Blackledge M, Lee RW, Aboagye EO (2022). A comparison of machine learning methods for predicting recurrence and death after curative-intent radiotherapy for non-small cell lung cancer: development and validation of multivariable clinical prediction models. EBioMedicine.

[57] Feng L, Liu Z, Li C, Li Z, Lou X, Shao L, Wang Y, Huang Y, Chen H, Pang X, Liu S, He F, Zheng J, Meng X, Xie P, Yang G, Ding Y, Wei M, Yun J, Hung M, Zhou W, Wahl DR, Lan P, Tian J, Wan X (2022). Development and validation of a radiopathomics model to predict pathological complete response to neoadjuvant chemoradiotherapy in locally advanced rectal cancer: a multicentre observational study. Lancet Digit Health.

[58] Seah JC, Tang CH, Buchlak QD, Holt XG, Wardman JB, Aimoldin A, Esmaili N, Ahmad H, Pham H, Lambert JF, Hachey B, Hogg SJF, Johnston BP, Bennett C, Oakden-Rayner L, Brotchie P, Jones CM (2021). Effect of a comprehensive deep-learning model on the accuracy of chest x-ray interpretation by radiologists: a retrospective, multireader multicase study. Lancet Digit Health.

[59] Fontanellaz M, Ebner L, Huber A, Peters A, Löbelenz L, Hourscht C, Klaus J, Munz J, Ruder T, Drakopoulos D, Sieron D, Primetis E, Heverhagen JT, Mougiakakou S, Christe A (2021). A deep-learning diagnostic support system for the detection of COVID-19 using chest radiographs: a multireader validation study. Invest Radiol.

[60] Gu J, Tong T, Xu D, Cheng F, Fang C, He C, Wang J, Wang B, Yang X, Wang K, Tian J, Jiang T (2023). Deep learning radiomics of ultrasonography for comprehensively predicting tumor and axillary lymph node status after neoadjuvant chemotherapy in breast cancer patients: A multicenter study. Cancer.

[61] Jiang M, Li CL, Luo XM, Chuan ZR, Lv WZ, Li X, Cui X, Dietrich CF (2021). Ultrasound-based deep learning radiomics in the assessment of pathological complete response to neoadjuvant chemotherapy in locally advanced breast cancer. Eur J Cancer.

[62] Zhang Y, Liu M, Zhang L, Wang L, Zhao K, Hu S, Chen X, Xie X (2023). Comparison of chest radiograph captions based on natural language processing vs completed by radiologists. JAMA Netw Open.

[63] Yoon AP, Lee YL, Kane RL, Kuo C, Lin C, Chung KC (2021). Development and validation of a deep learning model using convolutional neural networks to identify scaphoid fractures in radiographs. JAMA Netw Open.

[64] Yoo H, Kim KH, Singh R, Digumarthy SR, Kalra MK (2020). Validation of a deep learning algorithm for the detection of malignant pulmonary nodules in chest radiographs. JAMA Netw Open.

[65] Zhong L, Dong D, Fang X, Zhang F, Zhang N, Zhang L, Fang M, Jiang W, Liang S, Li C, Liu Y, Zhao X, Cao R, Shan H, Hu Z, Ma J, Tang L, Tian J (2021). A deep learning-based radiomic nomogram for prognosis and treatment decision in advanced nasopharyngeal carcinoma: a multicentre study. EBioMedicine.

[66] Lu MT, Raghu VK, Mayrhofer T, Aerts HJ, Hoffmann U (2020). Deep learning using chest radiographs to identify high-risk smokers for lung cancer screening computed tomography: development and validation of a prediction model. Ann Intern Med.

[67] Ahn JS, Ebrahimian S, McDermott S, Lee S, Naccarato L, Di Capua JF, Wu MY, Zhang EW, Muse V, Miller B, Sabzalipour F, Bizzo BC, Dreyer KJ, Kaviani P, Digumarthy SR, Kalra MK (2022). Association of artificial intelligence-aided chest radiograph interpretation with reader performance and efficiency. JAMA Netw Open.

[68] Upton R, Mumith A, Beqiri A, Parker A, Hawkes W, Gao S, Porumb M, Sarwar R, Marques P, Markham D, Kenworthy J, O'Driscoll JM, Hassanali N, Groves K, Dockerill C, Woodward W, Alsharqi M, McCourt A, Wilkes EH, Heitner SB, Yadava M, Stojanovski D, Lamata P, Woodward G, Leeson P (2022). Automated echocardiographic detection of severe coronary artery disease using artificial intelligence. JACC Cardiovasc Imaging.

[69] Kusunose K, Abe T, Haga A, Fukuda D, Yamada H, Harada M, Sata M (2020). A deep learning approach for assessment of regional wall motion abnormality from echocardiographic images. JACC Cardiovasc Imaging.

[70] Ko WY, Siontis KC, Attia ZI, Carter RE, Kapa S, Ommen SR, Demuth SJ, Ackerman MJ, Gersh BJ, Arruda-Olson AM, Geske JB, Asirvatham SJ, Lopez-Jimenez F, Nishimura RA, Friedman PA, Noseworthy PA (2020). Detection of hypertrophic cardiomyopathy using a convolutional neural network-enabled electrocardiogram. J Am Coll Cardiol.

[71] Vaid A, Johnson KW, Badgeley MA, Somani SS, Bicak M, Landi I, Russak A, Zhao S, Levin MA, Freeman RS, Charney AW, Kukar A, Kim B, Danilov T, Lerakis S, Argulian E, Narula J, Nadkarni GN, Glicksberg BS (2022). Using deep-learning algorithms to simultaneously identify right and left ventricular dysfunction from the electrocardiogram. JACC Cardiovasc Imaging.

[72] Elias P, Poterucha TJ, Rajaram V, Moller LM, Rodriguez V, Bhave S, Hahn RT, Tison G, Abreau SA, Barrios J, Torres JN, Hughes JW, Perez MV, Finer J, Kodali S, Khalique O, Hamid N, Schwartz A, Homma S, Kumaraiah D, Cohen DJ, Maurer MS, Einstein AJ, Nazif T, Leon MB, Perotte AJ (2022). Deep learning electrocardiographic analysis for detection of left-sided valvular heart disease. J Am Coll Cardiol.

[73] Yao X, Rushlow DR, Inselman JW, McCoy RG, Thacher TD, Behnken EM, Bernard ME, Rosas SL, Akfaly A, Misra A, Molling PE, Krien JS, Foss RM, Barry BA, Siontis KC, Kapa S, Pellikka PA, Lopez-Jimenez F, Attia ZI, Shah ND, Friedman PA, Noseworthy PA (2021). Artificial intelligence-enabled electrocardiograms for identification of patients with low ejection fraction: a pragmatic, randomized clinical trial. Nat Med.

[74] Wu S, Chen X, Pan J, Dong W, Diao X, Zhang R, Zhang Y, Zhang Y, Qian G, Chen H, Lin H, Xu S, Chen Z, Zhou X, Mei H, Wu C, Lv Q, Yuan B, Chen Z, Liao W, Yang X, Chen H, Huang J, Lin T (2022). An artificial intelligence system for the detection of bladder cancer via cystoscopy: a multicenter diagnostic study. J Natl Cancer Inst.

[75] Narang A, Bae R, Hong H, Thomas Y, Surette S, Cadieu C, Chaudhry A, Martin RP, McCarthy PM, Rubenson DS, Goldstein S, Little SH, Lang RM, Weissman NJ, Thomas JD (2021). Utility of a deep-learning algorithm to guide novices to acquire echocardiograms for limited diagnostic use. JAMA Cardiol.

[76] Yuan XL, Guo LJ, Liu W, Zeng X, Mou Y, Bai S, Pan ZG, Zhang T, Pu WF, Wen C, Wang J, Zhou ZD, Feng J, Hu B (2022). Artificial intelligence for detecting superficial esophageal squamous cell carcinoma under multiple endoscopic imaging modalities: a multicenter study. J Gastroenterol Hepatol.

[77] Attia ZI, Kapa S, Dugan J, Pereira N, Noseworthy PA, Jimenez FL, Cruz J, Carter RE, DeSimone DC, Signorino J, Halamka J, Chennaiah Gari NR, Madathala RS, Platonov PG, Gul F, Janssens SP, Narayan S, Upadhyay GA, Alenghat FJ, Lahiri MK, Dujardin K, Hermel M, Dominic P, Turk-Adawi K, Asaad N, Svensson A, Fernandez-Aviles F, Esakof DD, Bartunek J, Noheria A, Sridhar AR, Lanza GA, Cohoon K, Padmanabhan D, Pardo Gutierrez JA, Sinagra G, Merlo M, Zagari D, Rodriguez Escenaro BD, Pahlajani DB, Loncar G, Vukomanovic V, Jensen HK, Farkouh ME, Luescher TF, Su Ping CL, Peters NS, Friedman PA, Discover Consortium (DigitalNoninvasive Screening for COVID-19 with AI ECG Repository) (2021). Rapid exclusion of COVID infection with the artificial intelligence electrocardiogram. Mayo Clin Proc.

[78] Kashou AH, Medina-Inojosa JR, Noseworthy PA, Rodeheffer RJ, Lopez-Jimenez F, Attia IZ, Kapa S, Scott CG, Lee AT, Friedman PA, McKie PM (2021). Artificial intelligence-augmented electrocardiogram detection of left ventricular systolic dysfunction in the general population. Mayo Clin Proc.

[79] Kwon JM, Kim KH, Medina-Inojosa J, Jeon KH, Park J, Oh BH (2020). Artificial intelligence for early prediction of pulmonary hypertension using electrocardiography. J Heart Lung Transplant.

[80] Asch FM, Mor-Avi V, Rubenson D, Goldstein S, Saric M, Mikati I, Surette S, Chaudhry A, Poilvert N, Hong H, Horowitz R, Park D, Diaz-Gomez JL, Boesch B, Nikravan S, Liu RB, Philips C, Thomas JD, Martin RP, Lang RM (2021). Deep learning-based automated echocardiographic quantification of left ventricular ejection fraction: a point-of-care solution. Circ Cardiovasc Imaging.

[81] Kashou AH, Rabinstein AA, Attia IZ, Asirvatham SJ, Gersh BJ, Friedman PA, Noseworthy PA (2020). Recurrent cryptogenic stroke: a potential role for an artificial intelligence-enabled electrocardiogram?. HeartRhythm Case Rep.

[82] Wu L, He X, Liu M, Xie H, An P, Zhang J, Zhang H, Ai Y, Tong Q, Guo M, Huang M, Ge C, Yang Z, Yuan J, Liu J, Zhou W, Jiang X, Huang X, Mu G, Wan X, Li Y, Wang H, Wang Y, Zhang H, Chen D, Gong D, Wang J, Huang L, Li J, Yao L, Zhu Y, Yu H (2021). Evaluation of the effects of an artificial intelligence system on endoscopy quality and preliminary testing of its performance in detecting early gastric cancer: a randomized controlled trial. Endoscopy.

[83] Yang X, Wang H, Dong Q, Xu Y, Liu H, Ma X, Yan J, Li Q, Yang C, Li X (2022). An artificial intelligence system for distinguishing between gastrointestinal stromal tumors and leiomyomas using endoscopic ultrasonography. Endoscopy.

[84] Herrin J, Abraham NS, Yao X, Noseworthy PA, Inselman J, Shah ND, Ngufor C (2021). Comparative effectiveness of machine learning approaches for predicting gastrointestinal bleeds in patients receiving antithrombotic treatment. JAMA Netw Open.

[85] Xie X, Xiao YF, Zhao XY, Li JJ, Yang QQ, Peng X, Nie XB, Zhou JY, Zhao YB, Yang H, Liu X, Liu E, Chen YY, Zhou YY, Fan CQ, Bai JY, Lin H, Koulaouzidis A, Yang SM (2022). Development and validation of an artificial intelligence model for small bowel capsule endoscopy video review. JAMA Netw Open.

[86] Shung DL, Au B, Taylor RA, Tay JK, Laursen SB, Stanley AJ, Dalton HR, Ngu J, Schultz M, Laine L (2020). Validation of a machine learning model that outperforms clinical risk scoring systems for upper gastrointestinal bleeding. Gastroenterology.

[87] Bhuiyan A, Govindaiah A, Deobhakta A, Gupta M, Rosen R, Saleem S, Smith RT (2020). Development and validation of an automated diabetic retinopathy screening tool for primary care setting. Diabetes Care.

[88] Heydon P, Egan C, Bolter L, Chambers R, Anderson J, Aldington S, Stratton IM, Scanlon PH, Webster L, Mann S, du Chemin A, Owen CG, Tufail A, Rudnicka AR (2021). Prospective evaluation of an artificial intelligence-enabled algorithm for automated diabetic retinopathy screening of 30 000 patients. Br J Ophthalmol.

[89] Olvera-Barrios A, Heeren TF, Balaskas K, Chambers R, Bolter L, Egan C, Tufail A, Anderson J (2021). Diagnostic accuracy of diabetic retinopathy grading by an artificial intelligence-enabled algorithm compared with a human standard for wide-field true-colour confocal scanning and standard digital retinal images. Br J Ophthalmol.

[90] Dai L, Wu L, Li H, Cai C, Wu Q, Kong H, Liu R, Wang X, Hou X, Liu Y, Long X, Wen Y, Lu L, Shen Y, Chen Y, Shen D, Yang X, Zou H, Sheng B, Jia W (2021). A deep learning system for detecting diabetic retinopathy across the disease spectrum. Nat Commun.

[91] Ipp E, Liljenquist D, Bode B, Shah VN, Silverstein S, Regillo CD, Lim JI, Sadda S, Domalpally A, Gray G, Bhaskaranand M, Ramachandra C, Solanki K, EyeArt Study Group (2021). Pivotal evaluation of an artificial intelligence system for autonomous detection of referrable and vision-threatening diabetic retinopathy. JAMA Netw Open.

[92] Ravaut M, Harish V, Sadeghi H, Leung KK, Volkovs M, Kornas K, Watson T, Poutanen T, Rosella LC (2021). Development and validation of a machine learning model using administrative health data to predict onset of type 2 diabetes. JAMA Netw Open.

[93] Bachar N, Benbassat D, Brailovsky D, Eshel Y, Glück D, Levner D, Levy S, Pecker S, Yurkovsky E, Zait A, Sever C, Kratz A, Brugnara C (2021). An artificial intelligence-assisted diagnostic platform for rapid near-patient hematology. Am J Hematol.

[94] Dong L, He W, Zhang R, Ge Z, Wang YX, Zhou J, Xu J, Shao L, Wang Q, Yan Y, Xie Y, Fang L, Wang H, Wang Y, Zhu X, Wang J, Zhang C, Wang H, Wang Y, Chen R, Wan Q, Yang J, Zhou W, Li H, Yao X, Yang Z, Xiong J, Wang X, Huang Y, Chen Y, Wang Z, Rong C, Gao J, Zhang H, Wu S, Jonas JB, Wei WB (2022). Artificial intelligence for screening of multiple retinal and optic nerve diseases. JAMA Netw Open.

[95] Lee AY, Yanagihara RT, Lee CS, Blazes M, Jung HC, Chee YE, Gencarella MD, Gee H, Maa AY, Cockerham GC, Lynch M, Boyko EJ (2021). Multicenter, head-to-head, real-world validation study of seven automated artificial intelligence diabetic retinopathy screening systems. Diabetes Care.

[96] Lee Y, Kim G, Jun JE, Park H, Lee WJ, Hwang YC, Kim JH (2023). An integrated digital health care platform for diabetes management with ai-based dietary management: 48-week results from a randomized controlled trial. Diabetes Care.

[97] Oikonomidi T, Ravaud P, Cosson E, Montori V, Tran VT (2021). Evaluation of patient willingness to adopt remote digital monitoring for diabetes management. JAMA Netw Open.

[98] Repici A, Badalamenti M, Maselli R, Correale L, Radaelli F, Rondonotti E, Ferrara E, Spadaccini M, Alkandari A, Fugazza A, Anderloni A, Galtieri PA, Pellegatta G, Carrara S, Di Leo M, Craviotto V, Lamonaca L, Lorenzetti R, Andrealli A, Antonelli G, Wallace M, Sharma P, Rosch T, Hassan C (2020). Efficacy of real-time computer-aided detection of colorectal neoplasia in a randomized trial. Gastroenterology.

[99] Wang P, Liu P, Glissen Brown JR, Berzin TM, Zhou G, Lei S, Liu X, Li L, Xiao X (2020). Lower adenoma miss rate of computer-aided detection-assisted colonoscopy vs routine white-light colonoscopy in a prospective tandem study. Gastroenterology.

[100] Svoboda E (2020). Artificial intelligence is improving the detection of lung cancer. Nature.

[101] Song Z, Zou S, Zhou W, Huang Y, Shao L, Yuan J, Gou X, Jin W, Wang Z, Chen X, Ding X, Liu J, Yu C, Ku C, Liu C, Sun Z, Xu G, Wang Y, Zhang X, Wang D, Wang S, Xu W, Davis RC, Shi H (2020). Clinically applicable histopathological diagnosis system for gastric cancer detection using deep learning. Nat Commun.

[102] Qin ZZ, Ahmed S, Sarker MS, Paul K, Adel AS, Naheyan T, Barrett R, Banu S, Creswell J (2021). Tuberculosis detection from chest x-rays for triaging in a high tuberculosis-burden setting: an evaluation of five artificial intelligence algorithms. Lancet Digit Health.

[103] Tang LY, Coxson HO, Lam S, Leipsic J, Tam RC, Sin DD (2020). Towards large-scale case-finding: training and validation of residual networks for detection of chronic obstructive pulmonary disease using low-dose CT. Lancet Digit Health.

[104] Kim H, Kim HH, Han BK, Kim KH, Han K, Nam H, Lee EH, Kim E (2020). Changes in cancer detection and false-positive recall in mammography using artificial intelligence: a retrospective, multireader study. Lancet Digit Health.

[105] Wu S, Hong G, Xu A, Zeng H, Chen X, Wang Y, Luo Y, Wu P, Liu C, Jiang N, Dang Q, Yang C, Liu B, Shen R, Chen Z, Liao C, Lin Z, Wang J, Lin T (2023). Artificial intelligence-based model for lymph node metastases detection on whole slide images in bladder cancer: a retrospective, multicentre, diagnostic study. Lancet Oncol.

[106] Weigt J, Repici A, Antonelli G, Afifi A, Kliegis L, Correale L, Hassan C, Neumann H (2022). Performance of a new integrated computer-assisted system (CADe/CADx) for detection and characterization of colorectal neoplasia. Endoscopy.

[107] Homayounieh F, Digumarthy S, Ebrahimian S, Rueckel J, Hoppe BF, Sabel BO, Conjeti S, Ridder K, Sistermanns M, Wang L, Preuhs A, Ghesu F, Mansoor A, Moghbel M, Botwin A, Singh R, Cartmell S, Patti J, Huemmer C, Fieselmann A, Joerger C, Mirshahzadeh N, Muse V, Kalra M (2021). An artificial intelligence-based chest X-ray model on human nodule detection accuracy from a multicenter study. JAMA Netw Open.

[108] Glissen Brown JR, Mansour NM, Wang P, Chuchuca MA, Minchenberg SB, Chandnani M, Liu L, Gross SA, Sengupta N, Berzin TM (2022). Deep learning computer-aided polyp detection reduces adenoma miss rate: a united states multi-center randomized tandem colonoscopy study (CADeT-CS Trial). Clin Gastroenterol Hepatol.

[109] Foersch S, Eckstein M, Wagner DC, Gach F, Woerl AC, Geiger J, Glasner C, Schelbert S, Schulz S, Porubsky S, Kreft A, Hartmann A, Agaimy A, Roth W (2021). Deep learning for diagnosis and survival prediction in soft tissue sarcoma. Ann Oncol.

[110] Jin EH, Lee D, Bae JH, Kang HY, Kwak M, Seo JY, Yang JI, Yang SY, Lim SH, Yim JY, Lim JH, Chung GE, Chung SJ, Choi JM, Han YM, Kang SJ, Lee J, Chan Kim H, Kim JS (2020). Improved accuracy in optical diagnosis of colorectal polyps using convolutional neural networks with visual explanations. Gastroenterology.

[111] Shi Y, Wang Z, Chen P, Cheng P, Zhao K, Zhang H, Shu H, Gu L, Gao L, Wang Q, Zhang H, Xie C, Liu Y, Zhang Z, Alzheimer’s Disease Neuroimaging Initiative (2023). Episodic memory-related imaging features as valuable biomarkers for the diagnosis of Alzheimer’s disease: a multicenter study based on machine learning. Biol Psychiatry Cogn Neurosci Neuroimaging.

[112] Huang B, Tian S, Zhan N, Ma J, Huang Z, Zhang C, Zhang H, Ming F, Liao F, Ji M, Zhang J, Liu Y, He P, Deng B, Hu J, Dong W (2021). Accurate diagnosis and prognosis prediction of gastric cancer using deep learning on digital pathological images: a retrospective multicentre study. EBioMedicine.

[113] Jin C, Chen W, Cao Y, Xu Z, Tan Z, Zhang X, Deng L, Zheng C, Zhou J, Shi H, Feng J (2020). Development and evaluation of an artificial intelligence system for COVID-19 diagnosis. Nat Commun.

[114] Goh KH, Wang L, Yeow AY, Poh H, Li K, Yeow JJL, Tan GY (2021). Artificial intelligence in sepsis early prediction and diagnosis using unstructured data in healthcare. Nat Commun.

[115] Zhou Q, Zuley M, Guo Y, Yang L, Nair B, Vargo A, Ghannam S, Arefan D, Wu S (2021). A machine and human reader study on AI diagnosis model safety under attacks of adversarial images. Nat Commun.

[116] Peng S, Liu Y, Lv W, Liu L, Zhou Q, Yang H, Ren J, Liu G, Wang X, Zhang X, Du Q, Nie F, Huang G, Guo Y, Li J, Liang J, Hu H, Xiao H, Liu Z, Lai F, Zheng Q, Wang H, Li Y, Alexander EK, Wang W, Xiao H (2021). Deep learning-based artificial intelligence model to assist thyroid nodule diagnosis and management: a multicentre diagnostic study. Lancet Digit Health.

[117] Pantanowitz L, Quiroga-Garza GM, Bien L, Heled R, Laifenfeld D, Linhart C, Sandbank J, Albrecht Shach A, Shalev V, Vecsler M, Michelow P, Hazelhurst S, Dhir R (2020). An artificial intelligence algorithm for prostate cancer diagnosis in whole slide images of core needle biopsies: a blinded clinical validation and deployment study. Lancet Digit Health.

[118] Ström P, Kartasalo K, Olsson H, Solorzano L, Delahunt B, Berney DM, Bostwick DG, Evans AJ, Grignon DJ, Humphrey PA, Iczkowski KA, Kench JG, Kristiansen G, van der Kwast TH, Leite KR, McKenney JK, Oxley J, Pan C, Samaratunga H, Srigley JR, Takahashi H, Tsuzuki T, Varma M, Zhou M, Lindberg J, Lindskog C, Ruusuvuori P, Wählby C, Grönberg H, Rantalainen M, Egevad L, Eklund M (2020). Artificial intelligence for diagnosis and grading of prostate cancer in biopsies: a population-based, diagnostic study. Lancet Oncol.

[119] Venkatesan P (2021). Artificial intelligence and cancer diagnosis: caution needed. Lancet Oncol.

[120] Gao K, Su J, Jiang Z, Zeng L, Feng Z, Shen H, Rong P, Xu X, Qin J, Yang Y, Wang W, Hu D (2021). Dual-branch combination network (DCN): towards accurate diagnosis and lesion segmentation of COVID-19 using CT images. Med Image Anal.

[121] Pfob A, Sidey-Gibbons C, Barr RG, Duda V, Alwafai Z, Balleyguier C, Clevert D, Fastner S, Gomez C, Goncalo M, Gruber I, Hahn M, Hennigs A, Kapetas P, Lu S, Nees J, Ohlinger R, Riedel F, Rutten M, Schaefgen B, Stieber A, Togawa R, Tozaki M, Wojcinski S, Xu C, Rauch G, Heil J, Golatta M (2022). Intelligent multi-modal shear wave elastography to reduce unnecessary biopsies in breast cancer diagnosis (INSPiRED 002): a retrospective, international, multicentre analysis. Eur J Cancer.

[122] McKinney SM, Sieniek M, Godbole V, Godwin J, Antropova N, Ashrafian H, Back T, Chesus M, Corrado GS, Darzi A, Etemadi M, Garcia-Vicente F, Gilbert FJ, Halling-Brown M, Hassabis D, Jansen S, Karthikesalingam A, Kelly CJ, King D, Ledsam JR, Melnick D, Mostofi H, Peng L, Reicher JJ, Romera-Paredes B, Sidebottom R, Suleyman M, Tse D, Young KC, De Fauw J, Shetty S (2020). International evaluation of an AI system for breast cancer screening. Nature.

[123] Bachtiger P, Petri CF, Scott FE, Ri Park S, Kelshiker MA, Sahemey HK, Dumea B, Alquero R, Padam PS, Hatrick IR, Ali A, Ribeiro M, Cheung W, Bual N, Rana B, Shun-Shin M, Kramer DB, Fragoyannis A, Keene D, Plymen CM, Peters NS (2022). Point-of-care screening for heart failure with reduced ejection fraction using artificial intelligence during ECG-enabled stethoscope examination in London, UK: a prospective, observational, multicentre study. Lancet Digit Health.

[124] Kann BH, Likitlersuang J, Bontempi D, Ye Z, Aneja S, Bakst R, Kelly HR, Juliano AF, Payabvash S, Guenette JP, Uppaluri R, Margalit DN, Schoenfeld JD, Tishler RB, Haddad R, Aerts HJ, Garcia JJ, Flamand Y, Subramaniam RM, Burtness BA, Ferris RL (2023). Screening for extranodal extension in HPV-associated oropharyngeal carcinoma: evaluation of a CT-based deep learning algorithm in patient data from a multicentre, randomised de-escalation trial. Lancet Digit Health.

[125] Soltan AA, Kouchaki S, Zhu T, Kiyasseh D, Taylor T, Hussain ZB, Peto T, Brent AJ, Eyre DW, Clifton DA (2021). Rapid triage for COVID-19 using routine clinical data for patients attending hospital: development and prospective validation of an artificial intelligence screening test. Lancet Digit Health.

[126] Xie Y, Zhao L, Yang X, Wu X, Yang Y, Huang X, Liu F, Xu J, Lin L, Lin H, Feng Q, Lin H, Liu Q (2020). Screening candidates for refractive surgery with corneal tomographic-based deep learning. JAMA Ophthalmol.

[127] Abbasi J (2020). Artificial intelligence improves breast cancer screening in study. JAMA.

[128] Xu H, Tang RS, Lam TY, Zhao G, Lau JY, Liu Y, Wu Q, Rong L, Xu W, Li X, Wong SH, Cai S, Wang J, Liu G, Ma T, Liang X, Mak JW, Xu H, Yuan P, Cao T, Li F, Ye Z, Shutian Z, Sung JJ (2023). Artificial intelligence-assisted colonoscopy for colorectal cancer screening: a multicenter randomized controlled trial. Clin Gastroenterol Hepatol.

[129] Sun Y, Zhang L, Dong D, Li X, Wang J, Yin C, Poon LC, Tian J, Wu Q (2021). Application of an individualized nomogram in first-trimester screening for trisomy 21. Ultrasound Obstet Gynecol.

[130] Zeleznik R, Foldyna B, Eslami P, Weiss J, Alexander I, Taron J, Parmar C, Alvi RM, Banerji D, Uno M, Kikuchi Y, Karady J, Zhang L, Scholtz J, Mayrhofer T, Lyass A, Mahoney TF, Massaro JM, Vasan RS, Douglas PS, Hoffmann U, Lu MT, Aerts HJ (2021). Deep convolutional neural networks to predict cardiovascular risk from computed tomography. Nat Commun.

[131] Liu CM, Chang SL, Chen HH, Chen WS, Lin YJ, Lo LW, Hu YF, Chung FP, Chao TF, Tuan TC, Liao JN, Lin CY, Chang TY, Wu CI, Kuo L, Wu MH, Chen CK, Chang YY, Shiu YC, Lu HH, Chen SA (2020). The clinical application of the deep learning technique for predicting trigger origins in patients with paroxysmal atrial fibrillation with catheter ablation. Circ Arrhythm Electrophysiol.

[132] Qiang M, Li C, Sun Y, Sun Y, Ke L, Xie C, Zhang T, Zou Y, Qiu W, Gao M, Li Y, Li X, Zhan Z, Liu K, Chen X, Liang C, Chen Q, Mai H, Xie G, Guo X, Lv X (2021). A prognostic predictive system based on deep learning for locoregionally advanced nasopharyngeal carcinoma. J Natl Cancer Inst.

[133] She Y, He B, Wang F, Zhong Y, Wang T, Liu Z, Yang M, Yu B, Deng J, Sun X, Wu C, Hou L, Zhu Y, Yang Y, Hu H, Dong D, Chen C, Tian J (2022). Deep learning for predicting major pathological response to neoadjuvant chemoimmunotherapy in non-small cell lung cancer: a multicentre study. EBioMedicine.

[134] Wang L, Ding L, Liu Z, Sun L, Chen L, Jia R, Dai X, Cao J, Ye J (2020). Automated identification of malignancy in whole-slide pathological images: identification of eyelid malignant melanoma in gigapixel pathological slides using deep learning. Br J Ophthalmol.

[135] Li D, Bledsoe JR, Zeng Y, Liu W, Hu Y, Bi K, Liang A, Li S (2020). A deep learning diagnostic platform for diffuse large B-cell lymphoma with high accuracy across multiple hospitals. Nat Commun.

[136] Yu G, Sun K, Xu C, Shi XH, Wu C, Xie T, Meng R, Meng X, Wang K, Xiao H, Deng H (2021). Accurate recognition of colorectal cancer with semi-supervised deep learning on pathological images. Nat Commun.

[137] Kwon JM, Cho Y, Jeon KH, Cho S, Kim KH, Baek SD, Jeung S, Park J, Oh BH (2020). A deep learning algorithm to detect anaemia with ECGs: a retrospective, multicentre study. Lancet Digit Health.

[138] Lin A, Manral N, McElhinney P, Killekar A, Matsumoto H, Kwiecinski J, Pieszko K, Razipour A, Grodecki K, Park C, Otaki Y, Doris M, Kwan AC, Han D, Kuronuma K, Flores Tomasino G, Tzolos E, Shanbhag A, Goeller M, Marwan M, Gransar H, Tamarappoo BK, Cadet S, Achenbach S, Nicholls SJ, Wong DT, Berman DS, Dweck M, Newby DE, Williams MC, Slomka PJ, Dey D (2022). Deep learning-enabled coronary CT angiography for plaque and stenosis quantification and cardiac risk prediction: an international multicentre study. Lancet Digit Health.

[139] Storelli L, Azzimonti M, Gueye M, Vizzino C, Preziosa P, Tedeschi G, De Stefano N, Pantano P, Filippi M, Rocca MA (2022). A deep learning approach to predicting disease progression in multiple sclerosis using magnetic resonance imaging. Invest Radiol.

[140] Mao N, Zhang H, Dai Y, Li Q, Lin F, Gao J, Zheng T, Zhao F, Xie H, Xu C, Ma H (2023). Attention-based deep learning for breast lesions classification on contrast enhanced spectral mammography: a multicentre study. Br J Cancer.

[141] Ueno S, Berntsen J, Ito M, Uchiyama K, Okimura T, Yabuuchi A, Kato K (2021). Pregnancy prediction performance of an annotation-free embryo scoring system on the basis of deep learning after single vitrified-warmed blastocyst transfer: a single-center large cohort retrospective study. Fertil Steril.

[142] Yamashita R, Long J, Longacre T, Peng L, Berry G, Martin B, Higgins J, Rubin DL, Shen J (2021). Deep learning model for the prediction of microsatellite instability in colorectal cancer: a diagnostic study. Lancet Oncol.

[143] Li X, Gao H, Zhu J, Huang Y, Zhu Y, Huang W, Li Z, Sun K, Liu Z, Tian J, Li B (2021). 3D deep learning model for the pretreatment evaluation of treatment response in esophageal carcinoma: a prospective study (ChiCTR2000039279. Int J Radiat Oncol Biol Phys.

[144] Wu L, Ye W, Liu Y, Chen D, Wang Y, Cui Y, Li Z, Li P, Li Z, Liu Z, Liu M, Liang C, Yang X, Xie Y, Wang Y (2022). An integrated deep learning model for the prediction of pathological complete response to neoadjuvant chemotherapy with serial ultrasonography in breast cancer patients: a multicentre, retrospective study. Breast Cancer Res.

[145] Suri JS, Agarwal S, Saba L, Chabert GL, Carriero A, Paschè A, Danna P, Mehmedović A, Faa G, Jujaray T, Singh IM, Khanna NN, Laird JR, Sfikakis PP, Agarwal V, Teji JS, R Yadav R, Nagy F, Kincses ZT, Ruzsa Z, Viskovic K, Kalra MK (2022). Multicenter study on COVID-19 lung computed tomography segmentation with varying glass ground opacities using unseen deep learning artificial intelligence paradigms: COVLIAS 1.0 validation. J Med Syst.

[146] Khurshid S, Friedman S, Pirruccello JP, Di Achille P, Diamant N, Anderson CD, Ellinor PT, Batra P, Ho JE, Philippakis AA, Lubitz SA (2021). Deep learning to predict cardiac magnetic resonance-derived left ventricular mass and hypertrophy from 12-lead ECGs. Circ Cardiovasc Imaging.

[147] Liu XP, Jin X, Seyed Ahmadian S, Yang X, Tian SF, Cai YX, Chawla K, Snijders AM, Xia Y, van Diest PJ, Weiss WA, Mao J, Li Z, Vogel H, Chang H (2023). Clinical significance and molecular annotation of cellular morphometric subtypes in lower-grade gliomas discovered by machine learning. Neuro Oncol.

[148] Akal F, Batu ED, Sonmez HE, Karadağ ŞG, Demir F, Ayaz NA, Sözeri B (2022). Diagnosing growing pains in children by using machine learning: a cross-sectional multicenter study. Med Biol Eng Comput.

[149] Awada H, Durmaz A, Gurnari C, Kishtagari A, Meggendorfer M, Kerr CM, Kuzmanovic T, Durrani J, Shreve J, Nagata Y, Radivoyevitch T, Advani AS, Ravandi F, Carraway HE, Nazha A, Haferlach C, Saunthararajah Y, Scott J, Visconte V, Kantarjian H, Kadia T, Sekeres MA, Haferlach T, Maciejewski JP (2021). Machine learning integrates genomic signatures for subclassification beyond primary and secondary acute myeloid leukemia. Blood.

[150] Moyer JD, Lee P, Bernard C, Henry L, Lang E, Cook F, Planquart F, Boutonnet M, Harrois A, Gauss T, Traumabase Group® (2022). Machine learning-based prediction of emergency neurosurgery within 24 h after moderate to severe traumatic brain injury. World J Emerg Surg.

[151] Hollon T, Jiang C, Chowdury A, Nasir-Moin M, Kondepudi A, Aabedi A, Adapa A, Al-Holou W, Heth J, Sagher O, Lowenstein P, Castro M, Wadiura LI, Widhalm G, Neuschmelting V, Reinecke D, von Spreckelsen N, Berger MS, Hervey-Jumper SL, Golfinos JG, Snuderl M, Camelo-Piragua S, Freudiger C, Lee H, Orringer DA (2023). Artificial-intelligence-based molecular classification of diffuse gliomas using rapid, label-free optical imaging. Nat Med.

[152] Takenaka K, Ohtsuka K, Fujii T, Negi M, Suzuki K, Shimizu H, Oshima S, Akiyama S, Motobayashi M, Nagahori M, Saito E, Matsuoka K, Watanabe M (2020). Development and validation of a deep neural network for accurate evaluation of endoscopic images from patients with ulcerative colitis. Gastroenterology.

[153] Savage N (2023). Why artificial intelligence needs to understand consequences. Nature (Forthcoming).

[154] - (2021). Artificial intelligence predicts drug response. Cancer Discov.

[155] Wagner M, Müller-Stich BP, Kisilenko A, Tran D, Heger P, Mündermann L, Lubotsky DM, Müller B, Davitashvili T, Capek M, Reinke A, Reid C, Yu T, Vardazaryan A, Nwoye CI, Padoy N, Liu X, Lee E, Disch C, Meine H, Xia T, Jia F, Kondo S, Reiter W, Jin Y, Long Y, Jiang M, Dou Q, Heng PA, Twick I, Kirtac K, Hosgor E, Bolmgren JL, Stenzel M, von Siemens B, Zhao L, Ge Z, Sun H, Xie D, Guo M, Liu D, Kenngott HG, Nickel F, Frankenberg MV, Mathis-Ullrich F, Kopp-Schneider A, Maier-Hein L, Speidel S, Bodenstedt S (2023). Comparative validation of machine learning algorithms for surgical workflow and skill analysis with the HeiChole benchmark. Med Image Anal.

[156] Soda P, D'Amico NC, Tessadori J, Valbusa G, Guarrasi V, Bortolotto C, Akbar MU, Sicilia R, Cordelli E, Fazzini D, Cellina M, Oliva G, Callea G, Panella S, Cariati M, Cozzi D, Miele V, Stellato E, Carrafiello G, Castorani G, Simeone A, Preda L, Iannello G, Del Bue A, Tedoldi F, Alí M, Sona D, Papa S (2021). AIforCOVID: predicting the clinical outcomes in patients with COVID-19 applying AI to chest-X-rays. An Italian multicentre study. Med Image Anal.

[157] Avari P, Leal Y, Herrero P, Wos M, Jugnee N, Arnoriaga-Rodríguez M, Thomas M, Liu C, Massana Q, Lopez B, Nita L, Martin C, Fernández-Real JM, Oliver N, Fernández-Balsells M, Reddy M (2021). Safety and feasibility of the PEPPER adaptive bolus advisor and safety system: a randomized control study. Diabetes Technol Ther.

[158] Wathour J, Govaerts PJ, Deggouj N (2020). From manual to artificial intelligence fitting: two cochlear implant case studies. Cochlear Implants Int.

[159] Jayakumar P, Moore MG, Furlough KA, Uhler LM, Andrawis JP, Koenig KM, Aksan N, Rathouz PJ, Bozic KJ (2021). Comparison of an artificial intelligence-enabled patient decision aid vs educational material on decision quality, shared decision-making, patient experience, and functional outcomes in adults with knee osteoarthritis: a randomized clinical trial. JAMA Netw Open.

[160] Eilts SK, Pfeil JM, Poschkamp B, Krohne TU, Eter N, Barth T, Guthoff R, Lagrèze W, Grundel M, Bründer MC, Busch M, Kalpathy-Cramer J, Chiang MF, Chan RV, Coyner AS, Ostmo S, Campbell JP, Stahl A, Comparing Alternative Ranibizumab Dosages for SafetyEfficacy in Retinopathy of Prematurity (CARE-ROP) Study Group (2023). Assessment of retinopathy of prematurity regression and reactivation using an artificial intelligence-based vascular severity score. JAMA Netw Open.

[161] Takeda I, Yamada A, Onodera H (2021). Artificial intelligence-assisted motion capture for medical applications: a comparative study between markerless and passive marker motion capture. Comput Methods Biomech Biomed Engin.

[162] Nimri R, Battelino T, Laffel LM, Slover RH, Schatz D, Weinzimer SA, Dovc K, Danne T, Phillip M, NextDREAM Consortium (2020). Insulin dose optimization using an automated artificial intelligence-based decision support system in youths with type 1 diabetes. Nat Med.

[163] Carvalho DM, Richardson PJ, Olaciregui N, Stankunaite R, Lavarino C, Molinari V, Corley EA, Smith DP, Ruddle R, Donovan A, Pal A, Raynaud FI, Temelso S, Mackay A, Overington JP, Phelan A, Sheppard D, Mackinnon A, Zebian B, Al-Sarraj S, Merve A, Pryce J, Grill J, Hubank M, Cruz O, Morales La Madrid A, Mueller S, Carcaboso AM, Carceller F, Jones C (2022). Repurposing Vandetanib plus everolimus for the treatment of -mutant diffuse intrinsic pontine glioma. Cancer Discov.

[164] Sheridan C (2020). Massive data initiatives and AI provide testbed for pandemic forecasting. Nat Biotechnol.

[165] Meeuws M, Pascoal D, Janssens de Varebeke S, De Ceulaer G, Govaerts PJ (2020). Cochlear implant telemedicine: remote fitting based on psychoacoustic self-tests and artificial intelligence. Cochlear Implants Int.

[166] Thomas J, Harden A (2008). Methods for the thematic synthesis of qualitative research in systematic reviews. BMC Med Res Methodol.

[167] Dong Q, Li L, Dai D, Zheng C, Wu Z, Chang B, Sun X, Xu J, Li L, Sui Z (2022). A survey for in-context learning. arXiv. Preprint posted online December 31, 2022.

[168] Chi EA, Chi G, Tsui CT, Jiang Y, Jarr K, Kulkarni CV, Zhang M, Long J, Ng AY, Rajpurkar P, Sinha SR (2021). Development and validation of an artificial intelligence system to optimize clinician review of patient records. JAMA Netw Open.

[169] Cowan RP, Rapoport AM, Blythe J, Rothrock J, Knievel K, Peretz AM, Ekpo E, Sanjanwala BM, Woldeamanuel YW (2022). Diagnostic accuracy of an artificial intelligence online engine in migraine: a multi-center study. Headache.

[170] Curran JM, Meuter ML, Surprenant CF (2016). Intentions to use self-service technologies: a confluence of multiple attitudes. J Serv Res.

[171] Dabholkar PA (1996). Consumer evaluations of new technology-based self-service options: an investigation of alternative models of service quality. Int J Res Mark.

[172] Seneviratne MG, Li RC, Schreier M, Lopez-Martinez D, Patel BS, Yakubovich A, Kemp JB, Loreaux E, Gamble P, El-Khoury K, Vardoulakis L, Wong D, Desai J, Chen JH, Morse KE, Downing NL, Finger LT, Chen M, Shah N (2022). User-centred design for machine learning in health care: a case study from care management. BMJ Health Care Inform.

[173] Giordano C, Brennan M, Mohamed B, Rashidi P, Modave F, Tighe P (2021). Accessing artificial intelligence for clinical decision-making. Front Digit Health.

[174] Novak LL, Russell RG, Garvey K, Patel M, Thomas Craig KJ, Snowdon J, Miller B (2023). Clinical use of artificial intelligence requires AI-capable organizations. JAMIA Open.

