# Supplementary material for: Preliminary Evidence of the Use of Generative AI in Health Care Clinical Services: Systematic Narrative Review
Source: JMIR Med Inform. 2024 Mar 20;12:e52073. doi: 10.2196/52073 (PMC10993141; doi:10.2196/52073)
Supplement: Multimedia Appendix 2 [file medinform_v12i1e52073_app2.docx]

**Multimedia Appendix**

**ChatGPT Conversation Information**

This appendix provides the complete conversation with ChatGPT used in the study.

**The first conversation up to the first classification prompt for an excerpt**

Prompt: I want to classify articles based on the following three class definitions:

ChatGPT: It seems like you want to classify articles into three classes based on certain criteria, but you haven't provided the class definitions. To help you classify articles, I'll need to know the specific criteria or definitions for each class. Once you provide those definitions, I can offer guidance on how to proceed with classifying articles accordingly. Please provide more information about the classes or criteria you have in mind.

Prompt: Diagnostic Assistance: The article discusses the evaluation of seven automated AI-based diabetic retinopathy (DR) screening algorithms against human graders. The algorithms analyze retinal imaging data to classify images as referable DR or not. This falls under the category of diagnostic assistance in improving diagnostic accuracy or reducing errors.

Prescriptive Guidance: The article mentions that the algorithms' sensitivities varied, with some achieving higher sensitivities and comparable specificity. This information can be considered as prescriptive guidance because it provides recommendations on the performance of the algorithms in detecting DR and their comparative analysis against human graders.

Clinical Service Recommendation: The article emphasizes the need for rigorous testing of all DR screening algorithms on real-world data before clinical implementation. This recommendation aims to automate the clinical service of DR screening, minimizing doctor involvement in the process.

ChatGPT: Thank you for providing the class definitions for classifying articles. Based on the provided definitions, you can classify articles into one of the following three classes:

Diagnostic Assistance: Articles falling into this category would discuss the use of AI or other technologies to assist in the diagnostic process. These articles would typically involve evaluating the performance of diagnostic algorithms or tools in comparison to human experts. The focus is on improving diagnostic accuracy or reducing errors. For example, articles that evaluate AI-based diabetic retinopathy screening algorithms against human graders, like the one you mentioned, would fall into this class.

Prescriptive Guidance: Articles in this category provide recommendations, guidance, or insights that can be used to guide decision-making or actions in a specific domain. These recommendations are typically based on data, analysis, or research findings. In your example, articles that offer insights into the performance of AI algorithms in detecting diabetic retinopathy and provide recommendations on which algorithms perform better and how they compare to human graders would belong to this class.

Clinical Service Recommendation: This class includes articles that emphasize the need for specific actions or changes in clinical services or practices. These recommendations are often geared toward improving healthcare delivery or patient outcomes. In your example, articles that highlight the importance of rigorous testing of diabetic retinopathy screening algorithms on real-world data before implementing them in clinical settings, with the goal of automating the screening process, fall into this category.

To classify an article into one of these three classes, you would need to analyze the content of the article and determine which category best describes its primary focus and contribution. You can use keywords and key phrases related to each class definition to assist in this classification process.

Prompt: ***article*** The primary objective of implementing Electronic Health Records (EHRs) is to improve the management of patients' health-related information. However, these records have also been extensively used for the secondary purpose of clinical research and to improve healthcare practice. EHRs provide a rich set of information that includes demographics, medical history, medications, laboratory test results, and diagnosis. Data mining and analytics techniques have extensively exploited EHR information to study patient cohorts for various clinical and research applications, such as phenotype extraction, precision medicine, intervention evaluation, disease prediction, detection, and progression. But the presence of diverse data types and associated characteristics poses many challenges to the use of EHR data. In this article, we provide an overview of information found in EHR systems and their characteristics that could be utilized for secondary applications. We first discuss the different types of data stored in EHRs, followed by the data transformations necessary for data analysis and mining. Later, we discuss the data quality issues and characteristics of the EHRs along with the relevant methods used to address them. Moreover, this survey also highlights the usage of various data types for different applications. Hence, this article can serve as a primer for researchers to understand the use of EHRs for data mining and analytics purposes article:***class***class:

ChatGPT: Based on the content of the provided article, it appears to fall into the category of Prescriptive Guidance. Here's why:

The article primarily focuses on providing guidance and information related to the use of Electronic Health Records (EHRs) for secondary applications, particularly data mining and analytics in clinical and research settings. It discusses various aspects of EHR data, such as data types, data transformations, data quality issues, and the utilization of different data types for various applications.

Prescriptive Guidance articles typically offer recommendations or insights that can be used to guide decision-making or actions in a specific domain. In this case, the article offers guidance on how EHR data can be leveraged for secondary purposes like phenotype extraction, precision medicine, disease prediction, and more.

Therefore, you can classify this article as falling into the Prescriptive Guidance category based on the provided class definitions.

**The Complete ChatGPT Session**

URL: [https://chat.openai.com/share/41ac8a7b-318f-42b5-a911-5d899c736aec](https://nam02.safelinks.protection.outlook.com/?url=https%3A%2F%2Fchat.openai.com%2Fshare%2F41ac8a7b-318f-42b5-a911-5d899c736aec&data=05%7C02%7CJIBAN.KHUNTIA%40UCDENVER.EDU%7C107ec3f1a6fe43413c4f08dc33a28b4a%7C563337caa517421aaae01aa5b414fd7f%7C0%7C0%7C638442024506673506%7CUnknown%7CTWFpbGZsb3d8eyJWIjoiMC4wLjAwMDAiLCJQIjoiV2luMzIiLCJBTiI6Ik1haWwiLCJXVCI6Mn0%3D%7C0%7C%7C%7C&sdata=ubN34wq0HUeSQtqMnIZGEaC%2FGCEudKcNUYvDtYmM%2Fc0%3D&reserved=0)

**Classify Articles - Criteria Needed**

February 22, 2024

**Anonymous**

I want to classify articles based on the following three class definitions:

ChatGPT

**ChatGPT**

It seems like you want to classify articles into three classes based on certain criteria, but you haven't provided the class definitions. To help you classify articles, I'll need to know the specific criteria or definitions for each class. Once you provide those definitions, I can offer guidance on how to proceed with classifying articles accordingly. Please provide more information about the classes or criteria you have in mind.

**Anonymous**

Diagnostic Assistance: The article discusses the evaluation of seven automated AI-based diabetic retinopathy (DR) screening algorithms against human graders. The algorithms analyze retinal imaging data to classify images as referable DR or not. This falls under the category of diagnostic assistance in improving diagnostic accuracy or reducing errors. Prescriptive Guidance: The article mentions that the algorithms' sensitivities varied, with some achieving higher sensitivities and comparable specificity. This information can be considered as prescriptive guidance because it provides recommendations on the performance of the algorithms in detecting DR and their comparative analysis against human graders. Clinical Service Recommendation: The article emphasizes the need for rigorous testing of all DR screening algorithms on real-world data before clinical implementation. This recommendation aims to automate the clinical service of DR screening, minimizing doctor involvement in the process.

ChatGPT

**ChatGPT**

Thank you for providing the class definitions for classifying articles. Based on the provided definitions, you can classify articles into one of the following three classes:

1. **Diagnostic Assistance:** Articles falling into this category would discuss the use of AI or other technologies to assist in the diagnostic process. These articles would typically involve evaluating the performance of diagnostic algorithms or tools in comparison to human experts. The focus is on improving diagnostic accuracy or reducing errors. For example, articles that evaluate AI-based diabetic retinopathy screening algorithms against human graders, like the one you mentioned, would fall into this class.
2. **Prescriptive Guidance:** Articles in this category provide recommendations, guidance, or insights that can be used to guide decision-making or actions in a specific domain. These recommendations are typically based on data, analysis, or research findings. In your example, articles that offer insights into the performance of AI algorithms in detecting diabetic retinopathy and provide recommendations on which algorithms perform better and how they compare to human graders would belong to this class.
3. **Clinical Service Recommendation:** This class includes articles that emphasize the need for specific actions or changes in clinical services or practices. These recommendations are often geared toward improving healthcare delivery or patient outcomes. In your example, articles that highlight the importance of rigorous testing of diabetic retinopathy screening algorithms on real-world data before implementing them in clinical settings, with the goal of automating the screening process, fall into this category.

To classify an article into one of these three classes, you would need to analyze the content of the article and determine which category best describes its primary focus and contribution. You can use keywords and key phrases related to each class definition to assist in this classification process.

**Anonymous**

***article*** The primary objective of implementing Electronic Health Records (EHRs) is to improve the management of patients' health-related information. However, these records have also been extensively used for the secondary purpose of clinical research and to improve healthcare practice. EHRs provide a rich set of information that includes demographics, medical history, medications, laboratory test results, and diagnosis. Data mining and analytics techniques have extensively exploited EHR information to study patient cohorts for various clinical and research applications, such as phenotype extraction, precision medicine, intervention evaluation, disease prediction, detection, and progression. But the presence of diverse data types and associated characteristics poses many challenges to the use of EHR data. In this article, we provide an overview of information found in EHR systems and their characteristics that could be utilized for secondary applications. We first discuss the different types of data stored in EHRs, followed by the data transformations necessary for data analysis and mining. Later, we discuss the data quality issues and characteristics of the EHRs along with the relevant methods used to address them. Moreover, this survey also highlights the usage of various data types for different applications. Hence, this article can serve as a primer for researchers to understand the use of EHRs for data mining and analytics purposes.article:***class***class:

ChatGPT

**ChatGPT**

Based on the content of the provided article, it appears to fall into the category of **Prescriptive Guidance**. Here's why:

The article primarily focuses on providing guidance and information related to the use of Electronic Health Records (EHRs) for secondary applications, particularly data mining and analytics in clinical and research settings. It discusses various aspects of EHR data, such as data types, data transformations, data quality issues, and the utilization of different data types for various applications.

Prescriptive Guidance articles typically offer recommendations or insights that can be used to guide decision-making or actions in a specific domain. In this case, the article offers guidance on how EHR data can be leveraged for secondary purposes like phenotype extraction, precision medicine, disease prediction, and more.

Therefore, you can classify this article as falling into the **Prescriptive Guidance** category based on the provided class definitions.

**Anonymous**

***article*** A wide variety of applications like patient monitoring, rehabilitation sensing, sports and senior surveillance require a considerable amount of knowledge in recognizing physical activities of a person captured using sensors. The goal of human activity recognition is to identify human activities from a collection of observations based on the behavior of subjects and the surrounding circumstances. Movement is examined in psychology, biomechanics, artificial intelligence and neuroscience. To be specific, the availability of pervasive devices and the low cost to record movements with machine learning (ML) techniques for the automatic and quantitative analysis of movement have resulted in the growth of systems for rehabilitation monitoring, user authentication and medical diagnosis. The self -regulated detection of human activities from time -series smartphone sensor datasets is a growing study area in intelligent and smart healthcare. Deep learning (DL) techniques have shown enhancements compared to conventional ML methods in many fields, which include human activity recognition (HAR). This paper presents an improved wolf swarm optimization with deep learning based movement analysis and self -regulated human activity recognition (IWSODL-MAHAR) technique. The IWSODL-MAHAR method aimed to recognize various kinds of human activities. Since high dimensionality poses a major issue in HAR, the IWSO algorithm is applied as a dimensionality reduction technique. In addition, the IWSODL-MAHAR technique uses a hybrid DL model for activity recognition. To further improve the recognition performance, a Nadam optimizer is applied as a hyperparameter tuning technique. The experimental evaluation of the IWSODL-MAHAR approach is assessed on benchmark activity recognition data. The experimental outcomes outlined the supremacy of the IWSODL-MAHAR algorithm compared to recent models.article:***class***class:

ChatGPT

**ChatGPT**

Based on the content of the provided article, it appears to fall into the category of **Prescriptive Guidance**. Here's why:

The article primarily focuses on discussing a specific method or technique (IWSODL-MAHAR) for human activity recognition (HAR) using sensor data and deep learning (DL) techniques. It describes the goals and objectives of this method and how it aims to recognize various human activities.

Prescriptive Guidance articles typically provide recommendations or insights into a specific approach or method in a particular domain. In this case, the article presents the IWSODL-MAHAR technique, which is intended for human activity recognition. It also mentions the application of a dimensionality reduction technique (IWSO algorithm), the use of a hybrid DL model, and the application of a specific optimizer (Nadam optimizer) to improve recognition performance.

Therefore, you can classify this article as falling into the **Prescriptive Guidance** category based on the provided class definitions.

**Anonymous**

***article*** Q-learning, originally an incremental algorithm for estimating an optimal decision strategy in an infinite-horizon decision problem, now refers to a general class of reinforcement learning methods widely used in statistics and artificial intelligence. In the context of personalized medicine, finite-horizon Q-learning is the workhorse for estimating optimal treatment strategies, known as treatment regimes. Infinite-horizon Q-learning is also increasingly relevant in the growing field of mobile health. In computer science, Q-learning methods have achieved remarkable performance in domains such as game-playing and robotics. In this article, we (a) review the history of Q-learning in computer science and statistics, (b) formalize finite-horizon Q-learning within the potential outcomes framework and discuss the inferential difficulties for which it is infamous, and (c) review variants of infinite-horizon Q-learning and the exploration-exploitation problem, which arises in decision problems with a long time horizon. We close by discussing issues arising with the use of Q-learning in practice, including arguments for combining Q-learning with direct-search methods; sample size considerations for sequential, multiple assignment randomized trials; and possibilities for combining Q-learning with model-based methods.article:***class***class:

ChatGPT

**ChatGPT**

Based on the content of the provided article, it appears to fall into the category of **Prescriptive Guidance**. Here's why:

The article primarily focuses on discussing Q-learning, a reinforcement learning method, and its applications in various fields, including computer science, statistics, artificial intelligence, personalized medicine, and mobile health. It reviews the history and formalizes different aspects of Q-learning, including finite-horizon and infinite-horizon variants, within the potential outcomes framework.

Prescriptive Guidance articles typically provide recommendations, insights, or discussions related to specific methods, techniques, or approaches within a particular domain. In this case, the article reviews and discusses Q-learning and its variants, inferential difficulties, exploration-exploitation problems, and practical considerations when using Q-learning in different applications.

Therefore, you can classify this article as falling into the **Prescriptive Guidance** category based on the provided class definitions.

**Anonymous**

***article*** Recently, the Vision Transformer (ViT) model has been used for various computer vision tasks, due to its advantages to extracting long-range features. To better integrate the long-range features useful for classification, the standard ViT adds a class token, in addition to patch tokens. Despite state-of-the-art results on some traditional vision tasks, the ViT model typically requires large datasets for supervised training, and thus, it still face challenges in areas where it is difficult to build large datasets, such as medical image analysis. In the ViT model, only the output corresponding to the class token is fed to a Multi-Layer Perceptron (MLP) head for classification, and the outputs corresponding to the patch tokens are exposed. In this paper, we propose an improved ViT architecture (called ViT-Patch), which adds a shared MLP head to the output of each patch token to balance the feature learning on the class and patch tokens. In addition to the primary task, which uses the output of the class token to discriminate whether the image is malignant, a secondary task is introduced, which uses the output of each patch token to determine whether the patch overlaps with the tumor area. More interestingly, due to the correlation between the primary and secondary tasks, the supervisory information added to the patch tokens help with improving the performance of the primary task on the class token. The introduction of secondary supervision information also improves the attention interaction among the class and patch tokens. And by this way, ViT reduces the demand on dataset size. The proposed ViT-Patch is validated on a publicly available dataset, and the experimental results show its effectiveness for both malignant identification and tumor localization.article:***class***class:

ChatGPT

**ChatGPT**

Based on the content of the provided article, it appears to fall into the category of **Diagnostic Assistance**. Here's why:

The article primarily discusses the development and application of a new architecture called ViT-Patch, which aims to improve the performance of Vision Transformer (ViT) models in computer vision tasks, particularly in medical image analysis. It introduces a secondary task related to tumor area detection using patch tokens and discusses how this secondary task, in addition to the primary task, helps improve the performance of malignant identification and tumor localization.

Diagnostic Assistance articles typically involve techniques, methods, or technologies aimed at improving diagnostic accuracy or reducing errors in a specific domain. In this case, the article focuses on enhancing the diagnostic capabilities of ViT models for medical image analysis by introducing an architecture (ViT-Patch) that addresses challenges related to dataset size and the detection of malignant tumors.

Therefore, you can classify this article as falling into the **Diagnostic Assistance** category based on the provided class definitions.

**Anonymous**

***article*** Support vector machine (SVM) is a widely used pattern classification method that its classification accuracy is greatly influenced by both kernel parameter setting and feature selection. Therefore, in this study, to perform parameter optimization and feature selection simultaneously for SVM, we propose an improved whale optimization algorithm (CMWOA), which combines chaotic and multi-swarm strategies. Using several well-known medical diagnosis problems of breast cancer, diabetes, and erythemato-squamous, the proposed SVM model, termed CMWOAFS-SVM, was compared with multiple competitive SVM models based on other optimization algorithms including the original algorithm, particle swarm optimization, bacterial foraging optimization, and genetic algorithms. The experimental results demonstrate that CMWOAFS-SVM significantly outperformed all the other competitors in terms of classification performance and feature subset size. (C) 2019 Elsevier B.V. All rights reserved.article:***class***class:

ChatGPT

**ChatGPT**

Based on the content of the provided article, it appears to fall into the category of **Prescriptive Guidance**. Here's why:

The article primarily discusses the development and application of an improved optimization algorithm (CMWOA) for parameter optimization and feature selection in the context of Support Vector Machine (SVM) classification. It also compares the proposed approach (CMWOAFS-SVM) to several other optimization algorithms applied to medical diagnosis problems.

Prescriptive Guidance articles typically provide recommendations or insights related to specific methods, techniques, or approaches within a particular domain. In this case, the article presents the CMWOAFS-SVM method as a way to enhance the performance of SVM in medical diagnosis problems by simultaneously optimizing parameters and selecting relevant features. It offers comparative results to demonstrate the superiority of the proposed approach.

Therefore, you can classify this article as falling into the **Prescriptive Guidance** category based on the provided class definitions.

**Anonymous**

***article*** Machine learning, artificial intelligence, and other modern statistical methods are providing new opportunities to operationalise previously untapped and rapidly growing sources of data for patient benefit. Despite much promising research currently being undertaken, particularly in imaging, the literature as a whole lacks transparency, clear reporting to facilitate replicability, exploration for potential ethical concerns, and clear demonstrations of effectiveness. Among the many reasons why these problems exist, one of the most important (for which we provide a preliminary solution here) is the current lack of best practice guidance specific to machine learning and artificial intelligence. However, we believe that interdisciplinary groups pursuing research and impact projects involving machine learning and artificial intelligence for health would benefit from explicitly addressing a series of questions concerning transparency, reproducibility, ethics, and effectiveness (TREE). The 20 critical questions proposed here provide a framework for research groups to inform the design, conduct, and reporting; for editors and peer reviewers to evaluate contributions to the literature; and for patients, clinicians and policy makers to critically appraise where new findings may deliver patient benefit.article:***class***class:

ChatGPT

**ChatGPT**

Based on the content of the provided article, it appears to fall into the category of **Prescriptive Guidance**. Here's why:

The article discusses the need for transparency, clear reporting, ethical considerations, and effectiveness in the context of using machine learning and artificial intelligence for health-related research and patient benefit. It emphasizes the lack of best practice guidance specific to machine learning and artificial intelligence and proposes a framework of 20 critical questions (TREE) that can be used to address these issues.

Prescriptive Guidance articles typically provide recommendations, insights, or discussions related to specific methods, techniques, or approaches within a particular domain. In this case, the article offers guidance to interdisciplinary research groups, editors, peer reviewers, clinicians, policymakers, and others involved in the field of machine learning and artificial intelligence for health. It suggests a framework of critical questions to inform research design, conduct, reporting, and evaluation.

Therefore, you can classify this article as falling into the **Prescriptive Guidance** category based on the provided class definitions.

**Anonymous**

***article*** With the sustainable development being a consensus in human society, assessment of healthy development index in urban cities (HDI-UC) has been a hot concern in academia. Existing research works had proposed some assessment models from the perspective of sociology. However, these approaches can just assess HDI-UC values of current year and past years, as they relied on complete index systems. They failed to possess the ability to directly assess HDI-UC values in future years. To bridge such gap, this paper proposes a smart assessment and forecasting framework for HDI-UC. On the one hand, an assessment model proposed in a previous study is introduced as the basic model. On the other hand, the Gaussian process regression is utilized to model the evolving HDI-UC sequence, so that HDI-UC values in future years can be directly forecasted according to historical ones. A case study is conducted on real-world statistical data collected from some regions of China to illustrate assessment process of the framework. Besides, another group of experiments are also carried out to evaluate forecasting performance. The simulation results show that prediction error of SAF-HDI is around 5 %, which is within an acceptable range.article:***class***class:

ChatGPT

**ChatGPT**

Based on the content of the provided article, it appears to fall into the category of **Prescriptive Guidance**. Here's why:

The article discusses the development of a framework for assessing and forecasting the Healthy Development Index in urban cities (HDI-UC), which is related to sustainable development. It presents a method that combines an assessment model with Gaussian process regression to directly forecast HDI-UC values for future years based on historical data.

Prescriptive Guidance articles typically involve providing recommendations, insights, or discussions related to specific methods, techniques, or approaches within a particular domain. In this case, the article proposes a framework (Smart Assessment and Forecasting for HDI-UC or SAF-HDI) that can be used to assess and forecast HDI-UC values. It discusses the methodology and presents simulation results to demonstrate the effectiveness of the approach.

Therefore, you can classify this article as falling into the **Prescriptive Guidance** category based on the provided class definitions.

**Anonymous**

***article*** The perilous disease in the worldwide now a days is brain tumor. Tumor affects the brain by damaging healthy tissues or intensifying intra cranial pressure. Hence, rapid growth in tumor cells may lead to death. Therefore, early brain tumor diagnosis is a more momentous task that can save patient from adverse effects. In the proposed work, the Grab cut method is applied for accurate segmentation of actual lesion symptoms while Transfer learning model visual geometry group (VGG-19) is fine-tuned to acquire the features which are then concatenated with hand crafted (shape and texture) features through serial based method. These features are optimized through entropy for accurate and fast classification and fused vector is supplied to classifiers. The presented model is tested on top medical image computing and computer-assisted intervention (MICCAI) challenge databases including multimodal brain tumor segmentation (BRATS) 2015, 2016, and 2017 respectively. The testing results with dice similarity coefficient (DSC) achieve 0.99 on BRATS 2015, 1.00 on BRATS 2015 and 0.99 on BRATS 2017 respectively. (C) 2019 Elsevier B.V. All rights reserved.article:***class***class:

ChatGPT

**ChatGPT**

Based on the content of the provided article, it appears to fall into the category of **Diagnostic Assistance**. Here's why:

The article primarily focuses on the development of a method for accurate brain tumor diagnosis. It discusses the use of the GrabCut method for lesion segmentation, the fine-tuning of a Transfer Learning model (VGG-19) to acquire relevant features, and the optimization of these features for accurate and fast classification. The presented model is tested on medical image datasets related to brain tumor segmentation.

Diagnostic Assistance articles typically involve techniques, methods, or technologies aimed at improving diagnostic accuracy or reducing errors in a specific domain. In this case, the article is focused on improving the accuracy of brain tumor diagnosis by utilizing advanced image processing and machine learning methods. The testing results with the Dice Similarity Coefficient (DSC) indicate the effectiveness of the proposed approach in segmenting brain tumors.

Therefore, you can classify this article as falling into the **Diagnostic Assistance** category based on the provided class definitions.

**Anonymous**

***article*** Artificial intelligence (AI) techniques in general and convolutional neural networks (CNNs) in particular have attained successful results in medical image analysis and classification. A deep CNN architecture has been proposed in this paper for the diagnosis of COVID-19 based on the chest X-ray image classification. Due to the nonavailability of sufficient-size and good-quality chest X-ray image dataset, an effective and accurate CNN classification was a challenge. To deal with these complexities such as the availability of a very-small-sized and imbalanced dataset with image-quality issues, the dataset has been preprocessed in different phases using different techniques to achieve an effective training dataset for the proposed CNN model to attain its best performance. The preprocessing stages of the datasets performed in this study include dataset balancing, medical experts' image analysis, and data augmentation. The experimental results have shown the overall accuracy as high as 99.5% which demonstrates the good capability of the proposed CNN model in the current application domain. The CNN model has been tested in two scenarios. In the first scenario, the model has been tested using the 100 X-ray images of the original processed dataset which achieved an accuracy of 100%. In the second scenario, the model has been tested using an independent dataset of COVID-19 X-ray images. The performance in this test scenario was as high as 99.5%. To further prove that the proposed model outperforms other models, a comparative analysis has been done with some of the machine learning algorithms. The proposed model has outperformed all the models generally and specifically when the model testing was done using an independent testing set.article:***class***class:

ChatGPT

**ChatGPT**

Based on the content of the provided article, it appears to fall into the category of **Diagnostic Assistance**. Here's why:

The article discusses the development of a convolutional neural network (CNN) architecture for the diagnosis of COVID-19 based on chest X-ray image classification. It addresses challenges related to the availability of a small-sized and imbalanced dataset with image-quality issues. The article describes preprocessing techniques, including dataset balancing, medical experts' image analysis, and data augmentation, to enhance the quality of the training dataset for the CNN model.

Diagnostic Assistance articles typically involve techniques, methods, or technologies aimed at improving diagnostic accuracy or reducing errors in a specific domain. In this case, the article focuses on the development of a CNN model for accurately diagnosing COVID-19 based on chest X-ray images. The experimental results indicate high accuracy levels for the proposed model, which demonstrates its capability in the application domain.

Therefore, you can classify this article as falling into the **Diagnostic Assistance** category based on the provided class definitions.

**Anonymous**

***article*** Advances in computational science offer a principled pipeline for predictive modeling of cardiovascular flows and aspire to provide a valuable tool for monitoring, diagnostics and surgical planning. Such models can be nowadays deployed on large patient-specific topologies of systemic arterial networks and return detailed predictions on flow patterns, wall shear stresses, and pulse wave propagation. However, their success heavily relies on tedious pre-processing and calibration procedures that typically induce a significant computational cost, thus hampering their clinical applicability. In this work we put forth a machine learning framework that enables the seamless synthesis of non-invasive in-vivo measurement techniques and computational flow dynamics models derived from first physical principles. We illustrate this new paradigm by showing how one-dimensional models of pulsatile flow can be used to constrain the output of deep neural networks such that their predictions satisfy the conservation of mass and momentum principles. Once trained on noisy and scattered clinical data of flow and wall displacement, these networks can return physically consistent predictions for velocity, pressure and wall displacement pulse wave propagation, all without the need to employ conventional simulators. A simple post-processing of these outputs can also provide a relatively cheap and effective way for estimating Windkessel model parameters that are required for the calibration of traditional computational models. The effectiveness of the proposed techniques is demonstrated through a series of prototype benchmarks, as well as a realistic clinical case involving in-vivo measurements near the aorta/carotid bifurcation of a healthy human subject. (C) 2019 Elsevier B.V. All rights reserved.article:***class***class:

ChatGPT

**ChatGPT**

Based on the content of the provided article, it appears to fall into the category of **Diagnostic Assistance**. Here's why:

The article discusses the development of a machine learning framework for predictive modeling of cardiovascular flows. It aims to provide a valuable tool for monitoring, diagnostics, and surgical planning in the context of cardiovascular health. The framework combines non-invasive in-vivo measurement techniques with computational flow dynamics models derived from physical principles.

Diagnostic Assistance articles typically involve techniques, methods, or technologies aimed at improving diagnostic accuracy or reducing errors in a specific domain. In this case, the article focuses on the development of a machine learning framework that can predict flow patterns, wall shear stresses, and pulse wave propagation in cardiovascular flows. It aims to provide physically consistent predictions using deep neural networks trained on clinical data.

Therefore, you can classify this article as falling into the **Diagnostic Assistance** category based on the provided class definitions.

**Anonymous**

***article*** Intelligent AI devices have become a common presence in the business landscape, offering a wide range of services, from the medical sector to the hospitality industry. From an organizational perspective, AI devices have several advantages, by performing certain tasks quicker and more accurately in comparison to humans while at the same time being more cost-efficient. However, in order to maintain the high standards of a brand, they have to be accepted by consumers and deliver socially adequate performance. Therefore, it is important to determine the characteristics of AI devices which make them accepted and trusted by consumers. Based on the Computers as Social Actors (CASA) Theory, we have researched on the role of psychological anthropomorphic characteristics, perceived empathy, and interaction quality in the acceptance of AI devices in the service industry. The results show that anthropomorphic characteristics alone do not influence acceptance and trust towards AI devices. However, both perceived empathy and interaction quality mediate the relation between anthropomorphic characteristics and acceptance. A human-like AI device has higher acceptance when it has the ability to show empathy and interaction in relation to the human consumer. This result reveals the importance of developing forms of strong intelligence and empathetic behaviour in service robots and AI devices.article:***class***class:

ChatGPT

**ChatGPT**

Based on the content of the provided article, it appears to fall into the category of **Prescriptive Guidance**. Here's why:

The article discusses the acceptance and trust of AI devices from a consumer perspective, particularly in the service industry. It explores the role of psychological anthropomorphic characteristics, perceived empathy, and interaction quality in influencing the acceptance of AI devices.

Prescriptive Guidance articles typically provide recommendations, insights, or discussions related to specific methods, techniques, or approaches within a particular domain. In this case, the article offers insights into the factors that contribute to the acceptance of AI devices, emphasizing the importance of perceived empathy and interaction quality in mediating the relationship between anthropomorphic characteristics and acceptance.

Therefore, you can classify this article as falling into the **Prescriptive Guidance** category based on the provided class definitions.

**Anonymous**

***article*** The production of blood cells is affected by leukemia, a type of bone marrow cancer or blood cancer. Deoxyribonucleic acid (DNA) is related to immature cells, particularly white cells, and is damaged in various ways in this disease. When a radiologist is involved in diagnosing acute leukemia cells, the diagnosis is time consuming and needs to provide better accuracy. For this purpose, many types of research have been conducted for the automatic diagnosis of acute leukemia. However, these studies have low detection speed and accuracy. Machine learning and artificial intelligence techniques are now playing an essential role in medical sciences, particularly in detecting and classifying leukemic cells. These methods assist doctors in detecting diseases earlier, reducing their workload and the possibility of errors. This research aims to design a deep learning model with a customized architecture for detecting acute leukemia using images of lymphocytes and monocytes. This study presents a novel dataset containing images of Acute Lymphoblastic Leukemia (ALL) and Acute Myeloid Leukemia (AML). The new dataset has been created with the assistance of various experts to help the scientific community in its efforts to incorporate machine learning techniques into medical research. Increasing the scale of the dataset is achieved with a Generative Adversarial Network (GAN). The proposed CNN model based on the Tversky loss function includes six convolution layers, four dense layers, and a Softmax activation function for the classification of acute leukemia images. The proposed model achieved a 99% accuracy rate in diagnosing acute leukemia types, including ALL and AML. Compared to previous research, the proposed network provides a promising performance in terms of speed and accuracy; and based on the results, the proposed model can be used to assist doctors and specialists in practical applications.article:***class***class:

ChatGPT

**ChatGPT**

Based on the content of the provided article, it appears to fall into the category of **Diagnostic Assistance**. Here's why:

The article focuses on the development of a deep learning model for the automatic diagnosis of acute leukemia using images of lymphocytes and monocytes. It discusses the use of machine learning and artificial intelligence techniques to assist doctors in detecting and classifying leukemic cells, aiming to improve accuracy and reduce the workload of medical professionals.

Diagnostic Assistance articles typically involve techniques, methods, or technologies aimed at improving diagnostic accuracy or reducing errors in a specific medical domain. In this case, the article is centered on the development of a deep learning model for diagnosing acute leukemia, and it highlights the high accuracy achieved by the proposed model in classifying different types of leukemia.

Therefore, you can classify this article as falling into the **Diagnostic Assistance** category based on the provided class definitions.

**Anonymous**

***article*** Deep learning applications have been thriving over the last decade in many different domains, including computer vision and natural language understanding. The drivers for the vibrant development of deep learning have been the availability of abundant data, breakthroughs of algorithms and the advancements in hardware. Despite the fact that complex industrial assets have been extensively monitored and large amounts of condition monitoring signals have been collected, the application of deep learning approaches for detecting, diagnosing and predicting faults of complex industrial assets has been limited. The current paper provides a thorough evaluation of the current developments, drivers, challenges, potential solutions and future research needs in the field of deep learning applied to Prognostics and Health Management (PHM) applications.article:***class***class:

ChatGPT

**ChatGPT**

Based on the content of the provided article, it appears to fall into the category of **Prescriptive Guidance**. Here's why:

The article discusses the application of deep learning approaches for detecting, diagnosing, and predicting faults in complex industrial assets, particularly in the context of Prognostics and Health Management (PHM) applications. It evaluates the current developments, challenges, potential solutions, and future research needs in this field.

Prescriptive Guidance articles typically provide recommendations, insights, or discussions related to specific methods, techniques, or approaches within a particular domain. In this case, the article offers insights into the adoption of deep learning in PHM applications for industrial asset management, highlighting the drivers and challenges associated with its implementation.

Therefore, you can classify this article as falling into the **Prescriptive Guidance** category based on the provided class definitions.

**Anonymous**

***article*** Medical image segmentation, which is a complex and fundamental step in medical image processing, can help doctors make more precise decisions on patient diagnosis. Although multi-threshold image segmentation is the most exceptionally fundamental image segmentation technology, it requires complex computing and tends to yield unsatisfactory segmentation results, leading to its limited applications. To solve this problem, in this study, an ensemble multi strategy-driven shuffled frog leaping algorithm with horizontal and vertical crossover search (HVSFLA) is designed for multi-threshold image segmentation. Specifically, a horizontal crossover search enables different frogs to exchange information and guarantee the compelling exploration of each frog. Meanwhile, a vertical crossover search can make frogs in stagnation continue to search actively. Therefore, a better balance between diversification and intensification can be ensured. To evaluate its performance, HVSFLA was compared with a range of state-of-the-art algorithms using CEC 2017 benchmark functions. Furthermore, the performance of HVSFLA was also proved on several Berkeley segmentation datasets 500 (BSD5500). Finally, the proposed algorithm was applied to breast invasive ductal carcinoma cases based on multi-threshold segmentation technique using a non-local means 2D histogram integrated with Kapur's entropy. The experimental results demonstrate that the proposed HVSFLA outperforms a broad array of similar competitors, and thus it has a great potential to be used for medical image segmentation.article:***class***class:

ChatGPT

**ChatGPT**

Based on the content of the provided article, it appears to fall into the category of **Diagnostic Assistance**. Here's why:

The article focuses on the development of an algorithm, specifically an ensemble multi strategy-driven shuffled frog leaping algorithm with horizontal and vertical crossover search (HVSFLA), for improving the accuracy of medical image segmentation. The goal is to assist doctors in making more precise decisions on patient diagnosis through improved image segmentation techniques.

Diagnostic Assistance articles typically involve techniques, methods, or technologies aimed at improving diagnostic accuracy or reducing errors in a specific medical domain. In this case, the article is centered on enhancing the accuracy of medical image segmentation, which is a fundamental step in medical image processing. The experimental results demonstrate the algorithm's potential for medical image segmentation.

Therefore, you can classify this article as falling into the **Diagnostic Assistance** category based on the provided class definitions.

**Anonymous**

***article*** Among other hospitalization causes and cases, the clinical emergency is a critical case and the data of the reporting patients are biased as well as poorly managed due to the chaotic situation. The world has faced chaos over the past year due to the frequent waves of COVID-19 and the resulting emergencies. The data banks, linked with the clinical emergencies require serious quantitative and qualitative analysis to drive interpretable conclusions for necessary future emergency measures and to develop explainable artificial intelligence tools. This important procedure involves the clear understanding of the data patterns and topologies, which is a great challenge for the multidimensional data sets. Mathematically, the topological mapping can resolve this problem by mapping higher-dimensional data to two-dimensional representation, based on the overall association. Proper data mining and pattern recognition can help in improving the rapid patients admission, in providing the medical resources timely and in proper patient administration. In this paper, the importance of self-organizing maps, to interpret the hospital data, particularly for the COVID-19 epidemic is discussed in detail. Important variables are identified with the aid of networks and mappings.article:***class***class:

ChatGPT

**ChatGPT**

Based on the content of the provided article, it appears to fall into the category of **Prescriptive Guidance**. Here's why:

The article discusses the challenges related to clinical emergency data management, especially during critical situations like the COVID-19 pandemic. It emphasizes the need for quantitative and qualitative analysis of the data and the development of explainable artificial intelligence tools to drive necessary future emergency measures.

Prescriptive Guidance articles typically provide recommendations, insights, or discussions related to specific methods, techniques, or approaches within a particular domain. In this case, the article highlights the importance of using self-organizing maps for interpreting hospital data, particularly in the context of the COVID-19 epidemic. It discusses the role of mathematical topological mapping in resolving challenges associated with multidimensional datasets.

Therefore, you can classify this article as falling into the **Prescriptive Guidance** category based on the provided class definitions.

**Anonymous**

***article*** Wearable sensors and social networking platforms play a key role in providing a new method to collect patient data for efficient healthcare monitoring. However, continuous patient monitoring using wearable sensors generates a large amount of healthcare data. In addition, the user-generated healthcare data on social networking sites come in large volumes and are unstructured. The existing healthcare monitoring systems are not efficient at extracting valuable information from sensors and social networking data, and they have difficulty analyzing it effectively. On top of that, the traditional machine learning approaches are not enough to process healthcare big data for abnormality prediction. Therefore, a novel healthcare monitoring framework based on the cloud environment and a big data analytics engine is proposed to precisely store and analyze healthcare data, and to improve the classification accuracy. The proposed big data analytics engine is based on data mining techniques, ontologies, and bidirectional long short-term memory (Bi-LSTM). Data mining techniques efficiently preprocess the healthcare data and reduce the dimensionality of the data. The proposed ontologies provide semantic knowledge about entities and aspects, and their relations in the domains of diabetes and blood pressure (BP). Bi-LSTM correctly classifies the healthcare data to predict drug side effects and abnormal conditions in patients. Also, the proposed system classifies the patients' health condition using their healthcare data related to diabetes, BP, mental health, and drug reviews. This framework is developed employing the Protege Web Ontology Language tool with Java. The results show that the proposed model precisely handles heterogeneous data and improves the accuracy of health condition classification and drug side effect predictions. (C) 2020 Elsevier B.V. All rights reserved.article:***class***class:

ChatGPT

**ChatGPT**

Based on the content of the provided article, it appears to fall into the category of **Diagnostic Assistance**. Here's why:

The article discusses the development of a healthcare monitoring framework that utilizes wearable sensors and social networking platforms to collect patient data for efficient healthcare monitoring. It addresses the challenges of handling large volumes of unstructured healthcare data generated by continuous patient monitoring.

Diagnostic Assistance articles typically involve techniques, methods, or technologies aimed at improving diagnostic accuracy or reducing errors in a specific medical domain. In this case, the article focuses on a novel healthcare monitoring framework that leverages big data analytics, data mining techniques, ontologies, and bidirectional long short-term memory (Bi-LSTM) to process and analyze healthcare data for abnormality prediction, drug side effect prediction, and health condition classification.

Therefore, you can classify this article as falling into the **Diagnostic Assistance** category based on the provided class definitions.

**Anonymous**

***article*** Coronavirus disease (COVID-19) is a pandemic disease, which has already caused thousands of causalities and infected several millions of people worldwide. Any technological tool enabling rapid screening of the COVID-19 infection with high accuracy can be crucially helpful to the healthcare professionals. The main clinical tool currently in use for the diagnosis of COVID-19 is the Reverse transcription polymerase chain reaction (RT-PCR), which is expensive, less-sensitive and requires specialized medical personnel. X-ray imaging is an easily accessible tool that can be an excellent alternative in the COVID-19 diagnosis. This research was taken to investigate the utility of artificial intelligence (AI) in the rapid and accurate detection of COVID-19 from chest X-ray images. The aim of this paper is to propose a robust technique for automatic detection of COVID-19 pneumonia from digital chest X-ray images applying pre-trained deep-learning algorithms while maximizing the detection accuracy. A public database was created by the authors combining several public databases and also by collecting images from recently published articles. The database contains a mixture of 423 COVID-19, 1485 viral pneumonia, and 1579 normal chest X-ray images. Transfer learning technique was used with the help of image augmentation to train and validate several pre-trained deep Convolutional Neural Networks (CNNs). The networks were trained to classify two different schemes: i) normal and COVID-19 pneumonia; ii) normal, viral and COVID-19 pneumonia with and without image augmentation. The classification accuracy, precision, sensitivity, and specificity for both the schemes were 99.7%, 99.7%, 99.7% and 99.55% and 97.9%, 97.95%, 97.9%, and 98.8%, respectively. The high accuracy of this computer-aided diagnostic tool can significantly improve the speed and accuracy of COVID-19 diagnosis. This would be extremely useful in this pandemic where disease burden and need for preventive measures are at odds with available resources.article:***class***class:

ChatGPT

**ChatGPT**

Based on the content of the provided article, it appears to fall into the category of **Diagnostic Assistance**. Here's why:

The article discusses the development of a robust technique for the automatic detection of COVID-19 pneumonia from digital chest X-ray images using artificial intelligence (AI). It aims to provide a computer-aided diagnostic tool that can rapidly and accurately detect COVID-19 from chest X-ray images, which can be crucial in the context of the COVID-19 pandemic.

Diagnostic Assistance articles typically involve techniques, methods, or technologies aimed at improving diagnostic accuracy or reducing errors in a specific medical domain. In this case, the article focuses on the application of AI, particularly deep Convolutional Neural Networks (CNNs), for the detection of COVID-19 from chest X-ray images. The high accuracy achieved by the proposed model indicates its potential to improve the speed and accuracy of COVID-19 diagnosis.

Therefore, you can classify this article as falling into the **Diagnostic Assistance** category based on the provided class definitions.

**Anonymous**

***article*** Smart health care is an important aspect of connected living. Health care is one of the basic pillars of human need, and smart health care is projected to produce several billion dollars in revenue in the near future. There are several components of smart health care, including the Internet of Things (IoT), the Internet of Medical Things (IoMT), medical sensors, artificial intelligence (AI), edge computing, cloud computing, and next-generation wireless communication technology. Many papers in the literature deal with smart health care or health care in general. Here, we present a comprehensive survey of IoT- and IoMT-based edge-intelligent smart health care, mainly focusing on journal articles published between 2014 and 2020. We survey this literature by answering several research areas on IoT and IoMT, AI, edge and cloud computing, security, and medical signals fusion. We also address current research challenges and offer some future research directions.article:***class***class:

ChatGPT

**ChatGPT**

Based on the content of the provided article, it appears to fall into the category of **Prescriptive Guidance**. Here's why:

The article provides a comprehensive survey of IoT- and IoMT-based edge-intelligent smart health care, focusing on various components, including the Internet of Things (IoT), the Internet of Medical Things (IoMT), medical sensors, artificial intelligence (AI), edge computing, cloud computing, and next-generation wireless communication technology. It reviews the literature and research areas related to smart health care and addresses current research challenges.

Prescriptive Guidance articles typically provide recommendations, insights, or discussions related to specific methods, techniques, or approaches within a particular domain. In this case, the article offers a comprehensive survey of smart health care technologies and research areas, highlighting the importance of IoT, IoMT, AI, edge computing, and other components in the context of connected health care.

Therefore, you can classify this article as falling into the **Prescriptive Guidance** category based on the provided class definitions.

**Anonymous**

***article*** In recent years, with the increase of computer computing power, Deep Learning has begun to be favored. Its learning of non-linear feature combinations has played a role that traditional machine learning cannot reach in almost every field. The application of Deep Learning has also driven the advancement of Factorization Machine (FM) in the field of recommendation systems, because Deep Learning and FM can learn high-order and low-order features combinations respectively, and FM's hidden vector system enables it to learn information from sparse data. The integration of them has attracted the attention of many scholars. They have researched many classic models such as Factorization-supported Neural Network (FNN), Product-based Neural Networks (PNN), Inner PNN (IPNN), Wide&Deep, Deep&Cross, DeepFM, etc. for the Click-Through-Rate (CTR) problem, and their performance is getting better and better. This kind of model is also suitable for agriculture, meteorology, disease prediction and other fields due to the above advantages. Based on the DeepFM model, we predicts the incidence of hepatitis in each sample in the structured disease prediction data of the 2020 Artificial Intelligence Challenge Preliminary Competition, and make minor improvements and parameter adjustments to DeepFM. Compared with other models, the improved DeepFM has excellent performance in AUC. This research can be applied to electronic medical records to reduce the workload of doctors and make doctors focus on the samples with higher predicted incidence rates. For some changing data, such as blood pressure, height, weight, cholesterol, etc., we can introduce the Internet of Medical Things (IoMT). IoMT's sensors can be used to conduct transmission to ensure that the disease can be predicted in time, just in case. After joining IoMT, a healthcare system is formed, which is superior in forecasting and time performance.article:***class***class:

ChatGPT

**ChatGPT**

Based on the content of the provided article, it appears to fall into the category of **Diagnostic Assistance**. Here's why:

The article discusses the application of Deep Learning, specifically the DeepFM model, in predicting the incidence of hepatitis using structured disease prediction data. It aims to use machine learning techniques to assist in disease prediction, which can help reduce the workload of doctors and focus their attention on samples with higher predicted incidence rates.

Diagnostic Assistance articles typically involve techniques, methods, or technologies aimed at improving diagnostic accuracy or reducing errors in a specific medical domain. In this case, the article focuses on the use of machine learning, specifically the DeepFM model, for disease prediction, which is a form of diagnostic assistance.

Therefore, you can classify this article as falling into the **Diagnostic Assistance** category based on the provided class definitions.

**Anonymous**

***article*** Cardiovascular disease is a substantial cause of mortality and morbidity in the world. In clinical data analytics, it is a great challenge to predict heart disease survivor. Data mining transforms huge amounts of raw data generated by the health industry into useful information that can help in making informed decisions. Various studies proved that significant features play a key role in improving performance of machine learning models. This study analyzes the heart failure survivors from the dataset of 299 patients admitted in hospital. The aim is to find significant features and effective data mining techniques that can boost the accuracy of cardiovascular patient's survivor prediction. To predict patient's survival, this study employs nine classification models: Decision Tree (DT), Adaptive boosting classifier (AdaBoost), Logistic Regression (LR), Stochastic Gradient classifier (SGD), Random Forest (RF), Gradient Boosting classifier (GBM), Extra Tree Classifier (ETC), Gaussian Naive Bayes classifier (G-NB) and Support Vector Machine (SVM). The imbalance class problem is handled by Synthetic Minority Oversampling Technique (SMOTE). Furthermore, machine learning models are trained on the highest ranked features selected by RF. The results are compared with those provided by machine learning algorithms using full set of features. Experimental results demonstrate that ETC outperforms other models and achieves 0.9262 accuracy value with SMOTE in prediction of heart patient's survival.article:***class***class:

ChatGPT

**ChatGPT**

Based on the content of the provided article, it appears to fall into the category of **Diagnostic Assistance**. Here's why:

The article focuses on the prediction of cardiovascular patient survival, which is a form of diagnostic assistance. It utilizes data mining techniques and machine learning models to analyze a dataset of 299 patients admitted to the hospital. The goal is to find significant features and effective data mining techniques that can improve the accuracy of predicting the survival of cardiovascular patients.

Diagnostic Assistance articles typically involve techniques, methods, or technologies aimed at improving diagnostic accuracy or reducing errors in a specific medical domain. In this case, the article focuses on the application of machine learning models, such as Decision Tree, AdaBoost, Logistic Regression, and others, to predict patient survival in the context of cardiovascular diseases.

Therefore, you can classify this article as falling into the **Diagnostic Assistance** category based on the provided class definitions.

**Anonymous**

***article*** The impact of the Internet of Things (IoT) on the advancement of the healthcare industry is immense. The ushering of the Medicine 4.0 has resulted in an increased effort to develop platforms, both at the hardware level as well as the underlying software level. This vision has led to the development of Healthcare IoT (H-IoT) systems. The basic enabling technologies include the communication systems between the sensing nodes and the processors; and the processing algorithms for generating an output from the data collected by the sensors. However, at present, these enabling technologies are also supported by several new technologies. The use of Artificial Intelligence (AI) has transformed the H-IoT systems at almost every level. The fog/edge paradigm is bringing the computing power close to the deployed network and hence mitigating many challenges in the process. While the big data allows handling an enormous amount of data. Additionally, the Software Defined Networks (SDNs) bring flexibility to the system while the blockchains are finding the most novel use cases in H-IoT systems. The Internet of Nano Things (IoNT) and Tactile Internet (TI) are driving the innovation in the H-IoT applications. This paper delves into the ways these technologies are transforming the H-IoT systems and also identifies the future course for improving the Quality of Service (QoS) using these new technologies.article:***class***class:

ChatGPT

**ChatGPT**

Based on the content of the provided article, it appears to fall into the category of **Prescriptive Guidance**. Here's why:

The article discusses the impact of various technologies, including the Internet of Things (IoT), Artificial Intelligence (AI), fog/edge computing, big data, Software Defined Networks (SDNs), blockchains, Internet of Nano Things (IoNT), and Tactile Internet (TI), on Healthcare IoT (H-IoT) systems. It explores how these technologies are transforming H-IoT systems and improving the Quality of Service (QoS).

Prescriptive Guidance articles typically provide recommendations, insights, or discussions related to specific methods, techniques, or approaches within a particular domain. In this case, the article offers a comprehensive overview of how various technologies are shaping the future of H-IoT systems and identifies the potential for improving QoS in healthcare applications.

Therefore, you can classify this article as falling into the **Prescriptive Guidance** category based on the provided class definitions.

**Anonymous**

***article*** Blood glucose (BG) prediction is essential to the success of glycemic control in type 1 diabetes (T1D) management. Empowered by the recent development of the Internet of Medical Things (IoMT), continuous glucose monitoring (CGM) and deep learning technologies have been demonstrated to achieve the state of the art in BG prediction. However, it is challenging to implement such algorithms in actual clinical settings to provide persistent decision support due to the high demand for computational resources, while smartphone-based implementations are limited by short battery life and require users to carry the device. In this work, we propose a new deep learning model using an attention-based evidential recurrent neural network and design an IoMT-enabled wearable device to implement the embedded model, which comprises a low-cost and low-power system on a chip to perform Bluetooth connectivity and edge computing for real-time BG prediction and predictive hypoglycemia detection. In addition, we developed a smartphone app to visualize BG trajectories and predictions, and desktop and cloud platforms to backup data and fine-tune models. The embedded model was evaluated on three clinical data sets including 47 T1D subjects. The proposed model achieved superior performance of root mean square error (RMSE), mean absolute error, and glucose-specific RMSE, and obtained the best accuracy for hypoglycemia detection when compared with a group of machine learning baseline methods. Moreover, we performed hardware-in-the-loop in silico trials with ten virtual T1D adults to test the whole IoMT system with predictive low-glucose management, which significantly reduced hypoglycemia and improved BG control.article:***class***class:

ChatGPT

**ChatGPT**

Based on the content of the provided article, it falls into the category of **Clinical Service Recommendation**. Here's why:

The article discusses the development of an Internet of Medical Things (IoMT)-enabled wearable device and a deep learning model for blood glucose (BG) prediction in the context of type 1 diabetes (T1D) management. It focuses on the practical implementation of these technologies in clinical settings to provide decision support for glycemic control.

Clinical Service Recommendation articles typically emphasize the need for specific technologies or approaches to be implemented in clinical settings to improve healthcare services or patient outcomes. In this case, the article describes the development of an embedded deep learning model and a wearable device to support real-time BG prediction and hypoglycemia detection for individuals with T1D.

Therefore, you can classify this article as falling into the **Clinical Service Recommendation** category based on the provided class definitions.

**Anonymous**

***article*** Along with the advancement of several emerging computing paradigms and technologies, such as cloud computing, mobile computing, artificial intelligence, and big data, Internet of Things (IoT) technologies have been applied in a variety of fields. In particular, the Internet of Healthcare Things (IoHT) is becoming increasingly important in human activity recognition (HAR) due to the rapid development of wearable and mobile devices. In this article, we focus on the deep-learning-enhanced HAR in IoHT environments. A semisupervised deep learning framework is designed and built for more accurate HAR, which efficiently uses and analyzes the weakly labeled sensor data to train the classifier learning model. To better solve the problem of the inadequately labeled sample, an intelligent autolabeling scheme based on deep Q-network (DQN) is developed with a newly designed distance-based reward rule which can improve the learning efficiency in IoT environments. A multisensor based data fusion mechanism is then developed to seamlessly integrate the on-body sensor data, context sensor data, and personal profile data together, and a long short-term memory (LSTM)-based classification method is proposed to identify fine-grained patterns according to the high-level features contextually extracted from the sequential motion data. Finally, experiments and evaluations are conducted to demonstrate the usefulness and effectiveness of the proposed method using real-world data.article:***class***class:

ChatGPT

**ChatGPT**

Based on the content of the provided article, it falls into the category of **Diagnostic Assistance**. Here's why:

The article discusses the application of Internet of Things (IoT) technologies, specifically in the context of the Internet of Healthcare Things (IoHT), for human activity recognition (HAR). It focuses on the development of a deep-learning-enhanced framework for HAR in IoHT environments.

Diagnostic Assistance articles typically revolve around the use of technology and data analysis techniques to assist in diagnosing or evaluating specific conditions or situations. In this case, the article is addressing the challenge of accurately recognizing human activities using sensor data from IoT devices.

Therefore, you can classify this article as falling into the **Diagnostic Assistance** category based on the provided class definitions.

**Anonymous**

***article*** Deep learning (DL) has driven innovation in the field of computational imaging. One of its bottlenecks is unavailable or insufficient training data. This article reviews an emerging paradigm, imaging physics-based data synthesis (IPADS), that can provide huge training data in biomedical magnetic resonance (MR) without or with few real data. Following the physical law of MR, IPADS generates signals from differential equations or analytical solution models, making learning more scalable and explainable and better protecting privacy. Key components of IPADS learning, including signal generation models, basic DL network structures, enhanced data generation, and learning methods, are discussed. Great IPADS potential has been demonstrated by representative applications in fast imaging, ultrafast signal reconstruction, and accurate parameter quantification. Finally, open questions and future work are discussed.article:***class***class:

ChatGPT

**ChatGPT**

Based on the content of the provided article, it falls into the category of **Medical Imaging**. Here's why:

The article discusses the use of deep learning (DL) in the field of computational imaging, specifically focusing on biomedical magnetic resonance (MR) imaging. It introduces an emerging paradigm called imaging physics-based data synthesis (IPADS), which aims to provide training data for DL models in cases where real data is unavailable or insufficient.

Medical Imaging articles typically revolve around the application of various imaging techniques in the field of healthcare and medicine. In this case, the article discusses the use of DL and IPADS for improving MR imaging, which is a common technique in medical diagnostics.

Therefore, you can classify this article as falling into the **Medical Imaging** category based on the provided class definitions.

**Anonymous**

there are only three classes

ChatGPT

**ChatGPT**

I apologize for the confusion earlier. Since there are only three available classes for categorizing articles, let's choose the most appropriate class based on the content provided in the article:

The article discusses the use of deep learning (DL) and imaging physics-based data synthesis (IPADS) in the context of biomedical magnetic resonance (MR) imaging. It focuses on improving MR imaging techniques, which are widely used in medical diagnostics. Given this content, the most suitable class among the three provided options is likely:

**Medical Imaging**

This classification accurately reflects the primary focus of the article, which is the application of advanced imaging techniques in the medical field.

**Anonymous**

the three classes are Prescriptive Guidance Clinical Service Recommendation Diagnostic Assistance

ChatGPT

**ChatGPT**

I apologize for the misunderstanding. Now that we have the correct classes, let's categorize the article into one of the three classes:

Based on the content provided, it seems that the most appropriate class for the article would be:

**Diagnostic Assistance**

The article discusses the use of deep learning and data synthesis in the context of medical imaging and healthcare diagnostics, which aligns with the "Diagnostic Assistance" category as it involves improving diagnostic processes using advanced technologies.

**Anonymous**

***article*** Physiological studies have shown that there are some differences in speech and facial activities between depressive and healthy individuals. Based on this fact, we propose a novel spatio-temporal attention (STA) network and a multimodal attention feature fusion (MAFF) strategy to obtain the multimodal representation of depression cues for predicting the individual depression level. Specifically, we first divide the speech amplitude spectrum/video into fixed-length segments and input these segments into the STA network, which not only integrates the spatial and temporal information through attention mechanism, but also emphasizes the audio/video frames related to depression detection. The audio/video segment-level feature is obtained from the output of the last full connection layer of the STA network. Second, this article employs the eigen evolution pooling method to summarize the changes of each dimension of the audio/video segment-level features to aggregate them into the audio/video level feature. Third, the multimodal representation with modal complementary information is generated using the MAFF and inputs into the support vector regression predictor for estimating depression severity. Experimental results on the AVEC2013 and AVEC2014 depression databases illustrate the effectiveness of our method.article:***class***class:

ChatGPT

**ChatGPT**

Based on the content provided, the most appropriate class for the article would be:

**Diagnostic Assistance**

The article discusses the use of a novel spatio-temporal attention (STA) network and a multimodal attention feature fusion (MAFF) strategy for predicting the individual depression level by analyzing speech and facial activities. This falls under the category of diagnostic assistance as it involves using technology to assist in diagnosing and assessing the severity of depression.

**Anonymous**

***article*** Pain is a complex phenomenon, involving sensory and emotional experience, that is often poorly understood, especially in infants, anesthetized patients, and others who cannot speak. Technology supporting pain assessment has the potential to help reduce suffering; however, advances are needed before it can be adopted clinically. This survey paper assesses the state of the art and provides guidance for researchers to help make such advances. First, we overview pain's biological mechanisms, physiological and behavioral responses, emotional components, as well as assessment methods commonly used in the clinic. Next, we discuss the challenges hampering the development and validation of pain recognition technology, and we survey existing datasets together with evaluation methods. We then present an overview of all automated pain recognition publications indexed in the Web of Science as well as from the proceedings of the major conferences on biomedical informatics and artificial intelligence, to provide understanding of the current advances that have been made. We highlight progress in both non-contact and contact-based approaches, tools using face, voice, physiology, and multi-modal information, the importance of context, and discuss challenges that exist, including identification of ground truth. Finally, we identify underexplored areas such as chronic pain and connections to treatments, and describe promising opportunities for continued advances.article:***class***class:

ChatGPT

**ChatGPT**

Based on the content provided, the most appropriate class for the article would be:

**Diagnostic Assistance**

The article discusses the development of technology and methods for the assessment and recognition of pain, which can assist in diagnosing and evaluating the condition of patients experiencing pain. This falls under the category of diagnostic assistance as it involves using technology to aid in diagnosing and assessing pain in various patient groups.

**Anonymous**

***article*** At present, computed tomography (CT) is widely used to assist disease diagnosis. Especially, computer aided diagnosis (CAD) based on artificial intelligence (AI) recently exhibits its importance in intelligent healthcare. However, it is a great challenge to establish an adequate labeled dataset for CT analysis assistance, due to the privacy and security issues. Therefore, this paper proposes a convolutional autoencoder deep learning framework to support unsupervised image features learning for lung nodule through unlabeled data, which only needs a small amount of labeled data for efficient feature learning. Through comprehensive experiments, it shows that the proposed scheme is superior to other approaches, which effectively solves the intrinsic labor-intensive problem during artificial image labeling. Moreover, it verifies that the proposed convolutional autoencoder approach can be extended for similarity measurement of lung nodules images. Especially, the features extracted through unsupervised learning are also applicable in other related scenarios.article:***class***class:

ChatGPT

**ChatGPT**

**Diagnostic Assistance**

This article falls under the category of "Diagnostic Assistance" as it discusses the use of a convolutional autoencoder deep learning framework to support unsupervised image features learning for lung nodule analysis in computed tomography (CT) scans. The goal is to aid in the diagnosis of lung nodules, which is a diagnostic application.

**Anonymous**

***article*** The importance of effective surgical wound care cannot never be underestimated. Poorly managing surgical wounds may cause many serious complications. Thus, it raises the necessity to develop a patient-friendly self-care system which can help both patients and medical professionals to ensure the state of the surgical wounds without any special medical equipment. In this paper, a surgical wound assessment system for self-care is proposed. The proposed system is designed to enable patients capture surgical wound images of themselves by using a mobile device and upload these images for analysis. Combining image-processing and machine-learning techniques, the proposed method is composed of four phases. First, images are segmented into superpixels where each superpixel contains the pixels in the similar color distribution. Second, these superpixels corresponding to the skin are identified and the area of connected skin superpixels is derived. Third, surgical wounds will be extracted from this area based on the observation of the texture difference between skin and wounds. Lastly, state and symptoms of surgical wound will be assessed. Extensive experimental results are conducted. With the proposed method, more than 90% state assessment results are correct and more than 91% symptom assessment results consistent with the actual diagnosis. Moreover, case studies are provided to show the advantage and limitation of this system. These results show that this system could perform well in the practical self-care scenario.article:***class***class:

ChatGPT

**ChatGPT**

**Diagnostic Assistance**

This article falls under the category of "Diagnostic Assistance" as it discusses the development of a surgical wound assessment system for self-care. The proposed system allows patients to capture surgical wound images using a mobile device and then uses image processing and machine learning techniques to assess the state and symptoms of the surgical wounds. The goal is to assist in the diagnostic process related to surgical wound care.

**Anonymous**

***article*** The accurate prediction of heart disease is essential to efficiently treating cardiac patients before a heart attack occurs. This goal can be achieved using an optimal machine learning model with rich healthcare data on heart diseases. Various systems based on machine learning have been presented recently to predict and diagnose heart disease. However, these systems cannot handle high-dimensional datasets due to the lack of a smart framework that can use different sources of data for heart disease prediction. In addition, the existing systems utilize conventional techniques to select features from a dataset and compute a general weight for them based on their significance. These methods have also failed to enhance the performance of heart disease diagnosis. In this paper, a smart healthcare system is proposed for heart disease prediction using ensemble deep learning and feature fusion approaches. First, the feature fusion method combines the extracted features from both sensor data and electronic medical records to generate valuable healthcare data. Second, the information gain technique eliminates irrelevant and redundant features, and selects the important ones, which decreases the computational burden and enhances the system performance. In addition, the conditional probability approach computes a specific feature weight for each class, which further improves system performance. Finally, the ensemble deep learning model is trained for heart disease prediction. The proposed system is evaluated with heart disease data and compared with traditional classifiers based on feature fusion, feature selection, and weighting techniques. The proposed system obtains accuracy of 98.5%, which is higher than existing systems. This result shows that our system is more effective for the prediction of heart disease, in comparison to other state-of-the-art methods.article:***class***class:

ChatGPT

**ChatGPT**

**Diagnostic Assistance**

This article belongs to the category of "Diagnostic Assistance" as it discusses the development of a smart healthcare system for heart disease prediction using ensemble deep learning and feature fusion approaches. The goal is to assist in the diagnostic process related to heart disease by predicting and diagnosing heart disease using healthcare data.

**Anonymous**

***article*** In recent years, intelligent automation in the healthcare sector becomes more familiar due to the integration of artificial intelligence (AI) techniques. Intelligent healthcare systems assist in making better decisions, which further enable the patient to provide improved medical services. At the same time, skin lesion is a deadly disease that affects people of all age groups. Skin lesion segmentation and classification play a vital part in the earlier and precise skin cancer diagnosis by intelligent systems. However, the automated diagnosis of skin lesions in dermoscopic images is challenging because of the problems such as artifacts (hair, gel bubble, ruler marker), blurry boundary, poor contrast, and variable sizes and shapes of the lesion images. This study develops intelligent multilevel thresholding with deep learning (IMLT-DL) based skin lesion segmentation and classification model using dermoscopic images to address these problems. Primarily, the presented IMLT-DL model incorporates the Top hat filtering and inpainting technique for the pre-processing of the dermoscopic images. In addition, the Mayfly Optimization (MFO) with multilevel Kapur's thresholding-based segmentation process is involved in determining the infected regions. Besides, an Inception v3 based feature extractor is applied to derive a valuable set of feature vectors. Finally, the classification process is carried out using a gradient boosting tree (GBT) model. The presented model's performance takes place against the International Skin Imaging Collaboration (ISIC) dataset, and the experimental outcomes are inspected in different evaluation measures. The resultant experimental values ensure that the proposed IMLT-DL model outperforms the existing methods by achieving higher accuracy of 0.992.article:***class***class:

ChatGPT

**ChatGPT**

**Diagnostic Assistance**

This article falls into the category of "Diagnostic Assistance" as it discusses the development of an intelligent multilevel thresholding with deep learning (IMLT-DL) based skin lesion segmentation and classification model using dermoscopic images. The goal is to assist in the diagnostic process related to skin lesions, specifically for skin cancer diagnosis, by automating the segmentation and classification of dermoscopic images of skin lesions.

**Anonymous**

***article*** Numerous studies have focused on making telemedicine smart through the Internet of Things (IoT) technology. These works span a wide range of research areas to enhance telemedicine architecture such as network communications, artificial intelligence methods and techniques, IoT wearable sensors and hardware devices, smartphones and cloud computing. Accordingly, several telemedicine applications covering various human diseases have presented their works from a specific perspective and resulted in confusion regarding the IoT characteristics. Although such applications are useful and necessary for improving telemedicine contexts related to monitoring, detection and diagnostics, deriving an overall picture of how IoT characteristics are currently integrated with the telemedicine architecture is difficult. Accordingly, this study complements the academic literature with a systematic review covering all main aspects of advances in IoT-based telemedicine architecture. This study also provides a state-of-the-art telemedicine classification taxonomy under IoT and reviews works in different fields in relation to that classification. To this end, this study checked the ScienceDirect, Institute of Electrical and Electronics Engineers (IEEE) Xplore, and Web of Science databases. A total of 2121 papers were collected from 2014 to July 2020. The retrieved articles were filtered according to the defined inclusion criteria. A final set of 141 articles were selected and classified into two categories, each followed by subcategories and sections. The first category includes an IoT-based telemedicine network that accounts for 24.11% (n = 34/141). The second category includes IoT-based telemedicine healthcare services and applications that account for 75.89% (n = 107/141). This multi-field systematic review has exposed new research opportunities, motivations, recommendations and challenges that need attention for the synergistic integration of interdisciplinary works. This extensive study also lists a set of open issues and provides innovative key solutions along with a systematic review. The classification of diseases under IoT-based telemedicine is divided into 14 groups. Furthermore, the crossover in our taxonomy is demonstrated. The lifecycle of the context of IoT-based telemedicine healthcare applications is mapped for the first time, including the procedure sequencing and definition for each context. We believe that this study is a useful guide for researchers and practitioners in providing direction and valuable information for future research. This study can also address the ambiguity in the trends in IoT-based telemedicine.article:***class***class:

ChatGPT

**ChatGPT**

**Diagnostic Assistance**

This article primarily falls into the category of "Diagnostic Assistance" as it discusses the integration of IoT (Internet of Things) technology into telemedicine architecture to enhance monitoring, detection, and diagnostics in various healthcare contexts. The focus is on using IoT characteristics to improve telemedicine applications related to human diseases.

**Anonymous**

***article*** Deep learning plays a vital role in classifying different arrhythmias using electrocardiography (ECG) data. Nevertheless, training deep learning models normally requires a large amount of data and can lead to privacy concerns. Unfortunately, a large amount of healthcare data cannot be easily collected from a single silo. Additionally, deep learning models are like black-box, with no explainability of the predicted results, which is often required in clinical healthcare. This limits the application of deep learning in real-world health systems. In this paper, to address the above-mentioned challenges, we design a novel end-to-end framework in a federated setting for ECG-based healthcare using explainable artificial intelligence (XAI) and deep convolutional neural networks (CNN). The federated setting is used to solve challenges such as data availability and privacy concerns. Furthermore, the proposed framework effectively classifies different arrhythmias using an autoencoder and a classifier, both based on a CNN. Additionally, we propose an XAI-based module on top of the proposed classifier for interpretability of the classification results, which helps clinical practitioners to interpret the predictions of the classifier and to make quick and reliable decisions. The proposed framework was trained and tested using the baseline Massachusetts Institute of Technology - Boston's Beth Israel Hospital (MIT-BIH) Arrhythmia database. The trained classifier outperformed existing work by achieving accuracy up to 94.5% and 98.9% for arrhythmia detection using noisy and clean data, respectively, with five-fold cross-validation. We also propose a new communication cost reduction method to reduce the communication costs and to enhance the privacy of users' data in the federated setting. While the proposed framework was tested and validated for ECG classification, it is general enough to be extended to many other healthcare applications. (C) 2021 Elsevier B.V. All rights reserved.article:***class***class:

ChatGPT

**ChatGPT**

**Diagnostic Assistance**

This article primarily falls into the category of "Diagnostic Assistance" as it discusses the use of deep learning and explainable artificial intelligence (XAI) to classify different arrhythmias using electrocardiography (ECG) data. The focus is on developing a federated framework for ECG-based healthcare that addresses challenges related to data privacy and interpretability of the classification results.

**Anonymous**

***article*** Background The SARS-CoV-2 variant B.1.1.7 was first identified in December, 2020, in England. We aimed to investigate whether increases in the proportion of infections with this variant are associated with differences in symptoms or disease course, reinfection rates, or transmissibility. Methods We did an ecological study to examine the association between the regional proportion of infections with the SARS-CoV-2 B.1.1.7 variant and reported symptoms, disease course, rates of reinfection, and transmissibility. Data on types and duration of symptoms were obtained from longitudinal reports from users of the COVID Symptom Study app who reported a positive test for COVID-19 between Sept 28 and Dec 27, 2020 (during which the prevalence of B.1.1.7 increased most notably in parts of the UK). From this dataset, we also estimated the frequency of possible reinfection, defined as the presence of two reported positive tests separated by more than 90 days with a period of reporting no symptoms for more than 7 days before the second positive test. The proportion of SARS-CoV-2 infections with the B.1.1.7 variant across the UK was estimated with use of genomic data from the COVID-19 Genomics UK Consortium and data from Public Health England on spike-gene target failure (a non-specific indicator of the B.1.1.7 variant) in community cases in England. We used linear regression to examine the association between reported symptoms and proportion of B.1.1.7. We assessed the Spearman correlation between the proportion of B.1.1.7 cases and number of reinfections over time, and between the number of positive tests and reinfections. We estimated incidence for B.1.1.7 and previous variants, and compared the effective reproduction number, R-t, for the two incidence estimates. Findings From Sept 28 to Dec 27, 2020, positive COVID-19 tests were reported by 36 920 COVID Symptom Study app users whose region was known and who reported as healthy on app sign-up. We found no changes in reported symptoms or disease duration associated with B.1.1.7. For the same period, possible reinfections were identified in 249 (0.7% [95% CI 0.6-0.8]) of 36 509 app users who reported a positive swab test before Oct 1, 2020, but there was no evidence that the frequency of reinfections was higher for the B.1.1.7 variant than for pre-existing variants. Reinfection occurrences were more positively correlated with the overall regional rise in cases (Spearman correlation 0.56-0.69 for South East, London, and East of England) than with the regional increase in the proportion of infections with the B.1.1.7 variant (Spearman correlation 0.38-0.56 in the same regions), suggesting B.1.1.7 does not substantially alter the risk of reinfection. We found a multiplicative increase in the R-t of B.1.1.7 by a factor of 1.35 (95% CI 1.02-1.69) relative to pre-existing variants. However, R-t fell below 1 during regional and national lockdowns, even in regions with high proportions of infections with the B.1.1.7 variant. Interpretation The lack of change in symptoms identified in this study indicates that existing testing and surveillance infrastructure do not need to change specifically for the B.1.1.7 variant. In addition, given that there was no apparent increase in the reinfection rate, vaccines are likely to remain effective against the B.1.1.7 variant.article:***class***class:

ChatGPT

**ChatGPT**

**Diagnostic Assistance**

This article does not directly fall into any of the three specified classes. Instead, it provides information related to the SARS-CoV-2 B.1.1.7 variant, investigating its impact on symptoms, disease course, reinfection rates, and transmissibility. While it doesn't fit perfectly into the specified classes, its focus is on understanding the characteristics of a specific COVID-19 variant, which could contribute to diagnostic assistance indirectly by informing healthcare professionals and researchers about the variant's behavior.

**Anonymous**

***article*** Background Data for front-line health-care workers and risk of COVID-19 are limited. We sought to assess risk of COVID-19 among front-line health-care workers compared with the general community and the effect of personal protective equipment (PPE) on risk. Methods We did a prospective, observational cohort study in the UK and the USA of the general community, including front-line health-care workers, using self-reported data from the COVID Symptom Study smartphone application (app) from March 24 (UK) and March 29 (USA) to April 23, 2020. Participants were voluntary users of the app and at first use provided information on demographic factors (including age, sex, race or ethnic background, height and weight, and occupation) and medical history, and subsequently reported any COVID-19 symptoms. We used Cox proportional hazards modelling to estimate multivariate-adjusted hazard ratios (HRs) of our primary outcome, which was a positive COVID-19 test. The COVID Symptom Study app is registered with ClinicalTrials.gov, NCT04331509. Findings Among 2 035 395 community individuals and 99 795 front-line health-care workers, we recorded 5545 incident reports of a positive COVID-19 test over 34 435 272 person-days. Compared with the general community, front-line health-care workers were at increased risk for reporting a positive COVID-19 test (adjusted HR 11.61, 95% CI 10.93-12.33). To account for differences in testing frequency between front-line health-care workers and the general community and possible selection bias, an inverse probability-weighted model was used to adjust for the likelihood of receiving a COVID-19 test (adjusted HR 3.40, 95% CI 3.37-3.43). Secondary and post-hoc analyses suggested adequacy of PPE, clinical setting, and ethnic background were also important factors. Interpretation In the UK and the USA, risk of reporting a positive test for COVID-19 was increased among front-line health-care workers. Health-care systems should ensure adequate availability of PPE and develop additional strategies to protect health-care workers from COVID-19, particularly those from Black, Asian, and minority ethnic backgrounds. Additional follow-up of these observational findings is needed. Copyright (C) 2020 The Author(s). Published by Elsevier Ltd.article:***class***class:

ChatGPT

**ChatGPT**

**Diagnostic Assistance**

This article provides insights into the risk of COVID-19 among front-line health-care workers compared to the general community and the impact of personal protective equipment (PPE) on this risk. It is relevant to diagnostic assistance as it assesses the risk of COVID-19 transmission among healthcare workers, which can help in understanding and managing the spread of the virus within healthcare settings.

**Anonymous**

***article*** Osteosarcoma is a malignant osteosarcoma that is extremely harmful to human health. Magnetic resonance imaging (MRI) technology is one of the commonly used methods for the imaging examination of osteosarcoma. Due to the large amount of osteosarcoma MRI image data and the complexity of detection, manual identification of osteosarcoma in MRI images is a time-consuming and labor-intensive task for doctors, and it is highly subjective, which can easily lead to missed and misdiagnosed problems. AI medical image-assisted diagnosis alleviates this problem. However, the brightness of MRI images and the multi-scale of osteosarcoma make existing studies still face great challenges in the identification of tumor boundaries. Based on this, this study proposed a prior guidance-based assisted segmentation method for MRI images of osteosarcoma, which is based on the few-shot technique for tumor segmentation and fine fitting. It not only solves the problem of multi-scale tumor localization, but also greatly improves the recognition accuracy of tumor boundaries. First, we preprocessed the MRI images using prior generation and normalization algorithms to reduce model performance degradation caused by irrelevant regions and high-level features. Then, we used a prior-guided feature abdominal muscle network to perform small-sample segmentation of tumors of different sizes based on features in the processed MRI images. Finally, using more than 80,000 MRI images from the Second Xiangya Hospital for experiments, the DOU value of the method proposed in this paper reached 0.945, which is at least 4.3% higher than other models in the experiment. We showed that our method specifically has higher prediction accuracy and lower resource consumption.article:***class***class:

ChatGPT

**ChatGPT**

**Diagnostic Assistance**

This article focuses on the use of artificial intelligence (AI) in medical image analysis, particularly for the segmentation of osteosarcoma in MRI images. The AI-based method aims to improve the accuracy and efficiency of tumor boundary recognition, which is essential for diagnostic assistance in identifying and assessing osteosarcoma in medical imaging.

**Anonymous**

***article*** Medical technological advancements have led to the creation of various large datasets with numerous attributes. The presence of redundant and irrelevant features in datasets negatively influences algorithms and leads to decreases in the performance of the algorithms. Using effective features in data mining and analyzing tasks such as classification can increase the accuracy of the results and relevant decisions made by decision-makers using them. This increase can become more acute when dealing with challenging, large-scale problems in medical applications. Nature-inspired metaheuristics show superior performance in finding optimal feature subsets in the literature. As a seminal attempt, a wrapper feature selection approach is presented on the basis of the newly proposed Aquila optimizer (AO) in this work. In this regard, the wrapper approach uses AO as a search algorithm in order to discover the most effective feature subset. S-shaped binary Aquila optimizer (SBAO) and V-shaped binary Aquila optimizer (VBAO) are two binary algorithms suggested for feature selection in medical datasets. Binary position vectors are generated utilizing S- and V-shaped transfer functions while the search space stays continuous. The suggested algorithms are compared to six recent binary optimization algorithms on seven benchmark medical datasets. In comparison to the comparative algorithms, the gained results demonstrate that using both proposed BAO variants can improve the classification accuracy on these medical datasets. The proposed algorithm is also tested on the real-dataset COVID-19. The findings testified that SBAO outperforms comparative algorithms regarding the least number of selected features with the highest accuracy.article:***class***class:

ChatGPT

**ChatGPT**

**Diagnostic Assistance**

This article discusses the importance of feature selection in medical data analysis to improve the performance of machine learning algorithms, especially in challenging, large-scale medical applications. It introduces two binary optimization algorithms, S-shaped binary Aquila optimizer (SBAO) and V-shaped binary Aquila optimizer (VBAO), for feature selection in medical datasets. The study demonstrates the effectiveness of these algorithms in improving classification accuracy on various benchmark medical datasets and highlights their potential application in real-world scenarios, such as COVID-19 diagnosis.

**Anonymous**

***article*** Medical image analysis methods have been applied to clinical scenarios of tumor diagnosis and treatment. Many studies have attempted to optimize the effectiveness of tumor MRI image segmentation by deep learning, but they do not consider the optimization of local details and the interaction of global semantic information. Second, although medical image pattern recognition can learn representative semantic features, it is challenging to ignore useless features in order to learn generalizable embeddings. Thus, a tumor-assisted segmentation method is proposed to detect tumor lesion regions and boundaries with complex shapes. Specifically, we introduce a denoising convolutional autoencoder (DCAE) for MRI image noise reduction. Furthermore, we design a novel tumor MRI image segmentation framework (NFSR-U-Net) based on class-correlation pattern aggregation, which first aggregates class-correlation patterns in MRI images to form a class-correlational representation. Then the relationship of similar class features is identified to closely correlate the dense representations of local features for classification, which is conducive to identifying image data with high heterogeneity. Meanwhile, the model uses a spatial attention mechanism and residual structure to extract effective information of the spatial dimension and enhance statistical information in MRI images, which bridges the semantic gap in skip connections. In the study, over 4000 MRI images from the Monash University Research Center for Artificial Intelligence are analyzed. The results show that the method achieves segmentation accuracy of up to 96% for tumor MRI images with low resource consumption.article:***class***class:

ChatGPT

**ChatGPT**

**Diagnostic Assistance**

This article presents a tumor-assisted segmentation method for MRI images, particularly for tumor lesion regions with complex shapes. The proposed method includes a denoising convolutional autoencoder (DCAE) for MRI image noise reduction and a novel tumor MRI image segmentation framework (NFSR-U-Net) that incorporates class-correlation pattern aggregation, spatial attention mechanisms, and residual structures. The study evaluates the method using over 4000 MRI images and achieves segmentation accuracy of up to 96% for tumor MRI images while maintaining low resource consumption.

**Anonymous**

***article*** Osteosarcoma is a malignant bone tumor that is extremely dangerous to human health. Not only does it require a large amount of work, it is also a complicated task to outline the lesion area in an image manually, using traditional methods. With the development of computer-aided diagnostic techniques, more and more researchers are focusing on automatic segmentation techniques for osteosarcoma analysis. However, existing methods ignore the size of osteosarcomas, making it difficult to identify and segment smaller tumors. This is very detrimental to the early diagnosis of osteosarcoma. Therefore, this paper proposes a Contextual Axial-Preserving Attention Network (CaPaN)-based MRI image-assisted segmentation method for osteosarcoma detection. Based on the use of Res2Net, a parallel decoder is added to aggregate high-level features which effectively combines the local and global features of osteosarcoma. In addition, channel feature pyramid (CFP) and axial attention (A-RA) mechanisms are used. A lightweight CFP can extract feature mapping and contextual information of different sizes. A-RA uses axial attention to distinguish tumor tissues by mining, which reduces computational costs and thus improves the generalization performance of the model. We conducted experiments using a real dataset provided by the Second Xiangya Affiliated Hospital and the results showed that our proposed method achieves better segmentation results than alternative models. In particular, our method shows significant advantages with respect to small target segmentation. Its precision is about 2% higher than the average values of other models. For the segmentation of small objects, the DSC value of CaPaN is 0.021 higher than that of the commonly used U-Net method.article:***class***class:

ChatGPT

**ChatGPT**

**Diagnostic Assistance**

This article presents a Contextual Axial-Preserving Attention Network (CaPaN)-based MRI image-assisted segmentation method for osteosarcoma detection. The proposed method utilizes Res2Net, a parallel decoder, channel feature pyramid (CFP), and axial attention (A-RA) mechanisms to effectively combine local and global features of osteosarcoma. The results of experiments using a real dataset show that the proposed method achieves better segmentation results than alternative models, especially for small target segmentation. The precision of the CaPaN method is approximately 2% higher than other models.

**Anonymous**

***article*** Recent technologies such as artificial intelligence, machine learning, and big data are essential for supporting healthcare monitoring systems, particularly for monitoring Monkeypox confirmed cases. Infected and uninfected cases around the world have contributed to a growing dataset, which is publicly available and can be used by artificial intelligence and machine learning to predict the confirmed cases of Monkeypox at an early stage. Motivated by this, we propose in this paper a new approach for accurate prediction of the Monkeypox confirmed cases based on an optimized Long Short-Term Memory (LSTM) deep network. To fine-tune the hyper-parameters of the LSTM-based deep network, we employed the Al-Biruni Earth Radius (BER) optimization algorithm; thus, the proposed approach is denoted by BER-LSTM. Experimental results show the effectiveness of the proposed approach when assessed using various evaluation criteria, such as Mean Bias Error, which is recorded as (0.06) using BER-LSTM. To prove the superiority of the proposed approach, six different machine learning models are included in the conducted experiments. In addition, four different optimization algorithms are considered for comparison purposes. The results of this comparison confirmed the superiority of the proposed approach. On the other hand, several statistical tests are applied to analyze the stability and significance of the proposed approach. These tests include one-way Analysis of Variance (ANOVA), Wilcoxon, and regression tests. The results of these tests emphasize the robustness, significance, and efficiency of the proposed approach.article:***class***class:

ChatGPT

**ChatGPT**

**Diagnostic Assistance**

This article proposes a new approach for predicting Monkeypox confirmed cases using an optimized Long Short-Term Memory (LSTM) deep network. The optimization of hyperparameters is performed using the Al-Biruni Earth Radius (BER) optimization algorithm. The proposed approach, denoted as BER-LSTM, is found to be effective based on various evaluation criteria, including a Mean Bias Error of 0.06. Comparative experiments with six different machine learning models and four optimization algorithms confirm the superiority of the proposed approach. Statistical tests, including ANOVA, Wilcoxon, and regression tests, further emphasize the robustness, significance, and efficiency of the proposed method.

**Anonymous**

***article*** Artificial intelligence (AI) researchers and radiologists have recently reported AI systems that accurately detect COVID-19 in chest radiographs. However, the robustness of these systems remains unclear. Using state-of-the-art techniques in explainable AI, we demonstrate that recent deep learning systems to detect COVID-19 from chest radiographs rely on confounding factors rather than medical pathology, creating an alarming situation in which the systems appear accurate, but fail when tested in new hospitals. We observe that the approach to obtain training data for these AI systems introduces a nearly ideal scenario for AI to learn these spurious 'shortcuts'. Because this approach to data collection has also been used to obtain training data for the detection of COVID-19 in computed tomography scans and for medical imaging tasks related to other diseases, our study reveals a far-reaching problem in medical-imaging AI. In addition, we show that evaluation of a model on external data is insufficient to ensure AI systems rely on medically relevant pathology, because the undesired 'shortcuts' learned by AI systems may not impair performance in new hospitals. These findings demonstrate that explainable AI should be seen as a prerequisite to clinical deployment of machine-learning healthcare models. The urgency of the developing COVID-19 epidemic has led to a large number of novel diagnostic approaches, many of which use machine learning. DeGrave and colleagues use explainable AI techniques to analyse a selection of these approaches and find that the methods frequently learn to identify features unrelated to the actual disease.article:***class***class:

ChatGPT

**ChatGPT**

**Diagnostic Assistance**

This article discusses the use of artificial intelligence (AI) systems to detect COVID-19 in chest radiographs. It raises concerns about the robustness of these systems, highlighting that they may rely on confounding factors rather than medical pathology, leading to potential issues when tested in new hospitals. The study emphasizes the importance of explainable AI and the need for rigorous evaluation to ensure AI systems are clinically reliable.

**Anonymous**

***article*** Using large, multi-national datasets for high-performance medical imaging AI systems requires innovation in privacy-preserving machine learning so models can train on sensitive data without requiring data transfer. Here we present PriMIA (Privacy-preserving Medical Image Analysis), a free, open-source software framework for differentially private, securely aggregated federated learning and encrypted inference on medical imaging data. We test PriMIA using a real-life case study in which an expert-level deep convolutional neural network classifies paediatric chest X-rays; the resulting model's classification performance is on par with locally, non-securely trained models. We theoretically and empirically evaluate our framework's performance and privacy guarantees, and demonstrate that the protections provided prevent the reconstruction of usable data by a gradient-based model inversion attack. Finally, we successfully employ the trained model in an end-to-end encrypted remote inference scenario using secure multi-party computation to prevent the disclosure of the data and the model. Gaining access to medical data to train AI applications can present problems due to patient privacy or proprietary interests. A way forward can be privacy-preserving federated learning schemes. Kaissis, Ziller and colleagues demonstrate here their open source framework for privacy-preserving medical image analysis in a remote inference scenario.article:***class***class:

ChatGPT

**ChatGPT**

**Prescriptive Guidance**

This article introduces PriMIA, a privacy-preserving software framework designed for medical imaging data analysis. It focuses on enabling differential privacy, securely aggregated federated learning, and encrypted inference for medical imaging datasets. The framework is tested using a case study involving the classification of pediatric chest X-rays, demonstrating its effectiveness in preserving privacy while maintaining classification performance. The article addresses the challenges of accessing medical data for AI applications and provides a solution for privacy-preserving federated learning.

**Anonymous**

***article*** The sudden increase in COVID-19 cases is putting high pressure on healthcare services worldwide. At this stage, fast, accurate and early clinical assessment of the disease severity is vital. To support decision making and logistical planning in healthcare systems, this study leverages a database of blood samples from 485 infected patients in the region of Wuhan, China, to identify crucial predictive biomarkers of disease mortality. For this purpose, machine learning tools selected three biomarkers that predict the mortality of individual patients more than 10 days in advance with more than 90% accuracy: lactic dehydrogenase (LDH), lymphocyte and high-sensitivity C-reactive protein (hs-CRP). In particular, relatively high levels of LDH alone seem to play a crucial role in distinguishing the vast majority of cases that require immediate medical attention. This finding is consistent with current medical knowledge that high LDH levels are associated with tissue breakdown occurring in various diseases, including pulmonary disorders such as pneumonia. Overall, this Article suggests a simple and operable decision rule to quickly predict patients at the highest risk, allowing them to be prioritized and potentially reducing the mortality rate. Early and accurate clinical assessment of disease severity in COVID-19 patients is essential for planning the allocation of scarce hospital resources. An explainable machine learning tool trained on blood sample data from 485 patients from Wuhan selected three biomarkers for predicting mortality of individual patients with high accuracy.article:***class***class:

ChatGPT

**ChatGPT**

**Clinical Service Recommendation**

This article discusses the importance of fast and accurate clinical assessment of COVID-19 disease severity to support decision-making and logistical planning in healthcare systems. It leverages a database of blood samples from infected patients in Wuhan, China, to identify predictive biomarkers of disease mortality. Machine learning tools identified three biomarkers, including lactic dehydrogenase (LDH), lymphocyte, and high-sensitivity C-reactive protein (hs-CRP), that can predict mortality with over 90% accuracy more than 10 days in advance. The article suggests that relatively high levels of LDH can play a crucial role in identifying cases requiring immediate medical attention, potentially reducing the mortality rate.

**Anonymous**

***article*** In a short period of time, many areas of science have made a sharp transition towards data-dependent methods. In some cases, this process has been enabled by simultaneous advances in data acquisition and the development of networked system technologies. This new situation is particularly clear in the life sciences, where data overabundance has sparked a flurry of new methodologies for data management and analysis. This can be seen as a perfect scenario for the use of machine learning and computational intelligence techniques to address problems in which more traditional data analysis approaches might struggle. But, this scenario also poses some serious challenges. One of them is model interpretability and explainability, especially for complex nonlinear models. In some areas such as medicine and health care, not addressing such challenge might seriously limit the chances of adoption, in real practice, of computer-based systems that rely on machine learning and computational intelligence methods for data analysis. In this paper, we reflect on recent investigations about the interpretability and explainability of machine learning methods and discuss their impact on medicine and health care. We pay specific attention to one of the ways in which interpretability and explainability in this context can be addressed, which is through data and model visualization. We argue that, beyond improving model interpretability as a goal in itself, we need to integrate the medical experts in the design of data analysis interpretation strategies. Otherwise, machine learning is unlikely to become a part of routine clinical and health care practice.article:***class***class:

ChatGPT

**ChatGPT**

**Clinical Service Recommendation**

This article discusses the transition towards data-dependent methods in various areas of science, particularly in the life sciences, driven by advances in data acquisition and networked system technologies. It emphasizes the importance of using machine learning and computational intelligence techniques to address data-related challenges in these fields. However, the article also highlights the challenge of model interpretability and explainability, especially in areas like medicine and healthcare, where the adoption of machine learning methods depends on addressing these challenges. The paper reflects on recent investigations into the interpretability and explainability of machine learning methods and emphasizes the role of data and model visualization in addressing these issues. It suggests that involving medical experts in designing data analysis interpretation strategies is crucial for integrating machine learning into routine clinical and healthcare practice.

**Anonymous**

***article*** Data-driven machine learning (ML) has emerged as a promising approach for building accurate and robust statistical models from medical data, which is collected in huge volumes by modern healthcare systems. Existing medical data is not fully exploited by ML primarily because it sits in data silos and privacy concerns restrict access to this data. However, without access to sufficient data, ML will be prevented from reaching its full potential and, ultimately, from making the transition from research to clinical practice. This paper considers key factors contributing to this issue, explores how federated learning (FL) may provide a solution for the future of digital health and highlights the challenges and considerations that need to be addressed.article:***class***class:

ChatGPT

**ChatGPT**

**Prescriptive Guidance**

This article discusses the challenges related to the use of data-driven machine learning (ML) in healthcare, particularly in building accurate and robust statistical models from medical data. It points out that existing medical data is often underutilized due to data silos and privacy concerns, which limit access to valuable healthcare data. The article suggests that federated learning (FL) could offer a solution to this problem by enabling collaborative model training across decentralized data sources while preserving data privacy. It highlights the challenges and considerations that need to be addressed for the successful implementation of FL in digital health.

**Anonymous**

***article*** The era of artificial intelligence and internet of things is rapidly developed by recent advances in wearable electronics. Gait reveals sensory information in daily life containing personal information, regarding identification and healthcare. Current wearable electronics of gait analysis are mainly limited by high fabrication cost, operation energy consumption, or inferior analysis methods, which barely involve machine learning or implement nonoptimal models that require massive datasets for training. Herein, we developed low-cost triboelectric intelligent socks for harvesting waste energy from low-frequency body motions to transmit wireless sensory data. The sock equipped with self-powered functionality also can be used as wearable sensors to deliver information, regarding the identity, health status, and activity of the users. To further address the issue of ineffective analysis methods, an optimized deep learning model with an end-to-end structure on the socks signals for the gait analysis is proposed, which produces a 93.54% identification accuracy of 13 participants and detects five different human activities with 96.67% accuracy. Toward practical application, we map the physical signals collected through the socks in the virtual space to establish a digital human system for sports monitoring, healthcare, identification, and future smart home applications.article:***class***class:

ChatGPT

**ChatGPT**

**Article Summary:**

This article discusses the development of low-cost triboelectric intelligent socks that can harvest energy from body motions and transmit wireless sensory data. These socks serve as wearable sensors for gait analysis, allowing for the identification, health status, and activity tracking of users. The article also introduces an optimized deep learning model to analyze the signals from the socks, achieving high accuracy in identification and activity detection. The socks have potential applications in sports monitoring, healthcare, identification, and smart home systems.

**Anonymous**

***article*** The era of artificial intelligence and internet of things is rapidly developed by recent advances in wearable electronics. Gait reveals sensory information in daily life containing personal information, regarding identification and healthcare. Current wearable electronics of gait analysis are mainly limited by high fabrication cost, operation energy consumption, or inferior analysis methods, which barely involve machine learning or implement nonoptimal models that require massive datasets for training. Herein, we developed low-cost triboelectric intelligent socks for harvesting waste energy from low-frequency body motions to transmit wireless sensory data. The sock equipped with self-powered functionality also can be used as wearable sensors to deliver information, regarding the identity, health status, and activity of the users. To further address the issue of ineffective analysis methods, an optimized deep learning model with an end-to-end structure on the socks signals for the gait analysis is proposed, which produces a 93.54% identification accuracy of 13 participants and detects five different human activities with 96.67% accuracy. Toward practical application, we map the physical signals collected through the socks in the virtual space to establish a digital human system for sports monitoring, healthcare, identification, and future smart home applications.article:***class***class:

ChatGPT

**ChatGPT**

**Article Summary:**

This article discusses the development of low-cost triboelectric intelligent socks that can harvest energy from low-frequency body motions and transmit wireless sensory data. These socks serve as wearable sensors for gait analysis, providing information about the user's identity, health status, and activity. To improve the analysis of gait data, the article introduces an optimized deep learning model with an end-to-end structure, achieving high accuracy in participant identification and activity detection. The socks have potential applications in sports monitoring, healthcare, identification, and smart home systems.

**Anonymous**

no choose from the three classes

ChatGPT

**ChatGPT**

I apologize for the oversight. Please provide the specific classes or categories you would like to choose from, and I'll be happy to assist you in categorizing the article accordingly.

**Anonymous**

Diagnostic Assistance: The article discusses the evaluation of seven automated AI-based diabetic retinopathy (DR) screening algorithms against human graders. The algorithms analyze retinal imaging data to classify images as referable DR or not. This falls under the category of diagnostic assistance in improving diagnostic accuracy or reducing errors. Prescriptive Guidance: The article mentions that the algorithms' sensitivities varied, with some achieving higher sensitivities and comparable specificity. This information can be considered as prescriptive guidance because it provides recommendations on the performance of the algorithms in detecting DR and their comparative analysis against human graders. Clinical Service Recommendation: The article emphasizes the need for rigorous testing of all DR screening algorithms on real-world data before clinical implementation. This recommendation aims to automate the clinical service of DR screening, minimizing doctor involvement in the process.

ChatGPT

**ChatGPT**

Thank you for providing the categories. Here's the classification of the article based on the information you've provided:

- **Diagnostic Assistance:** The article primarily falls under the category of "Diagnostic Assistance" as it discusses the evaluation of AI-based diabetic retinopathy (DR) screening algorithms, which aim to assist in diagnosing DR and improving diagnostic accuracy.
- **Prescriptive Guidance:** The article also contains elements of "Prescriptive Guidance" as it provides recommendations on the performance of the algorithms and their comparative analysis against human graders. This guidance can help in making informed decisions regarding the use of these algorithms.
- **Clinical Service Recommendation:** Additionally, the article touches upon "Clinical Service Recommendation" by emphasizing the need for rigorous testing of DR screening algorithms on real-world data before clinical implementation. This recommendation aims to streamline and improve the clinical service of DR screening.

If you have any more articles or need further assistance, please feel free to let me know.

**Anonymous**

***article*** The era of artificial intelligence and internet of things is rapidly developed by recent advances in wearable electronics. Gait reveals sensory information in daily life containing personal information, regarding identification and healthcare. Current wearable electronics of gait analysis are mainly limited by high fabrication cost, operation energy consumption, or inferior analysis methods, which barely involve machine learning or implement nonoptimal models that require massive datasets for training. Herein, we developed low-cost triboelectric intelligent socks for harvesting waste energy from low-frequency body motions to transmit wireless sensory data. The sock equipped with self-powered functionality also can be used as wearable sensors to deliver information, regarding the identity, health status, and activity of the users. To further address the issue of ineffective analysis methods, an optimized deep learning model with an end-to-end structure on the socks signals for the gait analysis is proposed, which produces a 93.54% identification accuracy of 13 participants and detects five different human activities with 96.67% accuracy. Toward practical application, we map the physical signals collected through the socks in the virtual space to establish a digital human system for sports monitoring, healthcare, identification, and future smart home applications.article:***class***class:

ChatGPT

**ChatGPT**

This article can be classified into the following categories:

- **Wearable Technology**: The article discusses the development of wearable electronics in the form of intelligent socks that utilize triboelectric technology to harvest energy from body motions. This technology is part of the broader field of wearable electronics, which falls under the category of "Wearable Technology."
- **Healthcare and Monitoring**: The article mentions that the intelligent socks can deliver information related to the users' health status and activity. This aspect is related to healthcare monitoring, making it relevant to the "Healthcare and Monitoring" category.
- **Identification and Authentication**: The article also mentions that the socks can provide information regarding the identity of the users. This aspect relates to identification and authentication technologies.
- **Machine Learning and Deep Learning**: The article discusses the use of an optimized deep learning model for gait analysis based on signals from the socks. This falls under the category of "Machine Learning and Deep Learning."
- **Internet of Things (IoT)**: The article mentions that the development of these intelligent socks is part of the broader advancements in the Internet of Things (IoT) and how they can be applied to various applications, including sports monitoring and smart home applications.

Overall, the article encompasses multiple categories related to wearable technology, healthcare, identification, machine learning, and IoT.

**Anonymous**

choose from the three classes: Diagnostic Assistance: The article primarily falls under the category of "Diagnostic Assistance" as it discusses the evaluation of AI-based diabetic retinopathy (DR) screening algorithms, which aim to assist in diagnosing DR and improving diagnostic accuracy. Prescriptive Guidance: The article also contains elements of "Prescriptive Guidance" as it provides recommendations on the performance of the algorithms and their comparative analysis against human graders. This guidance can help in making informed decisions regarding the use of these algorithms. Clinical Service Recommendation: Additionally, the article touches upon "Clinical Service Recommendation" by emphasizing the need for rigorous testing of DR screening algorithms on real-world data before clinical implementation. This recommendation aims to streamline and improve the clinical service of DR screening.

ChatGPT

**ChatGPT**

Based on the provided information, the article can be classified into the following category:

**Diagnostic Assistance**: The article primarily falls under the category of "Diagnostic Assistance" as it discusses the evaluation of AI-based diabetic retinopathy (DR) screening algorithms, which aim to assist in diagnosing DR and improving diagnostic accuracy.

**Anonymous**

***article*** The Coronavirus Disease 2019 (COVID-19) pandemic continues to have a devastating effect on the health and well-being of the global population. A critical step in the fight against COVID-19 is effective screening of infected patients, with one of the key screening approaches being radiology examination using chest radiography. It was found in early studies that patients present abnormalities in chest radiography images that are characteristic of those infected with COVID-19. Motivated by this and inspired by the open source efforts of the research community, in this study we introduce COVID-Net, a deep convolutional neural network design tailored for the detection of COVID-19 cases from chest X-ray (CXR) images that is open source and available to the general public. To the best of the authors' knowledge, COVID-Net is one of the first open source network designs for COVID-19 detection from CXR images at the time of initial release. We also introduce COVIDx, an open access benchmark dataset that we generated comprising of 13,975 CXR images across 13,870 patient patient cases, with the largest number of publicly available COVID-19 positive cases to the best of the authors' knowledge. Furthermore, we investigate how COVID-Net makes predictions using an explainability method in an attempt to not only gain deeper insights into critical factors associated with COVID cases, which can aid clinicians in improved screening, but also audit COVID-Net in a responsible and transparent manner to validate that it is making decisions based on relevant information from the CXR images. By no means a production-ready solution, the hope is that the open access COVID-Net, along with the description on constructing the open source COVIDx dataset, will be leveraged and build upon by both researchers and citizen data scientists alike to accelerate the development of highly accurate yet practical deep learning solutions for detecting COVID-19 cases and accelerate treatment of those who need it the most.article:***class***class:

ChatGPT

**ChatGPT**

**Diagnostic Assistance**: This article falls under the category of "Diagnostic Assistance" as it discusses the development of COVID-Net, a deep convolutional neural network designed for the detection of COVID-19 cases from chest X-ray (CXR) images. It aims to assist in diagnosing COVID-19 by analyzing radiology images for abnormalities characteristic of the disease. Additionally, the article mentions the use of explainability methods to gain insights into critical factors associated with COVID cases, which can aid clinicians in improved screening.

**Anonymous**

***article*** Deep learning has shown tremendous potential in many real-life applications in different domains. One of these potentials is object detection. Recent object detection which is based on deep learning models has achieved promising results concerning the finding of an object in images. The objective of this paper is to annotate and localize the medical face mask objects in real-life images. Wearing a medical face mask in public areas, protect people from COVID-19 transmission among them. The proposed model consists of two components. The first component is designed for the feature extraction process based on the ResNet-50 deep transfer learning model. While the second component is designed for the detection of medical face masks based on YOLO v2. Two medical face masks datasets have been combined in one dataset to be investigated through this research. To improve the object detection process, mean IoU has been used to estimate the best number of anchor boxes. The achieved results concluded that the adam optimizer achieved the highest average precision percentage of 81% as a detector. Finally, a comparative result with related work has been presented at the end of the research. The proposed detector achieved higher accuracy and precision than the related work.article:***class***class:

ChatGPT

**ChatGPT**

**Diagnostic Assistance**: This article falls under the category of "Diagnostic Assistance" as it discusses the development of a deep learning model for annotating and localizing medical face mask objects in real-life images. The primary objective is to assist in identifying whether individuals are wearing medical face masks in public areas to protect against COVID-19 transmission. This technology aids in diagnosing whether people are following recommended safety measures related to mask-wearing.

**Anonymous**

***article*** The coronavirus (COVID-19) pandemic is putting healthcare systems across the world under unprecedented and increasing pressure according to the World Health Organization (WHO). With the advances in computer algorithms and especially Artificial Intelligence, the detection of this type of virus in the early stages will help in fast recovery and help in releasing the pressure off healthcare systems. In this paper, a GAN with deep transfer learning for coronavirus detection in chest X-ray images is presented. The lack of datasets for COVID-19 especially in chest X-rays images is the main motivation of this scientific study. The main idea is to collect all the possible images for COVID-19 that exists until the writing of this research and use the GAN network to generate more images to help in the detection of this virus from the available X-rays images with the highest accuracy possible. The dataset used in this research was collected from different sources and it is available for researchers to download and use it. The number of images in the collected dataset is 307 images for four different types of classes. The classes are the COVID-19, normal, pneumonia bacterial, and pneumonia virus. Three deep transfer models are selected in this research for investigation. The models are the Alexnet, Googlenet, and Restnet18. Those models are selected for investigation through this research as it contains a small number of layers on their architectures, this will result in reducing the complexity, the consumed memory and the execution time for the proposed model. Three case scenarios are tested through the paper, the first scenario includes four classes from the dataset, while the second scenario includes 3 classes and the third scenario includes two classes. All the scenarios include the COVID-19 class as it is the main target of this research to be detected. In the first scenario, the Googlenet is selected to be the main deep transfer model as it achieves 80.6% in testing accuracy. In the second scenario, the Alexnet is selected to be the main deep transfer model as it achieves 85.2% in testing accuracy, while in the third scenario which includes two classes (COVID-19, and normal), Googlenet is selected to be the main deep transfer model as it achieves 100% in testing accuracy and 99.9% in the validation accuracy. All the performance measurement strengthens the obtained results through the research.article:***class***class:

ChatGPT

**ChatGPT**

**Diagnostic Assistance**: This article primarily falls under the category of "Diagnostic Assistance" as it discusses the development of a deep learning model for coronavirus (COVID-19) detection in chest X-ray images. The objective is to assist in the early detection of COVID-19 in patients through the analysis of medical images, thereby aiding in the diagnostic process.

**Anonymous**

***article*** Artificial intelligence (AI) in healthcare is becoming increasingly important, given its potential to generate and analyse healthcare data to improve patient care and reduce costs and clinical risk while enhancing administrative processes within organisations. AI can introduce new sources of growth, change how people work and improve the effectiveness of their work. Consequently, implementing AI systems in healthcare can enable the optimisation of healthcare resources, facilitate a better patient experience, improve population health, reduce per capita costs, and improve the satisfaction of health professionals. Nowadays, most studies have focused on the potential benefits and barriers to implementing AI in healthcare, while only a few have explained the rational decision-making process for deploying new technologies in the healthcare system. In this study, we aim to fill this gap by investigating how AI supports the effective and efficient management of the healthcare system by examining the Humber River Hospital in Toronto using the case study methodology. To achieve the desired benefits from the process of implementing technology in healthcare, our key findings show that hospitals need to undergo a business transformation that exploits technology. Finally, we conclude that only effective knowledge of tech-nology will enable hospitals to effectively become technological and digital.article:***class***class:

ChatGPT

**ChatGPT**

**Operational Efficiency Enhancement**: This article primarily falls under the category of "Operational Efficiency Enhancement" as it discusses the implementation of artificial intelligence (AI) systems in healthcare to optimize healthcare resources, improve administrative processes, and enhance the overall efficiency of healthcare organizations. The focus is on how AI can support effective and efficient management within the healthcare system, with a case study conducted at Humber River Hospital in Toronto.

**Anonymous**

choose from the following three classes - Diagnostic Assistance: The article primarily falls under the category of "Diagnostic Assistance" as it discusses the evaluation of AI-based diabetic retinopathy (DR) screening algorithms, which aim to assist in diagnosing DR and improving diagnostic accuracy. Prescriptive Guidance: The article also contains elements of "Prescriptive Guidance" as it provides recommendations on the performance of the algorithms and their comparative analysis against human graders. This guidance can help in making informed decisions regarding the use of these algorithms. Clinical Service Recommendation: Additionally, the article touches upon "Clinical Service Recommendation" by emphasizing the need for rigorous testing of DR screening algorithms on real-world data before clinical implementation. This recommendation aims to streamline and improve the clinical service of DR screening.

ChatGPT

**ChatGPT**

I apologize for the confusion, but I don't see the specific article you're referring to in your recent message. If you could provide the title or key content of the article, I'd be happy to help you classify it into one of the three classes you mentioned: Diagnostic Assistance, Prescriptive Guidance, or Clinical Service Recommendation.

**Anonymous**

***article*** Artificial intelligence (AI) in healthcare is becoming increasingly important, given its potential to generate and analyse healthcare data to improve patient care and reduce costs and clinical risk while enhancing administrative processes within organisations. AI can introduce new sources of growth, change how people work and improve the effectiveness of their work. Consequently, implementing AI systems in healthcare can enable the optimisation of healthcare resources, facilitate a better patient experience, improve population health, reduce per capita costs, and improve the satisfaction of health professionals. Nowadays, most studies have focused on the potential benefits and barriers to implementing AI in healthcare, while only a few have explained the rational decision-making process for deploying new technologies in the healthcare system. In this study, we aim to fill this gap by investigating how AI supports the effective and efficient management of the healthcare system by examining the Humber River Hospital in Toronto using the case study methodology. To achieve the desired benefits from the process of implementing technology in healthcare, our key findings show that hospitals need to undergo a business transformation that exploits technology. Finally, we conclude that only effective knowledge of tech-nology will enable hospitals to effectively become technological and digital.article:***class***class:

ChatGPT

**ChatGPT**

This article falls under the category of "Clinical Service Recommendation." It discusses the importance of implementing AI systems in healthcare to optimize resources, improve patient care, and enhance administrative processes. It emphasizes the need for hospitals to undergo a business transformation to effectively utilize technology. Therefore, it provides recommendations and insights into how healthcare institutions can leverage AI for the betterment of clinical services and healthcare management.

**Anonymous**

***article*** Global economic and social life has been severely challenged since the World Health Organization (WHO) declared the COVID-19 disease a pandemic. Travel, tourism and hospitality, in particular, has been massively impacted by the lockdowns used to maintain social distance to manage the disease. Robotics, artificial intelligence, and human-robot interactions have gained an increased presence to help manage the spread of COVID-19 in hospitals, airports, transportation systems, recreation and scenic areas, hotels, restaurants, and communities in general. Humanoid robots, autonomous vehicles, drones, and other intelligent robots are used in many different ways to reduce human contact and the potential spread of the SARS-CoV-2 virus, including delivering materials, disinfecting and sterilizing public spaces, detecting or measuring body temperature, providing safety or security, and comforting and entertaining patients. While controversial in the past due to concerns over job losses and data privacy, the adoption of robotics and artificial intelligence in travel and tourism will likely continue after the COVID-19 pandemic becomes less serious. Tourism scholars should seize this opportunity to develop robotic applications that enhance tourist experiences, the protection of natural and cultural resources, citizen participation in tourism development decision making, and the emergence of new 'high-touch' employment opportunities for travel, tourism and hospitality workers.article:***class***class:

ChatGPT

**ChatGPT**

This article primarily falls under the category of "Prescriptive Guidance." It discusses the increased use of robotics, artificial intelligence, and human-robot interactions in various sectors, particularly in response to the COVID-19 pandemic. It provides insights into how these technologies are being utilized to reduce human contact and manage the spread of the virus. Additionally, it suggests that the adoption of these technologies in travel and tourism will likely continue beyond the pandemic and encourages tourism scholars to develop applications that enhance various aspects of the tourism industry.

**Anonymous**

***article*** Drug development is time-consuming and expensive. Repurposing existing drugs for new therapies is an attractive solution that accelerates drug development at reduced experimental costs, specifically for Coronavirus Disease 2019 (COVID-19), an infectious disease caused by severe acute respiratory syndrome coronavirus 2 (SARS-CoV-2). However, comprehensively obtaining and productively integrating available knowledge and big biomedical data to effectively advance deep learning models is still challenging for drug repurposing in other complex diseases. In this review, we introduce guidelines on how to utilize deep learning methodologies and tools for drug repurposing. We first summarized the commonly used bioinformatics and pharmacogenomics databases for drug repurposing. Next, we discuss recently developed sequence-based and graph-based representation approaches as well as state-of-the-art deep learning-based methods. Finally, we present applications of drug repurposing to fight the COVID-19 pandemic and outline its future challenges. This article is categorized under: Data Science > Artificial Intelligence/Machine Learningarticle:***class***class:

ChatGPT

**ChatGPT**

This article falls under the category of "Prescriptive Guidance." It provides guidelines on how to utilize deep learning methodologies and tools for drug repurposing, particularly in the context of combating Coronavirus Disease 2019 (COVID-19). The article offers insights into the use of deep learning approaches, bioinformatics databases, and data integration for drug repurposing and discusses the challenges and future prospects in this field.
